# Supplementary material for: Vaginal probiotic adherence and acceptability in Rwandan women with high sexual risk participating in a pilot randomised controlled trial: a mixed-methods approach
Source: BMJ Open. 2020 May 19;10(5):e031819. doi: 10.1136/bmjopen-2019-031819 (PMC7247375; doi:10.1136/bmjopen-2019-031819)
Supplement: Supplementary data [file bmjopen-2019-031819supp001.pdf]

Supplementary Figure 1: Pictorial diary card

Date/Month	Descriptions	Monday	Tuesday	Wednesday	Thursday	Friday	Saturday	Sunday
Indicate each time you used study product								
	Used study product							
Indicate each sex act								
	Sex with condom							
	Sex without condom							
Indicate each time you washed/inserted something inside the vagina other than study product								
By washing inside, we mean inserting an entire finger inside the vaginal canal								
	Washed inside vagina with water only							
	Washed inside vagina with soap and water							
	Inserted something else (herbs, powders, etc.)							
Indicate each day of menstrual bleeding								
	Had menstrual bleeding							

The picture provided is the English translation of the pictorial card; participants received a version in Kinyarwanda.

Supplementary Table 1: Baseline characteristics of enrolled population

	Controls (n=17)	Metronidazole (n=17)	EF+ (n=17)	GynLP (n=17)
Median age (IQR)	29 (24–36)	30 (27–34)	33 (28–35)	30 (27–35)
Marital status n (%)				
- Never married	16 (94.1)	11 (64.7)	10 (58.8)	13 (76.5)
- Married	1 (5.9)	1 (5.9)	2 (11.8)	1 (5.9)
- Divorced	0	5 (29.4)	4 (23.5)	3 (17.6)
- Widowed	0	0	1 (5.9)	0
Education level n (%)				
- No schooling	5 (29.4)	3 (17.6)	3 (17.6)	3 (17.7)
- Primary school not completed	7 (41.2)	7 (41.2)	13 (76.5)	4 (23.5)
- Primary school completed	4 (23.5)	5 (29.4)	1 (5.9)	7 (41.2)
- At least some secondary school	1 (5.9)	2 (11.8)	0	3 (17.7)
Median number of sex partners last month (IQR)	5 (3–20)	5 (2–10)	3 (2–15)	3 (2–20)
Exchanged sex for money/goods past month n (%)	17 (100)	14 (82.4)	15 (88.2)	17 (100)
At least one laboratory-confirmed STI* n (%)	8 (47.1)	8 (47.1)	4 (23.5)	9 (52.9)
Median weekly frequency of washing body (IQR)	7 (7–7)	7 (7–7)	7 (7–7)	7 (4–7)
Ever washing the genitalia n (%)				
- Yes, outside only	12 (70.7)	14 (82.4)	15 (88.3)	14 (82.3)
- Yes, both inside and outside	5 (29.4)	3 (17.6)	2 (11.7)	3 (17.7)
- Yes, inside only	0	0	0	0
If reports washing inside, median weekly frequency (IQR)	14 (7–16)	14 (14–14)	11 (7–14)	7 (3–12)

*Chlamydia, gonorrhoea, and/or syphilis.

EF+, Ecologic Femi+; Enr, enrolment visit; GynLP, Gynophilus LP; IQR, inter-quartile range; M2, Month 2 visit; STI, sexually transmitted disease.

Supplementary Table 2: Acceptability of interventions

**Acceptability of study products at Enr**	**Controls (n=17)**	**Metronidazole (n=17)**	**EF+ (n=17)**	**GynLP (n=17)**
Nurse reports having explained intervention to participant in detail n (%)	17 (100)	17 (100)	17 (100)	17 (100)
Nurse reports participant asked questions n (%)*<br>- Yes, a few<br>- Yes, many	 6 (35.3)<br>0	 2 (11.8)<br>0	 11 (64.7)<br>0	 11 (64.7)<br>2 (11.8)
First dose applied† under supervision n (%)	NA	17 (100)	17 (100)	17 (100)
Median number of attempts participant made until successful application (IQR)	NA	NA	1 (1–1)	1 (1–1)
Participant seemed comfortable with the insertion after these attempts, according to study nurse n (%)<br>- Yes, very<br>- Yes, somewhat	NA	NA	 17 (100)<br>0	 16 (94.1)<br>1 (5.9)
**Acceptability of study products at M2**				
Self-reported usual time of insertion n (%)<br>- Before going to sleep<br>- After bathing in the morning	NA	NA	 17 (100)<br>0	 15 (100)‡<br>0
Level of comfort with vaginal insertion after 2 months of use, self-reported n (%)<br>- Very comfortable<br>- Somewhat comfortable	NA	NA	 17 (100)<br>0	 15 (100)‡<br>0
Reported insertion becoming easier over time n (%)	NA	NA	17 (100)	15 (100)‡
Reported manner of insertion§ n (%)<br>- While lying down<br>- While squatting	NA	NA	 17 (100)<br>1 (5.9)	 14 (93.3)‡<br>1 (6.7)
**Acceptability of penile hygiene intervention at M2**				
Reports having told main sex partner to regularly clean the penis, including underneath the foreskin n (%)¶<br>- Yes<br>- No, because he is circumcised<br>- No, other reason	 3 (17.7)<br>10 (58.8)<br>1 (5.9)	 3 (18.8)<br>9 (56.2)<br>0	 3 (17.6)<br>6 (35.3)<br>1 (5.9)	 3 (18.8)<br>5 (31.3)<br>1 (6.3)
If yes, response by the main partner n (%)‖<br>- He said that he would do so in the future<br>- He said that he already does this<br>- He said that he is not interested	 2 (66.7)<br>1 (33.3)<br>0	 1 (33.3)<br>1 (33.3)<br>1 (33.3)	 1 (33.3)<br>0<br>2 (66.7)	 1 (33.3)<br>1 (33.3)<br>1 (33.3)

*One missing value.
†Whether oral insertion (oral metronidazole group) or vaginal insertion (Ecologic Femi+ and Gynophilus LP groups).
‡N=15 due to participants withdrawing informed consent.
§Multiple answers possible; hence totals can be more than 100%.
¶Women with no main sex partner not included.
‖N=3 in all four groups.
EF+, Ecologic Femi+; Enr, enrolment visit; GynLP, Gynophilus LP; IQR, inter-quartile range; M2, Month 2 visit; NA, Not applicable.
