# Supplementary material for: Vaginal probiotic adherence and acceptability in Rwandan women with high sexual risk participating in a pilot randomised controlled trial: a mixed-methods approach
Source: BMJ Open. 2020 May 19;10(5):e031819. doi: 10.1136/bmjopen-2019-031819 (PMC7247375; doi:10.1136/bmjopen-2019-031819)
Supplement: Author's manuscript [file bmjopen-2019-031819.draft_revisions.pdf]

BMJ Open

BMJ Open is committed to open peer review. As part of this commitment we make the peer review history of every article we publish publicly available.

When an article is published we post the peer reviewers' comments and the authors' responses online. We also post the versions of the paper that were used during peer review. These are the versions that the peer review comments apply to.

The versions of the paper that follow are the versions that were submitted during the peer review process. They are not the versions of record or the final published versions. They should not be cited or distributed as the published version of this manuscript.

BMJ Open is an open access journal and the full, final, typeset and author-corrected version of record of the manuscript is available on our site with no access controls, subscription charges or pay-per-view fees (http://bmjopen.bmj.com).

If you have any questions on BMJ Open's open peer review process please email info.bmjopen@bmj.com

BMJ Open

Vaginal Probiotic Adherence and Acceptability in High-Risk Rwandan Women Participating in a Pilot Randomised Controlled Trial: A Mixed-Methods Approach

Journal:	*BMJ Open*
Manuscript ID	bmjopen-2019-031819
Article Type:	Original research
Date Submitted by the Author:	20-May-2019

Complete List of Authors:	Verwijs, Marijn; Institute of Infection and Global Health, University of Liverpool,<br>Agaba, Stephen; Rinda Ubuzima<br>Umulisa, Marie ; Rinda Ubuzima<br>Uwineza, Mireille; Rinda Ubuzima<br>Nivoliez, Adrien; Biose,<br>Lievens, Elke; Winclove<br>van de Wijgert, Janneke H.H.M.; University of Liverpool, Institute of Infection and Global Health; Universitair Medisch Centrum Utrecht, Julius Center for Health Sciences and Primary Care

Keywords:	bacterial vaginosis, vaginal probiotic, adherence, acceptability, Africa

SCHOLARONE™
Manuscripts

I, the Submitting Author has the right to grant and does grant on behalf of all authors of the Work (as defined in the below author licence), an exclusive licence and/or a non-exclusive licence for contributions from authors who are: i) UK Crown employees; ii) where BMJ has agreed a CC-BY licence shall apply, and/or iii) in accordance with the terms applicable for US Federal Government officers or employees acting as part of their official duties; on a worldwide, perpetual, irrevocable, royalty-free basis to BMJ Publishing Group Ltd ("BMJ") its licensees and where the relevant Journal is co-owned by BMJ to the co-owners of the Journal, to publish the Work in this journal and any other BMJ products and to exploit all rights, as set out in our licence.

The Submitting Author accepts and understands that any supply made under these terms is made by BMJ to the Submitting Author unless you are acting as an employee on behalf of your employer or a postgraduate student of an affiliated institution which is paying any applicable article publishing charge ("APC") for Open Access articles. Where the Submitting Author wishes to make the Work available on an Open Access basis (and intends to pay the relevant APC), the terms of reuse of such Open Access shall be governed by a Creative Commons licence – details of these licences and which Creative Commons licence will apply to this Work are set out in our licence referred to above.

Other than as permitted in any relevant BMJ Author's Self Archiving Policies, I confirm this Work has not been accepted for publication elsewhere, is not being considered for publication elsewhere and does not duplicate material already published. I confirm all authors consent to publication of this Work and authorise the granting of this licence.

For peer review only

Vaginal Probiotic Adherence and Acceptability in High-Risk Rwandan Women Participating in a Pilot Randomised Controlled Trial: A Mixed-Methods Approach

Authors: Marijn C. Verwijs (MD)[1], Stephen K. Agaba (MD)[2], Marie-Michele Umulisa (MA)[2], Mireille Uwineza (BA)[2], Adrien Nivoliez (PhD)[3], Elke Lievens (PhD)[4], Janneke H.H.M. van de Wijgert (MD PhD MPH)[1,5].

Author affiliations

[1]Institute of Infection and Global Health, University of Liverpool, Liverpool, United Kingdom.

[2]Rinda Ubuzima, Kigali, Rwanda.

[3]Biose, Aurillac, France.

[4]Winclove Probiotics, Amsterdam, The Netherlands.

[5]Julius Center for Health Sciences and Primary Care, University Medical Center Utrecht, Utrecht University, Utrecht, The Netherlands.

Correspondence to

Professor Janneke van de Wijgert, MD PhD MPH

Department of Clinical Infection, Microbiology and Immunology

Institute of Infection and Global Health, University of Liverpool

Ronald Ross Building, 8 West Derby Street, Liverpool L69 7BE, United Kingdom

Email: j.vandewijgert@liverpool.ac.uk

ORCID: 0000-0003-2728-4560

Word counts:

Word count abstract: 300 words (max. 300 words).

Word count main text: 3,479 words (max. 4000 words).

Number of tables and figures: 5 (max. 5).

For peer review only - http://bmjopen.bmj.com/site/about/guidelines.xhtml

For peer review only

Number of supplementary tables and figures: 3.

For peer review only

For peer review only - http://bmjopen.bmj.com/site/about/guidelines.xhtml

ABSTRACT

Objectives Bacterial vaginosis (BV) recurrence is common. We evaluated the adherence and acceptability of intermittent use of two vaginal probiotics and one antibiotic to prevent recurrence.

Design Repeated adherence and acceptability assessments using mixed methods within a pilot randomised controlled trial.

Setting Research clinic in Kigali, Rwanda.

Participants High-risk Rwandan women (n=68) with BV and/or trichomoniasis.

Interventions Women were randomised to four groups (n=17 each) after completing metronidazole treatment: behavioural counselling only, or behavioural counselling plus two-month intermittent use of oral metronidazole, Ecologic Femi+ (EF+) vaginal capsule, or Gynophilus LP (GynLP) vaginal tablet.

Outcome measures Adherence and acceptability data from randomised women were collected in structured face-to-face interviews, semi-structured focus group discussions and in-depth interviews, daily diaries, and counting of used/unused study products. Randomised women and women attending recruitment sessions (n=131) were surveyed about vaginal infection knowledge.

Results Most women (93%) were sex workers. At baseline, they were unfamiliar with BV, and had never used probiotics. All probiotic users reported that insertion became easier over time. Triangulated adherence data showed that 100% of EF+ users and 88.2% of GynLP users used ≥80% of required doses. Younger age, asking many questions at enrolment, having menses, and reporting urogenital symptoms showed non-significant trends towards a lower perfect adherence likelihood. Qualitative data suggested that women believed that the probiotics reduced BV recurrence, but that partners were sometimes unsupportive of study participation. Self-reported vaginal washing practices decreased during follow-up, but sexual risk behaviours did not. Most women (80%) with an uncircumcised steady partner discussed penile hygiene with him, but many women found this difficult, especially with male clients.

Conclusions High-risk women require education about vaginal infections. Vaginal probiotic acceptability and adherence were high in this cohort. Our results can be used to inform future product development and to fine-tune counselling messages in prevention programs.

For peer review only - http://bmjopen.bmj.com/site/about/guidelines.xhtml

For peer review only

Trial registration ClinicalTrials.gov (NCT02459665).

Keywords (5) bacterial vaginosis, vaginal probiotic, adherence, acceptability, Africa.

ARTICLE SUMMARY

Strengths and limitations of this study

- We conducted this research in the context of a pilot randomised controlled trial, and statistical power was therefore limited.
- We triangulated different sources of adherence data to maximise accuracy, and used a mixed-methods approach to evaluate acceptability.
- We could not directly compare experiences with, and opinions about, the two different vaginal probiotics because each woman used only one product and qualitative data depth was suboptimal.
- Social desirability bias may have affected some of the results.
- The results of this study may not be generalizable to women at lower risk of HIV/STIs.

For peer review only

For peer review only - http://bmjopen.bmj.com/site/about/guidelines.xhtml

INTRODUCTION

Bacterial vaginosis (BV) is a vaginal condition in which fastidious anaerobes such as *Gardnerella vaginalis* increase while beneficial, lactic acid-producing lactobacilli decrease.[1] Often asymptomatic, it is associated with increased risks of sexually transmitted infections (STIs) and HIV transmission, pelvic inflammatory disease, and adverse pregnancy outcomes.[2–5] Although BV is treatable with antibiotics, the risk of recurrence is high.[6,7] The prevalence of BV varies among regions and ethnic groups but is highest in sub-Saharan Africa, where it is estimated at 30-50%.[8]

Vaginally-administered probiotics containing lactobacilli are considered a promising new strategy to restore a lactobacilli-dominated vaginal microbiota during and/or after antibiotic treatment, or to prevent BV.[9] While some probiotics have been available on the market for several years, clinical trials to support beneficial effects have only recently been initiated for most products.[10–13] Acceptability is an important component of these trials, to maximise future uptake and adherence of vaginal probiotics should they be proven efficacious. The acceptability of a novel vaginal product depends on factors such as the characteristics of the population studied, characteristics of and experiences with the product, types of sexual relationships and partner support, and community perceptions.[14,15]

We conducted a clinical trial of intermittent use of two vaginal probiotics and oral metronidazole to prevent BV recurrence in Rwandan women who had been treated for BV and/or *Trichomonas vaginalis* (TV). We used qualitative and quantitative research methods to assess adherence and acceptability with vaginal probiotic use. We triangulated various sources of adherence data to obtain adherence estimates per woman for each period of intermittent product use in between study visits, and determined correlates of adherence.

METHODS

The pilot clinical trial took place from June 2015 to February 2016 at the Rinda Ubuzima research clinic in Kigali, Rwanda. Women who had been successfully treated for BV/TV with a seven-day

For peer review only

course of oral metronidazole (Tricozole, Laboratory & Allied Ltd, Nairobi, Kenya) were randomised to four intervention groups (n=17 each) to prevent BV recurrence: behavioural counselling only (controls), or behavioural counselling plus intermittent use of two different vaginal probiotics or oral metronidazole for two months. Women were seen at screening, enrolment (product use initiation, if applicable), Day 7, Month 1, Month 2 (product use cessation, if applicable), and Month 6. Product efficacies were not known during the trial, and preliminary efficacy results are reported elsewhere.[16] The behavioural counselling focussed on safer sex practices, cessation of vaginal practices, and increasing male penile hygiene to prevent BV.

Study population

Women aged 18-45 at risk of HIV/STIs (defined as having had more than one sex partner and/or having been treated for an STI and/or BV in the last 12 months) were eligible for enrolment if they were confirmed HIV-negative, non-pregnant, diagnosed with BV and/or TV, and cured after seven-day oral metronidazole treatment. Other clinical exclusion criteria were applied but were rare.[16] Women were recruited by study staff with the assistance of Community Mobilisers who had strong ties with local high-risk women (particularly sex workers).

Study products and dosing

Ecologic Femi+ (EF+; Winclove Probiotics, Amsterdam, Netherlands) is a vaginal capsule containing lyophilised lactic acid-producing bacteria. EF+ was used once per day for five days followed by thrice weekly, for two months. Gynophilus LP (GynLP; Biose, Aurillac, France) is a tablet containing the *Lactobacillus rhamnosus* Lcr35 strain. The tablet disintegrates in the vagina and forms a gel that slowly releases the probiotic bacteria. GynLP was used once every four days for two months. The first dose was inserted at the clinic under direct observation of a clinician, and remaining doses were self-administered at home. Women were asked not to cleanse or insert other products into the vagina after probiotic insertion to allow the probiotics to dissolve. They were also told that they were allowed to cease probiotic use during menses, but were encouraged to continue. Intermittent metronidazole use was chosen as a positive control intervention because studies conducted in the U.S. and Kenya have

For peer review only - http://bmjopen.bmj.com/site/about/guidelines.xhtml

shown a 30-40% reduction in BV recurrence.[17,18] Metronidazole users took 500 mg generic oral metronidazole (Laboratory & Allied ltd, Nairobi, Kenya) twice weekly for two months. Participants and clinicians were not blinded.

Acceptability, adherence, behavioural, and vaginal infection knowledge assessments

Acceptability was assessed at the enrolment visit prior to product use initiation and at the Month 2 visit after the full two months of use. Adherence was assessed during the intervention period, at the Day 7, Month 1, and Month 2 visits. Sexual and other behaviours were assessed at all study visits. Participants were interviewed face-to-face in Kinyarwanda by a trained study nurse using structured questionnaires with multiple-choice questions, questions requiring a number or date, and an adherence self-rating scale (from 0-10). In between visits, participants used pictorial diary cards (online supplementary material figure 1) to record daily episodes of product use, vaginal sex, condom use, and vaginal practices. Those using study products returned the product packaging and unused products (if applicable) to their clinic visits, where they were counted by study staff. Any discrepancies between data sources were discussed with participants, and consensus, triangulated assessments of adherence were recorded on the questionnaires. Additionally, 131 women were interviewed about their knowledge of vaginal infections (such as BV and STIs) using a structured questionnaire during recruitment sessions (n=61; regardless of eligibility) and at enrolment visits (n=70; this included the 68 randomised women, and two women who attended enrolment visits but turned out to be ineligible). Women were interviewed before being counselled at study visits or before receiving information at recruitment sessions. This questionnaire contained multiple-choice and open-ended questions. Responses to the open-ended questions were categorised and discussed by two different researchers until consensus about the answer categories was reached.

Four semi-structured focus group discussions (FGDs) with 7-11 participants per group (total n=38), and four semi-structured individual in-depth interviews (IDIs) were held with enrolled participants, about their experiences with and opinions of the products, sexual behaviour, and vaginal practices. Women randomised to the behavioural counselling only group were not included in the FGDs and

For peer review only

IDIs. All had completed their product use period. The interviews were unlinked anonymous, and women used pseudonyms to enable them to talk freely despite the fact that the discussions and interviews were taped. All interviews took place between November 2015 and March 2016, were held in Kinyarwanda, recorded on tape, transcribed verbatim, and translated into English.

Data analysis

Questionnaire data were analysed using Stata 13 (StataCorp, College Station, TX, USA). The proportion of women with ≥80%/≥90%/100% adherence in the probiotic groups were compared by Fisher's exact tests. Changes in self-reported vaginal practices and sexual behaviours over time were tested using McNemar's test for binary outcomes, and Wilcoxon's signed-rank test for continuous outcomes. To study associations of participant characteristics with triangulated adherence, we used bivariable mixed effects models, with perfect adherence (defined as having used all doses as instructed) per interval between study visits during the intervention period as the outcome, participant identification numbers as the random effect, and one participant characteristic at the time as the fixed effect. We could not determine correlates of acceptability due to limited variation in the acceptability data (reported acceptability was high throughout the trial).

The FGD and IDI transcripts were read and discussed by three researchers (MV, MU, and JvdW). The Chief Investigator (JvdW) decided that data saturation had been met when the fourth FGD and the fourth IDI transcript had become available in March 2016. The transcripts were then coded using NVivo 10.0 (QSR International, Melbourne, Australia) by one single researcher (MV). The discussions and interviews were semi-structured, with themes and associated codes prepared a priori, as well as new elements that emerged from the data. The codes were derived from an acceptability framework that has been used in studies of vaginal products for contraception or HIV prevention.[14,15,19] Components of the framework include study population characteristics, product attributes, sexual encounter and relational attributes, and the contextual environment (e.g. community perceptions of product use).

For peer review only - http://bmjopen.bmj.com/site/about/guidelines.xhtml

Ethical statement

All participants provided written consent for study participation, and separate consent for participation in FGDs/IDIs. All non-married participants aged 18-20 also required parental/guardian consent per Rwandan law at the time of the study. The participants received 3 GBP per visit (in local currency) as a reimbursement for time and transport costs. Care was taken to protect participant privacy and confidentiality. The study was sponsored by the University of Liverpool, approved by the Rwanda National Ethics Committee and the University of Liverpool Research Ethics Subcommittee for Physical Interventions, and registered on ClinicalTrials.gov (NCT02459665).

Participant and public involvement

A subset of the enrolled participants were invited to comment on study design and experiences with the interventions during the FGDs/IDIs. Participants were not invited to develop outcomes, interpret the results, or to contribute to the writing or editing of this document for readability or accuracy. The preliminary results of this study were discussed with 32 stakeholders during a workshop held at the Ministry of Health in Kigali, Rwanda, in December 2017. These stakeholders included representatives of the Ministry of Health, the National University of Rwanda, the National Ethics Committee, local hospitals and clinics, and local non-governmental and women's organisations.

RESULTS

Baseline characteristics

We screened 176 women: bacterial STI prevalence was 31.3% and BV prevalence by Gram stain Nugent scoring was 47.9%. All 68 randomised women were at risk of STI/HIV transmission, with 93.1% reporting having exchanged sex for money and/or goods in the previous month (figure 1, online supplementary material table 1). We collected 29.93 person-years of data. Four women withdrew their informed consent during the study (for reasons unrelated to study product acceptability). None were lost to follow-up.

For peer review only

For peer review only - http://bmjopen.bmj.com/site/about/guidelines.xhtml

Adherence

Triangulated adherence was high: 100% of EF+ users and 88.2% of GynLP users used ≥80% of required doses (Fisher's exact p=0.103; table 1), and these percentages were 88.2% and 68.8% for ≥90% (p=0.225), and 58.8% and 50% for 100% of required doses (p=0.732), respectively. In comparison, these percentages were 88.2%, 82.4%, and 70.6%, respectively, for oral metronidazole users. Reported reasons of non-adherence to vaginal probiotics during face-to-face interviews were 'simply forgetting' (n=9), experiencing side-effects (n=2), menses (n=2), and being away from home and having left products at home (n=1). Additional reasons for missing doses mentioned during FGDs/IDIs were being drunk (n=2) and being confused about the dosing schedule (n=2). Only one woman in the metronidazole arm reported missing doses due to experiencing side-effects. Most women in FGDs reported using all doses as instructed and finding it easy to adhere, and thought that the diary cards served as a useful reminder to use the products.

For peer review only

For peer review only - http://bmjopen.bmj.com/site/about/guidelines.xhtml

Table 1: Adherence to study interventions

Adherence to study products	Metronidazole (n=17)	EF+ (n=17)	GynLP (n=16)
Adherence Enr–D7, median % (IQR)	100 (100–100)	100 (100–100)	100 (100–100)
Adherence D7–M1, median % (IQR)	100 (100–100)	100 (100–100)	100 (91.7–100)
Adherence M1–M2, median % (IQR)	100 (100–100)	100 (100–100)	100 (92.3–100)
Overall adherence Enr–M2, median % (IQR)	100 (96.3–100)	100 (100–100)	98.3 (89.3–100)
Overall adherence Enr–M2 n (%)<br>- Perfect*<br>- Adherence ≥90%<br>- Adherence ≥80%	 12 (70.6)<br>14 (82.4)<br>15 (88.2)	 10 (58.8)<br>15 (88.2)<br>17 (100)	 8 (50.0)<br>11 (68.8)<br>13 (81.3)
Number of times menses Enr–M2 n (%)†<br>- Never<br>- Once<br>- Twice	 7 (41.2)<br>6 (35.3)<br>4 (23.5)	 4 (23.5)<br>5 (29.4)<br>8 (47.1)	 2 (12.5)<br>4 (25.0)<br>10 (62.5)
Did not use product during menses at least once n (%)<br>- Yes<br>- NA (never had menses)	 4 (23.5)<br>7 (41.2)	 3 (17.6)<br>4 (23.5)	 5 (31.3)<br>2 (12.5)
**Self-reported reasons for non-adherence‡**	**Metronidazole**	**EF+**	**GynLP**
D7: Self-reported reasons why not able to use all doses as instructed n (%)§<br>- Simply forgot<br>- Product had side effects	 0<br>0	 2 (11.8)<br>0	 0<br>1 (6.7)¶
M1: Self-reported reasons why not able to use all doses as instructed n (%)§<br>- Simply forgot<br>- Product had side effects<br>- Did not like product for another reason<br>- Other	 1 (6.3)<br>1 (6.3)‖<br>1 (6.3)‖<br>1 (6.3)**	 1 (5.9)<br>0<br>0<br>1 (5.9)††	 1 (6.3)<br>1 (6.3)‡‡<br>0<br>2 (12.5)§§
M2: Self-reported reasons why not able to use all doses as instructed n (%)§<br>- Simply forgot<br>- Travelled and forgot to take product<br>- Other	 1 (6.3)<br>1 (6.3)<br>0	 2 (11.8)<br>0<br>1 (5.9)¶¶	 3 (18.8)<br>1 (6.25)<br>1 (6.3)‖‖
D7: Participant thinks she used product correctly most of the time n (%)	17 (100)	16 (94.1)	14 (93.3)
M1: Participant thinks she used product correctly most of the time n (%)	13 (86.7)	17 (100)	11 (68.8)
M2: Participant thinks she used product correctly most of the time n (%)	15 (93.7)	16 (94.1)	14 (87.5)

*Defined as 100% of the prescribed doses used at the prescribed times after nurse review of the participant's diary card and returned used packaging and unused product.
†Number of times menses in the control group: never 2 (11.8%), once 3 (17.8%), twice 11 (64.7%), and thrice 1 (5.9%).
‡Numbers of participants per randomisation group may very slightly due to loss to follow-up. Participants with ≥90% adherence not shown.
§Multiple answers possible.
¶Participant reported vulval itching and burning when passing urine.
‖Participant reported mild gastritis and wanting to withdraw from the study anyway.
**Participant reported receiving oral metronidazole therapy for 7 days due to infection.
††Participant reported having menses twice in one month; decided to use less of her product until the next study visit.
‡‡Participant reported genital itching, genital burning, and pain during sex.
§§One participant reported missing the D7 study visit and therefore running out of supplies. Another participant reported not to have used the study product during menses (which she was allowed to do).
¶¶Participant reported being drunk and therefore forgetting to take the study product.
‖‖Participant reported taking the study product correctly but that the product came out during menses
D7, Day 7 visit; EF+, Ecologic Femi+; Enr, enrolment visit; GynLP, Gynophilus LP; IQR, inter-quartile range; M1/2, Month 1/2 visit; NA, not applicable.

For peer review only - http://bmjopen.bmj.com/site/about/guidelines.xhtml

Acceptability

Ease-of-use

No participants reported having heard about probiotics before study participation. After product use, all vaginal probiotic users reported feeling very comfortable with insertion and that insertion became easier over time. All but one woman reported inserting while lying down (online supplementary material table 2).

Bodily changes and product perception

During FGDs, several women using either vaginal probiotic reported the product (partially) "*coming out*" during the first few uses, but that this decreased after having gained experience. Many EF+ and GynLP users reported an increase in vaginal wetness, which was considered a positive attribute by most. Some women reported increased libido. For example, one EF+ user said: "*I felt a great desire to* [have] *sex again and again.*" In contrast, one metronidazole user reported a decrease in libido. Most women believed that the vaginal probiotics decreased the recurrence of symptomatic BV (our preliminary efficacy data suggest that BV incidence had in fact decreased),[16] and a few believed that they also prevented STI acquisition (the trial had insufficient statistical power to assess this).

Support

One social harm related to vaginal probiotic use was reported: a GynLP user was verbally harassed by her partner and her sister because of her study participation, and opted to withdraw her informed consent. Reports of partner, family, and community support during the FGDs/IDIs were mixed: some women reported problems with loved ones. Negative reactions from male partners were more often based on suspicions about study participation than the products themselves. One EF+ user said: "*He* [her partner] *did not accept that. He asked me to go together with him to the clinic* [a local health centre] *and check if I am not HIV-positive.*" Another participant using metronidazole mentioned wanting to join the study to her husband, who forbade her to participate. However, she decided to join anyway: "*he did not know that I was using the study product, because he had refused me to join* [the]

For peer review only - http://bmjopen.bmj.com/site/about/guidelines.xhtml

study before… I used them [the study products] *without informing him.*" All sex workers except one stated that they had not discussed study participation with male clients.

Worries and concerns

In the FGDs, one woman reported hearing rumours prior to enrolling that vaginal products "*can damage the uterus or cause tumours in the womb.*" However, most participants thought that vaginal probiotics would be acceptable to Rwandan women. One GynLP user argued: "*They* [already] *give us vaginal pills*", by which she meant vaginal medications for yeast infections. Some women were concerned about future product availability and pricing. They hoped that probiotics would be distributed cheaply through the Rwandan *Mutuelle* public health insurance because they would otherwise be inaccessible to many women. One metronidazole user was concerned about a limited applicability of probiotics because BV is not diagnosed by laboratory testing in Rwanda: "*They do not have adequate medical instruments to test diseases, you tell the physician how* […] *you feel and by guessing the disease, he gives you at least four medications, saying that you may have trichomonas, you may have syphilis, you may have gonorrhoea* [she refers to syndromic management.[20,21]] *At health centre-level they do not have medical equipment to test diseases, meaning that they will not know who to give that* [probiotic/antibiotic maintenance therapy] *medication.*"

Vaginal practices and sexual risk-taking

At enrolment, 49.3% of the women reported to never use products inside the vagina, and at Month 6, this increased to 81.5% (OR 5.2, 95% CI 1.96-17.34; table 2). During FGDs, some women understood that vaginal washing practices may increase the risk of vaginal infection, but others did not. A participant stated: "*You get them* [i.e., vaginal diseases] *anyway… whether you wash or not*". In one FGD, 10 of 11 participants stated having ceased vaginal practices thanks to the study counselling. It should be noted that in contrast to many other African populations, Rwandan women use vaginal practices to increase rather than reduce vaginal lubrication. Women mentioned the use of herbs (*umushishiro*), Vaseline, and oils for this purpose. Self-reported sexual risk taking by face-to-face interview did not change over time, except for a significant reduction in reported numbers of sex

For peer review only - http://bmjopen.bmj.com/site/about/guidelines.xhtml

296 partners in the previous month at Month 6 compared to enrolment. No women in FGDs/IDIs
297 mentioned adopting safer sex practices (such as consistent condom use) in response to the counselling
298 messages. During face-to-face interviews at the Month 2 visit, 12 of 15 women (80%) who had an
299 uncircumcised main sex partner reported asking him to regularly clean his penis in the future (online
300 supplementary material table 2). While most women in FGDs understood that using condoms and
301 improved penile hygiene could reduce BV rates, some mentioned that they found it difficult to discuss
302 these topics with male partners. One participant stated that this is especially difficult being a sex
303 worker: "*a man gives you his own money and you start educating him to wash!*" However, another
304 sex worker reported refusing sex with uncircumcised clients: "*you leave him, because he has a lot* [of]
305 *germs*". Several women reported discussing circumcision with their partners; one participant reported
306 telling her husband: "*It is better that you do circumcision because it is a good thing… you would get a*
307 *chance of not contracting diseases.*"

For peer review only

For peer review only - http://bmjopen.bmj.com/site/about/guidelines.xhtml

Table 2: Changes in reported vaginal cleansing practices and (sexual) behaviour between the enrolment and the M6 visit.

Self-reported sociodemographic characteristics	Enr (n=71)	M6 (n=65)	OR (95% CI)*	P value*
Reports using no products inside the vagina (other than for managing menses; all participants) n (%)	35 (49.3)	53 (81.5)	5.2 (1.96–17.34)	<0.001
Reports using no products inside the vagina (other than for managing menses; controls and metronidazole users only)† n (%)	15 (44.1)	27 (79.4)	13.0 (1.95–552.5)	0.002
Reports using water only n (%)	23 (32.4)	10 (15.4)	0.37 (0.13–0.92)	0.029
Reports using water and soap n (%)	3 (4.2)	2 (3.1)	0.67 (0.06–5.82)	1.00
Reports using paper, cloth or cotton wool n (%)	9 (12.7)	0 (0)	0.13 (0.00–0.93)‡	0.008
Reports using traditional herbs, stones, powders as vaginal cleansing practice n (%)	1 (1.4)	1 (1.5)	1.00 (0.01–78.5)‡	1.00
Mean weekly frequency of vaginal practices (95% CI)	2.15 (0.97–3.34)	0.64 (0.18–1.11)	NA	0.328
Median number of sex partners in last month at baseline or per month during follow-up period (IQR)	5 (3–16)	2 (1–4)	NA	<0.001
Any condom use reported in past two weeks (Enr) or since last study visit (M6), versus none n (%)	64 (90.1)	60 (92.3)	1.67 (0.32–10.7)	0.727
Reports exchanging sex for money/goods in past month (Enr) or since last study visit (M6) n (%)	65 (91.5)	58 (89.2)	0.80 (0.16–3.72)	1.00

*McNemar's OR and p-value for binary variables and Wilcoxon signed-rank test p-value for continuous variables, comparing the response at M6 with the response at Enr. ORs with 95% CI were also calculated for binary pre-post data.
†N=34.
‡To enable calculation of effect measures, a zero value was replaced by 1.
CI, confidence interval; Enr, enrolment visit; IQR, inter-quartile range; M6, Month 6 visit; NA, not applicable; OR, odds ratio.

Correlates of adherence

In bivariable mixed effects models including the probiotic groups only, no participant characteristics were significantly associated with perfect adherence (table 3). However, non-significant trends were observed. Younger age, asking many questions at enrolment, having menses during the previous study interval, and reporting urogenital symptoms were associated with a lower likelihood of perfect adherence. When including oral metronidazole users, menses was significantly associated with a lower likelihood of perfect adherence (p=0.008). There were no significant associations between randomisation group and perfect adherence.

For peer review only

For peer review only - http://bmjopen.bmj.com/site/about/guidelines.xhtml

Table 3: Participant characteristics associated with perfect adherence

Participant characteristics	EF+ and GynLP users		EF+, GynLP and oral metronidazole users	
	OR (95% CI)	P value	OR (95% CI)	P value
Randomisation group: GynLP versus EF+	0.68 (0.22–2.11)	0.505	ND	ND
Randomisation group:<br>- EF+ versus metronidazole<br>- GynLP versus metronidazole	ND	ND	 0.53 (0.15–1.81)<br>0.36 (0.11–1.23)	 0.308<br>0.103
Age in years: ≥30 years versus <30	2.66 (0.90–7.82)	0.076	1.60 (0.61–4.15)	0.336
Marital status:<br>- Married versus never married<br>- Divorced versus never married<br>- Widowed versus never married	 0.97 (0.14–6.58)<br>1.18 (0.29–4.79)<br>ND	 0.976<br>0.912<br>0.991	 1.17 (0.20–6.99)<br>1.39 (0.42–4.57)<br>ND	 0.865<br>0.586<br>0.990
At least some schooling versus no schooling	1.20 (0.59–2.45)	0.619	0.80 (0.22–2.95)	0.740
Number of sex partners last month: five or more versus four or less.	0.58 (0.18–1.83)	0.351	0.49 (0.17–1.37)	0.173
Exchanged sex for money/goods past month	ND	0.990	ND	0.986
Nurse reported participant asked questions at Enr<br>- Yes, many versus none<br>- Yes, a few versus none	 0.19 (0.02–1.52)<br>0.83 (0.24–2.83)	 0.116<br>0.761	 0.15 (0.02–1.19)<br>0.83 (0.27–2.57)	 0.072<br>0.744
Had menses during study visit interval	0.41 (0.14–1.20)	0.104	0.26 (0.09–0.70)	0.008
Reported alcohol consumption during study:<br>- Once or twice per week versus never<br>- More than twice per week versus never	 0.54 (0.14–2.12)<br>0.92 (0.18–4.81)	 0.373<br>0.920	 0.34 (0.11–1.08)<br>0.81 (0.19–3.49)	 0.068<br>0.774
Reported at least one urogenital symptom during study interval versus none	0.11 (0.01–1.56)	0.103	0.30 (0.04–2.16)	0.231
Reported at least one adverse event during study visit interval (excluding urogenital symptoms) versus none	0.43 (0.10–1.83)	0.253	0.55 (0.15–2.05)	0.371

Sociodemographic characteristics associated with perfect adherence in bivariable mixed effects models, in the enrolment–D7, D7–M1, and M1–M2 study visit intervals.
CI, confidence interval; D7, Day 7 visit; EF+, Ecologic Femi+; Enr, enrolment visit; GynLP, Gynophilus LP; M1, Month 1 visit; M2, Month 2 visit; ND, non-determinable; OR, odds ratio.

Vaginal infection knowledge

Almost all participants reported having heard of 'diseases of the vagina' and STIs before, but only 4.6% knew what bacteria were (table 4). The STIs most often spontaneously named (in numerical order) were HIV, gonorrhoea, and syphilis; only one participant reported having heard of BV. After having received an explanation about what BV is, only one woman reported ever having been diagnosed with BV. Most participants could name at least one cause or potential consequence of vaginal infections. Consequences wrongfully attributed to vaginal infections were death (4.6% of women), infant malformations (3.9%), and cervical cancer/tumours (3.1%).

For peer review only

For peer review only - http://bmjopen.bmj.com/site/about/guidelines.xhtml

339 **Table 4: Vaginal infection knowledge**

340

	Recruitment (n=61)	Enrolment (n=70)	Total (n=131)
Median age (IQR)	32 (27–35)*	31 (27–35)	31 (27–35)
Has heard of diseases of the vagina before n (%)	60 (98.4)	70 (100)	130 (99.2)
Reports knowing what bacteria are before study n (%)	5 (8.2)	1 (1.4)	6 (4.6)
Reports having heard about STIs before study n (%)	61 (100)	70 (100)	131 (100)
If yes, spontaneously named, without probing† n (%)			
- HIV	58 (95.1)	65 (92.9)	123 (93.9)
- Gonorrhoea	58 (95.1)	65 (92.9)	123 (93.9)
- Syphilis	44 (72.1)	59 (84.3)	103 (78.7)
- Trichomoniasis	38 (62.3)	48 (68.6)	86 (65.7)
- Hepatitis	3 (4.9)	3 (4.3)	6 (4.6)
- Yeast infection	0	3 (4.3)	3 (2.3)
- BV	0	2 (2.9)	2 (1.5)
- Urinary tract infection	1 (1.6)	1 (1.4)	2 (1.5)
- Chlamydia	0	1 (1.4)	1 (0.8)
- Herpes	0	1 (1.4)	1 (0.8)
- HPV / cervical cancer	1 (1.6)	0	1 (0.8)
Reports having heard about BV before this study n (%)	1 (1.6)	0	1 (0.8)
Spontaneously reported reasons why women get vaginal disease, without probing† n (%)			
- Poor toilet hygiene	37 (60.7)	40 (57.1)	77 (58.8)
- Multiple sex partners	28 (45.9)	36 (51.4)	64 (48.9)
- After sex	25 (41.0)	30 (43.0)	55 (42.0)
- Dirty underwear	19 (31.2)	35 (50.0)	54 (41.2)
- Poor vaginal hygiene	26 (42.6)	22 (31.4)	48 (36.6)
- Poor penile hygiene of male partner(s)	4 (6.6)	17 (24.3)	21 (16.0)
- Traditional vaginal practices and washing	3 (4.9)	12 (17.1)	15 (11.5)
- New sex partner	6 (9.8)	3 (4.3)	9 (6.9)
- Use of contraception	1 (1.6)	3 (4.3)	4 (3.1)
- (Improper) use of sanitary pads or tampons	1 (1.6)	3 (4.3)	4 (3.1)
- Other	3 (4.9)‡	1 (1.4)§	4 (3.1)
- Cannot name any reasons	1 (1.6)	0	1 (0.8)
Spontaneously reported negative consequences of vaginal disease being named, without probing† n (%)			
- Foul smell from the vagina	30 (49.2)	39 (56.5)	69 (53.1)
- Difficulty getting pregnant	18 (29.5)	33 (47.8)	51 (39.2)
- Miscarriage	16 (26.2)	33 (47.8)	49 (37.7)
- Abnormal vaginal discharge	12 (19.7)	28 (40.6)	40 (30.8)
- Baby born too early	16 (26.2)	22 (31.9)	38 (29.2)
- Severe infection / fever of the woman	7 (11.5)	7 (10.1)	14 (10.8)
- Infection / fever of the newborn baby	5 (8.2)	3 (4.4)	8 (6.2)
- Itching	4 (6.6)	4 (5.8)	8 (6.2)
- Other consequences to the baby	3 (4.9)	3 (4.4)	6 (4.6)
- Cervical cancer or tumours	2 (3.3)	3 (4.4)	5 (3.9)
- Death	4 (6.6)	0	4 (3.1)
- HIV/STIs	1 (1.6)	3 (4.4)	4 (3.1)
- Pain during intercourse	0	3 (4.4)	3 (2.3)
- Cannot name any consequence	17 (27.9)	19 (27.5)	36 (27.7)

341 *One missing value.

342 †Open-ended question. Totals may be more than 100%.

343 ‡Participants report: “If you are infected with STIs”, sharing underwear, and unprotected sex.

344 §Participant reports: vaginal medicine.

345 BV, bacterial vaginosis; HPV, human papilloma virus; IQR, interquartile range; STI, sexually transmitted infection.

For peer review only

For peer review only - http://bmjopen.bmj.com/site/about/guidelines.xhtml

DISCUSSION

Several studies of different vaginal probiotics have been conducted, some of them in sub-Saharan Africa.[10–13] However, none reported in-depth acceptability and adherence data. Our study suggests high vaginal probiotic acceptability and adherence in high-risk Rwandan women. We found no statistically significant correlates of perfect adherence, partially due to limited statistical power, but younger age, asking many questions about product use at enrolment, current menses, and reporting urogenital symptoms showed trends towards a lower likelihood of perfect adherence. Vaginal probiotics are currently unavailable on the market in most African countries, and it is important to study acceptability in different target populations to inform product development and future marketing strategies.

We could not evaluate the impact of self-reported acceptability aspects on adherence because almost all women reported very high acceptability in face-to-face interviews throughout the trial. Such interviews are known to suffer from social desirability bias. However, women seemed to speak freely in the FGDs, and those data indicate that they did not have major issues with product attributes or insertion. However, some women reported difficulties due to lack of male partner support. The reported increase in vaginal wetness after probiotic insertion was not considered problematic, as lubrication during sex is preferred by most Rwandan men and women.[22] This might be different in other countries where dry sex is preferred.[23] We did find a non-significant lower adherence to GynLP compared to EF+, which might be explained by differences in formulation: GynLP forms a gel in the vagina whereas EF+ capsules merely release lyophilised bacteria. Previous research indicated high adherence to GynLP.[24] Unfortunately, the impact of these formulation differences was insufficiently probed during the FGDs. Participants indicated that they found the diary cards helpful in reminding them to use their products, and we believe that self-monitoring tools might indeed be helpful in maximising adherence.[25]

Our data suggest that counselling was partially effective in changing behaviours that increase BV risk. Significantly more women reported not engaging in vaginal practices at the end of the study, and most

For peer review only - http://bmjopen.bmj.com/site/about/guidelines.xhtml

women with uncircumcised steady male partners reported having discussed penile hygiene with them. However, many women mentioned in FGDs that they found it difficult to discuss condom use and penile hygiene with male partners, especially clients. Women reduced their sexual risks only to a limited extent during follow-up, reporting a reduction in numbers of sex partners but no differences in engaging in sex work and condom use in face-to-face interviews. While these results are encouraging, it is difficult to assess to what extent they were influenced by social desirability bias.

Our survey with women at recruitment sessions and enrolment visits showed that high-risk Rwandan women had heard of several STIs, but were generally unaware of BV, its causes and potential consequences, and what they can do to prevent it. Experiences with HIV show that public health interventions can only succeed if health care professionals and the public have sufficient knowledge of causes and consequences of disease.[26–28] High-risk Rwandan women (and health care professionals) should therefore be educated about BV.

Limitations

Our study had limited statistical power, and social desirability bias may have affected some of our results, as is often the case in studies of this nature. Additionally, it should be noted that product efficacy, availability and cost are important determinants of acceptability, and were not evaluated in our study, although preliminary efficacy results in this study were promising.[16] We could not directly compare experiences with, and opinions about, the two different vaginal probiotics because each woman used only one product and qualitative data depth was suboptimal. In the FGDs/IDIs, it was sometimes difficult to ascertain whether participants were referring to personal experiences, or to wider community perceptions. Strengths of our study include the use of a mixed-methods approach and triangulated adherence data.

CONCLUSIONS

The prevention of BV recurrence will likely have to include several components to be successful, such as improved diagnostics, treatments, and prophylactic products (for example probiotics), but also

For peer review only - http://bmjopen.bmj.com/site/about/guidelines.xhtml

improved information, education, and counselling messages targeted to at-risk women and their partners. The results of this study can be used to inform future product development, and to fine-tune counselling messages in future trials.

For peer review only

For peer review only - http://bmjopen.bmj.com/site/about/guidelines.xhtml

Acknowledgments We thank the study participants, the Rinda Ubuzima team and other colleagues in Rwanda and the United Kingdom who contributed, the funders of this study, as well as the Trial Steering Committee for trial oversight. We thank Biose and Winclove for the donation of study products.

Author contributions JvdW obtained the research funding and wrote the study protocol and data collection documents. AN, EL, SA, and JvdW were members of the Trial Steering Committee. SA, MU, MMU, and JvdW collected the primary data. MU and MMU performed the FGDs and IDIs. MV and JvdW developed the analytical approach and performed the statistical analyses. MV and JvdW wrote the manuscript. All authors commented on and approved the final manuscript.

Funding This work was funded by the DFID/MRC/Wellcome Trust Joint Global Health Trials Scheme as a Development Project (grant reference MR/M017443/1; grant title: "Preparing for a clinical trial of interventions to maintain normal vaginal microbiota for preventing adverse reproductive health outcomes in Africa"). Vaginal probiotics for use in the trial were donated free of charge by Winclove Probiotics (Amsterdam, The Netherlands) and Biose (formerly Probionov; Aurillac, France). The findings and conclusions in this paper are those of the authors and do not necessarily represent the views of the authors' institutions or companies, or the funder. None of the authors were paid to write this article. The corresponding author had full access to the data and had final responsibility for the decision to submit for publication.

Competing interests AN is employed by Biose (owner of trial product GynLP) and EL by Winclove Probiotics BV (owner of trial product EF+). AN has financial and/or intellectual investments in competing products. The other authors report no competing interests.

Patient consent All participants provided written informed consent for study participation, and separate written informed consent for participation in FGDs/IDIs.

For peer review only

For peer review only - http://bmjopen.bmj.com/site/about/guidelines.xhtml

Ethics approval The study was sponsored by the University of Liverpool, approved by the Rwanda National Ethics Committee and the University of Liverpool Research Ethics Subcommittee for Physical Interventions, and registered on ClinicalTrials.gov (NCT02459665).

Data sharing statement The data supporting the findings of this publication are retained by the corresponding author (JvdW) and will not be made openly accessible due to privacy concerns. Fully anonymised data can be made available by written request to j.vandewijgert@liverpool.ac.uk after assurance that the intended data usage is compliant with relevant ethical approvals and privacy will be maintained.

For peer review only

For peer review only - http://bmjopen.bmj.com/site/about/guidelines.xhtml

Figure footnotes

Figure 1: Flowchart of the study

*Totals to 110 reasons among 102 women because there could be more than one reason per woman.
†Reasons: outside of metronidazole treatment window (n=5), enrolment target already met (n=4), has a mental disorder (n=1), did not complete screening procedures and was subsequently lost to follow=up (n=1), withdrew consent during the screening visit because she thought the reimbursement was too low (n=1).
‡Reasons: moved away from Kigali (n=2), lost interest because symptoms resolved (n=1), and was verbally harassed by partner and sister about study participation (n=1).

Acceptability assessments were made at enrolment and at the M2 visit. Adherence assessments were made using self-rated assessments, pictorial diary cards, and returned packaging at the D7, M1, and M2 visits (after which product use was ceased). The vaginal infection knowledge survey was held at recruitment sessions in the community and at the enrolment visit. Changes in sexual risk-taking and vaginal practices were assessed at each follow-up visits and compared to answers given during the enrol visit. All this themes were discussed during the eight FGDs and IDIs.

BV, bacterial vaginosis; D7, day 7 visit; FGD, focus group discussion; IDI, in-depth interview; M1/2/6, month 1/2/6 visit; RU, Rinda Ubuzima; TV, *Trichomonas vaginalis*.

For peer review only

References

1 van de Wijgert JHHM, Borgdorff H, Verhelst R, *et al.* The vaginal microbiota: what have we learned after a decade of molecular characterization? *PLOS ONE* 2014;**9**:e105998. doi:10.1371/journal.pone.0105998

2 van de Wijgert JHHM, Morrison CS, Cornelisse PGA, *et al.* Bacterial vaginosis and vaginal yeast, but not vaginal cleansing, increase HIV-1 acquisition in African women. *J Acquir Immune Defic Syndr 1999* 2008;**48**:203–10. doi:10.1097/QAI.0b013e3181743936

3 van de Wijgert JHHM. The vaginal microbiome and sexually transmitted infections are interlinked: consequences for treatment and prevention. *PLOS Med* 2017;**14**:e1002478. doi:10.1371/journal.pmed.1002478

4 Li J, McCormick J, Bocking A, *et al.* Importance of vaginal microbes in reproductive health. *Reprod Sci* 2012;**19**:235–42. doi:10.1177/1933719111418379

5 Nelson DB, Hanlon AL, Wu G, *et al.* First trimester levels of BV-associated bacteria and risk of miscarriage among women early in pregnancy. *Matern Child Health J* 2015;**19**:2682–7. doi:10.1007/s10995-015-1790-2

6 Hay P. Recurrent bacterial vaginosis. *Curr Opin Infect Dis* 2009;**22**:82–6. doi:10.1097/QCO.0b013e32832180c6

7 Verstraelen H, Verhelst R. Bacterial vaginosis: an update on diagnosis and treatment. *Expert Rev Anti Infect Ther* 2009;**7**:1109–24. doi:10.1586/eri.09.87

8 Torrone EA, Morrison CS, Chen P-L, *et al.* Prevalence of sexually transmitted infections and bacterial vaginosis among women in sub-Saharan Africa: An individual participant data meta-analysis of 18 HIV prevention studies. *PLOS Med* 2018;**15**:e1002511. doi:10.1371/journal.pmed.1002511

9 Bradshaw CS, Brotman RM. Making inroads into improving treatment of bacterial vaginosis – striving for long-term cure. *BMC Infect Dis* 2015;**15**:292. doi:10.1186/s12879-015-1027-4

10 Anukam KC, Osazuwa E, Osemene GI, *et al.* Clinical study comparing probiotic *Lactobacillus* GR-1 and RC-14 with metronidazole vaginal gel to treat symptomatic bacterial vaginosis. *Microbes Infect* 2006;**8**:2772–6. doi:10.1016/j.micinf.2006.08.008

11 Ling Z, Liu X, Chen W, *et al.* The restoration of the vaginal microbiota after treatment for bacterial vaginosis with metronidazole or probiotics. *Microb Ecol* 2013;**65**:773–80. doi:10.1007/s00248-012-0154-3

12 Ngugi BM, Hemmerling A, Bukusi EA, *et al.* Effects of bacterial vaginosis-associated bacteria and sexual intercourse on vaginal colonization with the probiotic *Lactobacillus crispatus* CTV-05. *Sex Transm Dis* 2011;**38**:1020–7. doi:10.1097/OLQ.0b013e3182267ac4

13 Bradshaw CS, Pirotta M, De Guingand D, *et al.* Efficacy of oral metronidazole with vaginal clindamycin or vaginal probiotic for bacterial vaginosis: randomised placebo-

For peer review only

For peer review only - http://bmjopen.bmj.com/site/about/guidelines.xhtml

controlled double-blind trial. *PLoS ONE* 2012;**7**:e34540. doi:10.1371/journal.pone.0034540

14 van der Straten A, Montgomery ET, Cheng H, *et al.* High acceptability of a vaginal ring intended as a microbicide delivery method for HIV prevention in African women. *AIDS Behav* 2012;**16**:1775–86. doi:10.1007/s10461-012-0215-0

15 Merkatz RB, Plagianos M, Hoskin E, *et al.* Acceptability of the nestorone®/ethinyl estradiol contraceptive vaginal ring: development of a model; implications for introduction. *Contraception* 2014;**90**:514–21. doi:10.1016/j.contraception.2014.05.015

16 van de Wijgert JHHM, Verwijs MC, Agaba SK, *et al*. Intermittent use of vaginal probiotics or oral metronidazole to prevent bacterial vaginosis recurrence: safety and preliminary efficacy by Nugent scoring and 16S rRNA gene sequencing. 2018. Conference talk presented at the Keystone Symposium "Role of the genital tract microbiome in sexual and reproductive health", Cape Town, South Africa.

17 Sobel JD, Ferris D, Schwebke J, *et al.* Suppressive antibacterial therapy with 0.75% metronidazole vaginal gel to prevent recurrent bacterial vaginosis. *Am J Obstet Gynecol* 2006;**194**:1283–9. doi:10.1016/j.ajog.2005.11.041

18 McClelland RS, Richardson BA, Hassan WM, *et al.* Improvement of vaginal health for Kenyan women at risk for acquisition of Human Immunodeficiency Virus type 1: results of a randomized trial. *J Infect Dis* 2008;**197**:1361–8. doi:10.1086/587490

19 Kestelyn E, Nuil JIV, Umulisa MM, *et al.* High acceptability of a contraceptive vaginal ring among women in Kigali, Rwanda. *PLOS ONE* 2018;**13**:e0199096. doi:10.1371/journal.pone.0199096

20 World Health Organization. Guidelines for the management of sexually transmitted infections. 2003.http://applications.emro.who.int/aiecf/web79.pdf (accessed 12 Mar 2019).

21 Binagwaho A, editor. *National guidelines for prevention and management of HIV, STIs & other blood borne infections*. Republic of Rwanda - Ministry of Health 2013. https://aidsfree.usaid.gov/sites/default/files/hts_policy_rwanda.pdf (accessed 12 Mar 2019).

22 Veldhuijzen N, Nyinawabega J, Umulisa M, *et al.* Preparing for microbicide trials in Rwanda: focus group discussions with Rwandan women and men. *Cult Health Sex* 2006;**8**:395–406. doi:10.1080/13691050600859302

23 Low N, Chersich MF, Schmidlin K, *et al.* Intravaginal practices, bacterial vaginosis, and HIV infection in women: individual participant data meta-analysis. *PLOS Med* 2011;**8**:e1000416. doi:10.1371/journal.pmed.1000416

24 Dausset C, Patrier S, Gajer P, *et al.* Comparative phase I randomized open-label pilot clinical trial of Gynophilus® (Lcr regenerans®) immediate release capsules versus slow release muco-adhesive tablets. *Eur J Clin Microbiol Infect Dis* 2018;**37**:1869–80. doi:10.1007/s10096-018-3321-8

For peer review only

For peer review only - http://bmjopen.bmj.com/site/about/guidelines.xhtml

25 Safren SA, W. Otto M, Worth JL, *et al.* Two strategies to increase adherence to HIV antiretroviral medication: Life-Steps and medication monitoring. *Behav Res Ther* 2001;**39**:1151–62. doi:10.1016/S0005-7967(00)00091-7

26 Glick P, Sahn DE. Changes in HIV/AIDS knowledge and testing behavior in Africa: how much and for whom? *J Popul Econ* 2007;**20**:383–422. doi:10.1007/s00148-006-0085-8

27 Peltzer K, Matseke G, Mzolo T, *et al.* Determinants of knowledge of HIV status in South Africa: results from a population-based HIV survey. *BMC Public Health* 2009;**9**:174. doi:10.1186/1471-2458-9-174

28 Fay H, Baral SD, Trapence G, *et al.* Stigma, health care access, and HIV knowledge among men who have sex with men in Malawi, Namibia, and Botswana. *AIDS Behav* 2011;**15**:1088–97. doi:10.1007/s10461-010-9861-2

For peer review only

For peer review only - http://bmjopen.bmj.com/site/about/guidelines.xhtml

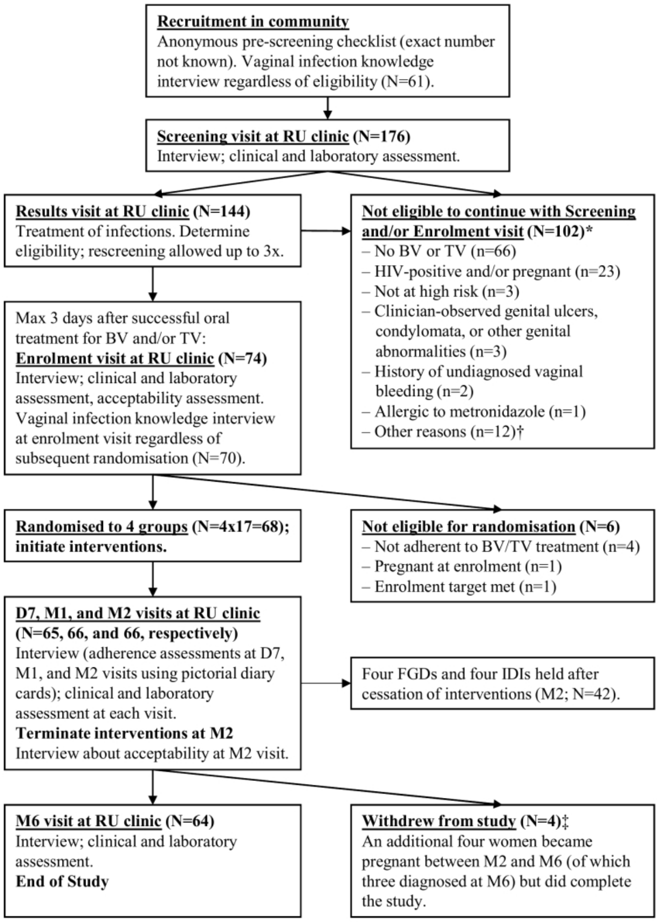

Figure 1: Flowchart of the study
*Totals to 110 reasons among 102 women because there could be more than one reason per woman.
†Reasons: outside of metronidazole treatment window (n=5), enrolment target already met (n=4), has a mental disorder (n=1), did not complete screening procedures and was subsequently lost to follow=up (n=1), withdrew consent during the screening visit because she thought the reimbursement was too low (n=1).
‡Reasons: moved away from Kigali (n=2), lost interest because symptoms resolved (n=1), and was verbally harassed by partner and sister about study participation (n=1).

Acceptability assessments were made at enrolment and at the M2 visit. Adherence assessments were made using self-rated assessments, pictorial diary cards, and returned packaging at the D7, M1, and M2 visits (after which product use was ceased). The vaginal infection knowledge survey was held at recruitment sessions in the community and at the enrolment visit. Changes in sexual risk-taking and vaginal practices were assessed at each follow-up visits and compared to answers given during the enrol visit. All this themes

were discussed during the eight FGDs and IDIs.

BV, bacterial vaginosis; D7, day 7 visit; FGD, focus group discussion; IDI, in-depth interview; M1/2/6, month 1/2/6 visit; RU, Rinda Ubuzima; TV, *Trichomonas vaginalis*.

215x279mm (300 x 300 DPI)

Supplementary Figure 1: Pictorial diary card

Date/Month	Descriptions	Monday	Tuesday	Wednesday	Thursday	Friday	Saturday	Sunday
Indicate each time you used study product								
	Used study product							
Indicate each sex act								
	Sex with condom							
	Sex without condom							
Indicate each time you washed/inserted something inside the vagina other than study product								
By washing inside, we mean inserting an entire finger inside the vaginal canal								
	Washed inside vagina with water only							
	Washed inside vagina with soap and water							
	Inserted something else (herbs, powders, etc.)							
Indicate each day of menstrual bleeding								
	Had menstrual bleeding							

The picture provided is the English translation of the pictorial card; participants received a version in Kinyarwanda.

Supplementary Table 1: Baseline characteristics of enrolled population

	Controls (n=17)	Metronidazole (n=17)	EF+ (n=17)	GynLP (n=17)
Median age (IQR)	29 (24–36)	30 (27–34)	33 (28–35)	30 (27–35)
Marital status n (%)				
- Never married	16 (94.1)	11 (64.7)	10 (58.8)	13 (76.5)
- Married	1 (5.9)	1 (5.9)	2 (11.8)	1 (5.9)
- Divorced	0	5 (29.4)	4 (23.5)	3 (17.6)
- Widowed	0	0	1 (5.9)	0
Education level n (%)				
- No schooling	5 (29.4)	3 (17.6)	3 (17.6)	3 (17.7)
- Primary school not completed	7 (41.2)	7 (41.2)	13 (76.5)	4 (23.5)
- Primary school completed	4 (23.5)	5 (29.4)	1 (5.9)	7 (41.2)
- At least some secondary school	1 (5.9)	2 (11.8)	0	3 (17.7)
Median number of sex partners last month (IQR)	5 (3–20)	5 (2–10)	3 (2–15)	3 (2–20)
Exchanged sex for money/goods past month n (%)	17 (100)	14 (82.4)	15 (88.2)	17 (100)
At least one laboratory-confirmed STI* n (%)	8 (47.1)	8 (47.1)	4 (23.5)	9 (52.9)
Median weekly frequency of washing body (IQR)	7 (7–7)	7 (7–7)	7 (7–7)	7 (4–7)
Ever washing the genitalia n (%)				
- Yes, outside only	12 (70.7)	14 (82.4)	15 (88.3)	14 (82.3)
- Yes, both inside and outside	5 (29.4)	3 (17.6)	2 (11.7)	3 (17.7)
- Yes, inside only	0	0	0	0
If reports washing inside, median weekly frequency (IQR)	14 (7–16)	14 (14–14)	11 (7–14)	7 (3–12)

*Chlamydia, gonorrhoea, and/or syphilis.
EF+, Ecologic Femi+; Enr, enrolment visit; GynLP, Gynophilus LP; IQR, inter-quartile range; M2, Month 2 visit; STI, sexually transmitted disease.

For peer review only

Supplementary Table 2: Acceptability of interventions

**Acceptability of study products at Enr**	**Controls (n=17)**	**Metronidazole (n=17)**	**EF+ (n=17)**	**GynLP (n=17)**
Nurse reports having explained intervention to participant in detail n (%)	17 (100)	17 (100)	17 (100)	17 (100)
Nurse reports participant asked questions n (%)*				
- Yes, a few	6 (35.3)	2 (11.8)	11 (64.7)	11 (64.7)
- Yes, many	0	0	0	2 (11.8)
First dose applied† under supervision n (%)	NA	17 (100)	17 (100)	17 (100)
Median number of attempts participant made until successful application (IQR)	NA	NA	1 (1–1)	1 (1–1)
Participant seemed comfortable with the insertion after these attempts, according to study nurse n (%)	NA	NA		
- Yes, very			17 (100)	16 (94.1)
- Yes, somewhat			0	1 (5.9)
**Acceptability of study products at M2**				
Self-reported usual time of insertion n (%)				
- Before going to sleep	NA	NA	17 (100)	15 (100)‡
- After bathing in the morning			0	0
Level of comfort with vaginal insertion after 2 months of use, self-reported n (%)	NA	NA		
- Very comfortable			17 (100)	15 (100)‡
- Somewhat comfortable			0	0
Reported insertion becoming easier over time n (%)	NA	NA	17 (100)	15 (100)‡
Reported manner of insertion§ n (%)				
- While lying down	NA	NA	17 (100)	14 (93.3)‡
- While squatting			1 (5.9)	1 (6.7)
**Acceptability of penile hygiene intervention at M2**				
Reports having told main sex partner to regularly clean the penis, including underneath the foreskin n (%)¶				
- Yes	3 (17.7)	3 (18.8)	3 (17.6)	3 (18.8)
- No, because he is circumcised	10 (58.8)	9 (56.2)	6 (35.3)	5 (31.3)
- No, other reason	1 (5.9)	0	1 (5.9)	1 (6.3)
If yes, response by the main partner n (%)‖				
- He said that he would do so in the future	2 (66.7)	1 (33.3)	1 (33.3)	1 (33.3)
- He said that he already does this	1 (33.3)	1 (33.3)	0	1 (33.3)
- He said that he is not interested	0	1 (33.3)	2 (66.7)	1 (33.3)

*One missing value.
†Whether oral insertion (oral metronidazole group) or vaginal insertion (Ecologic Femi+ and Gynophilus LP groups).
‡N=15 due to participants withdrawing informed consent.
§Multiple answers possible; hence totals can be more than 100%.
¶Women with no main sex partner not included.
‖N=3 in all four groups.
EF+, Ecologic Femi+; Enr, enrolment visit; GynLP, Gynophilus LP; IQR, inter-quartile range; M2, Month 2 visit; NA, Not applicable.

For peer review only

Supplementary Figure 1: Pictorial diary card

Date/Month	Descriptions	Monday	Tuesday	Wednesday	Thursday	Friday	Saturday	Sunday
Indicate each time you used study product								
	Used study product							
Indicate each sex act								
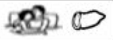	Sex with condom							
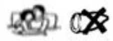	Sex without condom							
Indicate each time you washed/inserted something inside the vagina other than study product								
By washing inside, we mean inserting an entire finger inside the vaginal canal								
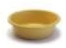	Washed inside vagina with water only							
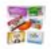	Washed inside vagina with soap and water							
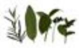	Inserted something else (herbs, powders, etc.)							
Indicate each day of menstrual bleeding								
	Had menstrual bleeding							

The picture provided is the English translation of the pictorial card; participants received a version in Kinyarwanda.

For peer review only

Supplementary Table 1: Baseline characteristics of enrolled population

	**Controls (n=17)**	**Metronidazole (n=17)**	**EF+ (n=17)**	**GynLP (n=17)**
Median age (IQR)	29 (24–36)	30 (27–34)	33 (28–35)	30 (27–35)
Marital status n (%)				
- Never married	16 (94.1)	11 (64.7)	10 (58.8)	13 (76.5)
- Married	1 (5.9)	1 (5.9)	2 (11.8)	1 (5.9)
- Divorced	0	5 (29.4)	4 (23.5)	3 (17.6)
- Widowed	0	0	1 (5.9)	0
Education level n (%)				
- No schooling	5 (29.4)	3 (17.6)	3 (17.6)	3 (17.7)
- Primary school not completed	7 (41.2)	7 (41.2)	13 (76.5)	4 (23.5)
- Primary school completed	4 (23.5)	5 (29.4)	1 (5.9)	7 (41.2)
- At least some secondary school	1 (5.9)	2 (11.8)	0	3 (17.7)
Median number of sex partners last month (IQR)	5 (3–20)	5 (2–10)	3 (2–15)	3 (2–20)
Exchanged sex for money/goods past month n (%)	17 (100)	14 (82.4)	15 (88.2)	17 (100)
At least one laboratory-confirmed STI* n (%)	8 (47.1)	8 (47.1)	4 (23.5)	9 (52.9)
Median weekly frequency of washing body (IQR)	7 (7–7)	7 (7–7)	7 (7–7)	7 (4–7)
Ever washing the genitalia n (%)				
- Yes, outside only	12 (70.7)	14 (82.4)	15 (88.3)	14 (82.3)
- Yes, both inside and outside	5 (29.4)	3 (17.6)	2 (11.7)	3 (17.7)
- Yes, inside only	0	0	0	0
If reports washing inside, median weekly frequency (IQR)	14 (7–16)	14 (14–14)	11 (7–14)	7 (3–12)

*Chlamydia, gonorrhoea, and/or syphilis.
EF+, Ecologic Femi+; Enr, enrolment visit; GynLP, Gynophilus LP; IQR, inter-quartile range; M2, Month 2 visit; STI, sexually transmitted disease.

For peer review only

Supplementary Table 2: Acceptability of interventions

Acceptability of study products at Enr	Controls (n=17)	Metronidazole (n=17)	EF+ (n=17)	GynLP (n=17)
Nurse reports having explained intervention to participant in detail n (%)	17 (100)	17 (100)	17 (100)	17 (100)
Nurse reports participant asked questions n (%)*<br>- Yes, a few<br>- Yes, many	 6 (35.3)<br>0	 2 (11.8)<br>0	 11 (64.7)<br>0	 11 (64.7)<br>2 (11.8)
First dose applied† under supervision n (%)	NA	17 (100)	17 (100)	17 (100)
Median number of attempts participant made until successful application (IQR)	NA	NA	1 (1–1)	1 (1–1)
Participant seemed comfortable with the insertion after these attempts, according to study nurse n (%)<br>- Yes, very<br>- Yes, somewhat	NA	NA	 <br>17 (100)<br>0	 <br>16 (94.1)<br>1 (5.9)
**Acceptability of study products at M2**				
Self-reported usual time of insertion n (%)<br>- Before going to sleep<br>- After bathing in the morning	NA	NA	 17 (100)<br>0	 15 (100)‡<br>0
Level of comfort with vaginal insertion after 2 months of use, self-reported n (%)<br>- Very comfortable<br>- Somewhat comfortable	NA	NA	 <br>17 (100)<br>0	 <br>15 (100)‡<br>0
Reported insertion becoming easier over time n (%)	NA	NA	17 (100)	15 (100)‡
Reported manner of insertion§ n (%)<br>- While lying down<br>- While squatting	NA	NA	 17 (100)<br>1 (5.9)	 14 (93.3)‡<br>1 (6.7)
**Acceptability of penile hygiene intervention at M2**				
Reports having told main sex partner to regularly clean the penis, including underneath the foreskin n (%)¶<br>- Yes<br>- No, because he is circumcised<br>- No, other reason	 <br>3 (17.7)<br>10 (58.8)<br>1 (5.9)	 <br>3 (18.8)<br>9 (56.2)<br>0	 <br>3 (17.6)<br>6 (35.3)<br>1 (5.9)	 <br>3 (18.8)<br>5 (31.3)<br>1 (6.3)
If yes, response by the main partner n (%)‖<br>- He said that he would do so in the future<br>- He said that he already does this<br>- He said that he is not interested	 2 (66.7)<br>1 (33.3)<br>0	 1 (33.3)<br>1 (33.3)<br>1 (33.3)	 1 (33.3)<br>0<br>2 (66.7)	 1 (33.3)<br>1 (33.3)<br>1 (33.3)

*One missing value.
†Whether oral insertion (oral metronidazole group) or vaginal insertion (Ecologic Femi+ and Gynophilus LP groups).
‡N=15 due to participants withdrawing informed consent.
§Multiple answers possible; hence totals can be more than 100%.
¶Women with no main sex partner not included.
‖N=3 in all four groups.
EF+, Ecologic Femi+; Enr, enrolment visit; GynLP, Gynophilus LP; IQR, inter-quartile range; M2, Month 2 visit; NA, Not applicable.

For peer review only

STROBE Statement—Checklist of items that should be included in reports of ***cohort studies***

	Item No	Recommendation	Reported on page #
**Title and abstract**	1	(*a*) Indicate the study's design with a commonly used term in the title or the abstract	1
		(*b*) Provide in the abstract an informative and balanced summary of what was done and what was found	2
**Introduction**			
Background/rationale	2	Explain the scientific background and rationale for the investigation being reported	4
Objectives	3	State specific objectives, including any prespecified hypotheses	4
**Methods**			
Study design	4	Present key elements of study design early in the paper	4
Setting	5	Describe the setting, locations, and relevant dates, including periods of recruitment, exposure, follow-up, and data collection	4
Participants	6	(*a*) Give the eligibility criteria, and the sources and methods of selection of participants. Describe methods of follow-up	4,5
		(*b*) For matched studies, give matching criteria and number of exposed and unexposed	NA
Variables	7	Clearly define all outcomes, exposures, predictors, potential confounders, and effect modifiers. Give diagnostic criteria, if applicable	6
Data sources/ measurement	8*	For each variable of interest, give sources of data and details of methods of assessment (measurement). Describe comparability of assessment methods if there is more than one group	6
Bias	9	Describe any efforts to address potential sources of bias	6
Study size	10	Explain how the study size was arrived at	NA (Primary outcomes paper)
Quantitative variables	11	Explain how quantitative variables were handled in the analyses. If applicable, describe which groupings were chosen and why	6
Statistical methods	12	(*a*) Describe all statistical methods, including those used to control for confounding	6,7
		(*b*) Describe any methods used to examine subgroups and interactions	6,7
		(*c*) Explain how missing data were addressed	NA (indicated in tables if missing)
		(*d*) If applicable, explain how loss to follow-up was addressed	NA
		(*e*) Describe any sensitivity analyses	NA
**Results**			
Participants	13*	(a) Report numbers of individuals at each stage of study—eg numbers potentially eligible, examined for eligibility, confirmed eligible, included in the study, completing follow-up, and analysed	Page 8, Figure 1
		(b) Give reasons for non-participation at each stage	8, Figure 1
		(c) Consider use of a flow diagram	Figure 1
Descriptive data	14*	(a) Give characteristics of study participants (eg demographic, clinical, social) and information on exposures and potential confounders	8
		(b) Indicate number of participants with missing data for each variable of	8 (missing

For peer review only - http://bmjopen.bmj.com/site/about/guidelines.xhtml

		interest	data in footnotes in table, if applicable)
		(c) Summarise follow-up time (eg, average and total amount)	8
Outcome data	15*	Report numbers of outcome events or summary measures over time	9
Main results	16	(*a*) Give unadjusted estimates and, if applicable, confounder-adjusted estimates and their precision (eg, 95% confidence interval). Make clear which confounders were adjusted for and why they were included	9
		(*b*) Report category boundaries when continuous variables were categorized	Table 1
		(*c*) If relevant, consider translating estimates of relative risk into absolute risk for a meaningful time period	NA
Other analyses	17	Report other analyses done—eg analyses of subgroups and interactions, and sensitivity analyses	Page 12 and beyond
**Discussion**			
Key results	18	Summarise key results with reference to study objectives	Page 18
Limitations	19	Discuss limitations of the study, taking into account sources of potential bias or imprecision. Discuss both direction and magnitude of any potential bias	Page 19
Interpretation	20	Give a cautious overall interpretation of results considering objectives, limitations, multiplicity of analyses, results from similar studies, and other relevant evidence	Page 18, 19
Generalisability	21	Discuss the generalisability (external validity) of the study results	Page 18, 19
**Other information**			
Funding	22	Give the source of funding and the role of the funders for the present study and, if applicable, for the original study on which the present article is based	Page 21

*Give information separately for exposed and unexposed groups.

Note: An Explanation and Elaboration article discusses each checklist item and gives methodological background and published examples of transparent reporting. The STROBE checklist is best used in conjunction with this article (freely available on the Web sites of PLoS Medicine at http://www.plosmedicine.org/, Annals of Internal Medicine at http://www.annals.org/, and Epidemiology at http://www.epidem.com/). Information on the STROBE Initiative is available at http://www.strobe-statement.org.

For peer review only

Vaginal Probiotic Adherence and Acceptability in High-Risk Rwandan Women Participating in a Pilot Randomised Controlled Trial: A Mixed-Methods Approach

Authors: Marijn C. Verwijs (MD)[1], Stephen K. Agaba (MD)[2], Marie-Michele Umulisa (MA)[2], Mireille Uwineza (BA)[2], Adrien Nivoliez (PhD)[3], Elke Lievens (PhD)[4], Janneke H.H.M. van de Wijgert (MD PhD MPH)[1,5].

Author affiliations

[1]Institute of Infection and Global Health, University of Liverpool, Liverpool, United Kingdom.

[2]Rinda Ubuzima, Kigali, Rwanda.

[3]Biose, Aurillac, France.

[4]Winclove Probiotics, Amsterdam, The Netherlands.

[5]Julius Center for Health Sciences and Primary Care, University Medical Center Utrecht, Utrecht University, Utrecht, The Netherlands.

Correspondence to

Professor Janneke van de Wijgert, MD PhD MPH

Department of Clinical Infection, Microbiology and Immunology

Institute of Infection and Global Health, University of Liverpool

Ronald Ross Building, 8 West Derby Street, Liverpool L69 7BE, United Kingdom

Email: j.vandewijgert@liverpool.ac.uk

ORCID: 0000-0003-2728-4560

Word counts:

Word count abstract: 300 words (max. 300 words).

Word count main text: 3,~~104~~ 479 words (max. 4000 words).

Number of tables and figures: 5 (max. 5).

For peer review only - http://bmjopen.bmj.com/site/about/guidelines.xhtml

For peer review only

Number of supplementary tables and figures: ~~2~~<u>3</u>.

For peer review only

For peer review only - http://bmjopen.bmj.com/site/about/guidelines.xhtml

ABSTRACT

Objectives ~~The recurrence of b~~Bacterial vaginosis (BV) recurrence is common. We evaluated the adherence and acceptability of intermittent use of two vaginal probiotics and one antibiotic to prevent recurrence.

Design Repeated adherence and acceptability assessments using mixed methods within a pilot randomised controlled trial.

Setting Research clinic in Kigali, Rwanda.

Participants High-risk Rwandan women (n=68) with BV and/or trichomoniasis.

Interventions Women were randomised to four groups (n=17 each) after completing metronidazole treatment: behavioural counselling only, or behavioural counselling plus two-month intermittent use of oral metronidazole, Ecologic Femi+ (EF+) vaginal capsule, or Gynophilus LP (GynLP) vaginal tablet~~ for two months~~.

Outcome measures Adherence and acceptability data from ~~the~~ randomised women were collected in structured face-to-face interviews, semi-structured focus group discussions and in-depth interviews, daily diaries, and counting of used/unused study products. Randomised ~~W~~women and women attending recruitment sessions (n=131) were surveyed about ~~their~~ vaginal infection knowledge.

Results Most ~~randomised~~ women (93%) were sex workers. At baseline, they were unfamiliar with BV, and had never used probiotics. All ~~vaginal~~ probiotic users reported that insertion became easier over time. Triangulated adherence data showed that 100% of EF+ users and 88.2% of GynLP users used ≥80% of required doses. Younger age, asking many questions at enrolment, having menses, and reporting urogenital symptoms showed non-significant trends towards a lower perfect adherence likelihood. Qualitative data suggested that women believed that the probiotics reduced BV recurrence, but that partners were sometimes unsupportive of study participation. Self-reported vaginal washing practices decreased during follow-up, but sexual risk behaviours did not. Most women (80%) with an uncircumcised steady partner discussed penile hygiene with him, but many women found this difficult, especially with male clients.

For peer review only

For peer review only - http://bmjopen.bmj.com/site/about/guidelines.xhtml

Conclusions High-risk women require education about vaginal infections. Vaginal probiotic acceptability and adherence were high in this cohort. Our results can be used to inform future product development and to fine-tune counselling messages in prevention programs.

Trial registration ClinicalTrials.gov (NCT02459665).

Keywords (5) bacterial vaginosis, vaginal probiotic, adherence, acceptability, Africa.

ARTICLE SUMMARY

Strengths and limitations of this study

- We conducted this research in the context of a ~~tightly controlled and conducted~~ pilot randomised controlled trial, and statistical power was therefore limited. ~~However, our statistical power was limited.~~
- We triangulated different sources of adherence data to maximise accuracy, and used a mixed-methods approach to evaluate acceptability. ~~However,~~
- We could not directly compare experiences with, and opinions about, the two different vaginal probiotics because each woman used only one product and qualitative data depth was suboptimal.
- ~~social~~ Social desirability bias may have affected some of the results.
- ~~We enrolled women at high risk of HIV in Kigali, Rwanda, the majority of whom were female sex workers.~~ The results of this study may not be generalizable to ~~other groups of~~ women at lower risk of HIV/STIs.

For peer review only

For peer review only - http://bmjopen.bmj.com/site/about/guidelines.xhtml

INTRODUCTION

Bacterial vaginosis (BV) is a vaginal condition in which fastidious anaerobes such as *Gardnerella vaginalis* increase while beneficial, lactic acid-producing lactobacilli decrease.[1] Often asymptomatic, it is associated with increased risks of sexually transmitted infections (STIs) and HIV transmission, pelvic inflammatory disease, and adverse pregnancy outcomes.[2–5] Although BV is treatable with antibiotics, the risk of recurrence is high.[6,7] ~~BV~~ The prevalence of BV varies among regions and ethnic groups but is highest in sub-Saharan Africa, where it is estimated at 30-50%.[8]

Vaginally-administered probiotics containing lactobacilli are considered a promising new strategy to restore a lactobacilli-dominated vaginal microbiota during and/or after antibiotic treatment, or to prevent BV.[9] While some probiotics have been available on the market for several years, clinical trials to support beneficial effects have only recently been initiated for most products.[10–13] Acceptability is an important component of these trials, to maximise future uptake and adherence of vaginal probiotics should they be proven efficacious. The acceptability of a novel vaginal product depends on factors such as the characteristics of the population studied, characteristics of and experiences with the product, types of sexual relationships and partner support, and community perceptions.[14,15]

For peer review only

We conducted a clinical trial of intermittent use of two vaginal probiotics and oral metronidazole to prevent BV recurrence in Rwandan women who had been treated for BV and/or *Trichomonas vaginalis* (TV). We used qualitative and quantitative research methods to assess adherence and acceptability with vaginal probiotic use. We triangulated various sources of adherence data to obtain adherence estimates per woman for each period of intermittent product use in between study visits, and determined correlates of adherence.

METHODS

The pilot clinical trial took place from June 2015 to February 2016 at the Rinda Ubuzima research clinic in Kigali, Rwanda. Women who had been successfully treated for BV/TV with a seven-day

For peer review only - http://bmjopen.bmj.com/site/about/guidelines.xhtml

course of oral metronidazole (Tricozole, Laboratory & Allied Ltd, Nairobi, Kenya) were randomised to four intervention groups (n=17 each) to prevent BV recurrence: bBehavioural counselling only (controls), or behavioural counselling plus intermittent use of two different vaginal probiotics or oral metronidazole for two months. Women were seen at screening, enrolment (product use initiation, if applicable), Day 7, Month 1, Month 2 (product use cessation, if applicable), and Month 6. Product efficacies were not known during the trial, and preliminary efficacy results are reported elsewhere.[16] The behavioural counselling focussed on safer sex practices, cessation of vaginal practices, and increasing male penile hygiene to prevent BV.

For peer review only

Study population

Women aged 18-45 at risk of HIV/STIs (defined as having had more than one sex partner and/or having been treated for an STI and/or BV in the last 12 months) were eligible for enrolment if they were confirmed HIV-negative, non-pregnant, diagnosed with BV and/or TV, and cured after seven-day oral metronidazole treatment. Other clinical exclusion criteria were applied but were rare.[16] Women were recruited by study staff with the assistance of Community Mobilisers who had strong ties with local high-risk women (particularly sex workers).

Study products and dosing

Ecologic Femi+ (EF+; Winclove Probiotics, Amsterdam, Netherlands) is a vaginal capsule containing lyophilised lactic acid-producing bacteria. EF+ was used once per day for five days followed by thrice weekly, for two months. Gynophilus LP (GynLP; Biose, Aurillac, France) is a tablet containing the *Lactobacillus rhamnosus* Lcr35 strain. The tablet disintegrates in the vagina and forms a gel that slowly releases the probiotic bacteria. GynLP was used once every four days for two months. The first dose was inserted at the clinic under direct observation of a clinician, and remaining doses were self-administered at home. Women were asked not to cleanse or insert other products into the vagina after probiotic insertion to allow the probiotics to dissolve. They were also told that they were allowed to cease probiotic use during menses, but were encouraged to continue. Intermittent metronidazole use was chosen as a positive control intervention because studies conducted in the U.S. and Kenya have

For peer review only - http://bmjopen.bmj.com/site/about/guidelines.xhtml

shown a 30-40% reduction in BV recurrence.[17,18] Metronidazole users took 500 mg generic oral metronidazole (Laboratory & Allied ltd, Nairobi, Kenya) twice weekly for two months. Participants and clinicians were not blinded.

~~Adherence and~~ Aacceptability, adherence, behavioural, and vaginal infection knowledge assessments

Acceptability was assessed at the enrolment visit prior to product use initiation and at the Month 2 visit after the full two months of use. Adherence was assessed during the intervention period, at the Day 7, Month 1, and Month 2 visits. Sexual and other behaviours were assessed at all study visits. Participants were interviewed face-to-face in Kinyarwanda by a trained study nurse using structured questionnaires with multiple-choice questions, questions requiring a number or date, and an adherence self-rating scale (from 0-10). In between visits, participants used pictorial diary cards (online supplementary material figure 1) to record daily episodes of product use, vaginal sex, condom use, and vaginal practices. Those using study products returned the product packaging and unused products (if applicable) to their clinic visits, where they were counted by study staff. ~~At each visit, trained study nurses interviewed participants in Kinyarwanda using a structured questionnaire (including a self-rating adherence scale from 0-10), and reviewed daily diaries and returned packaging.~~ Any discrepancies between data sources were discussed with participants, and consensus, triangulated assessments of adherence were recorded on the questionnaires. Additionally, 131 women were interviewed about their knowledge of vaginal infections (such as BV and STIs) using a structured questionnaire during recruitment sessions (n=61; regardless of eligibility) and at enrolment visits (n=70; this included the 68 randomised women, and two women who attended enrolment visits but turned out to be ineligible). Women were interviewed before being counselled at study visits or before receiving information at recruitment sessions. This questionnaire contained multiple-choice and open-ended questions. Responses to the open-ended questions were categorised and discussed by two different researchers until consensus about the answer categories was reached.

For peer review only - http://bmjopen.bmj.com/site/about/guidelines.xhtml

Four semi-structured focus group discussions (FGDs) with 7-11 participants per group (total n=38), and four semi-structured individual in-depth interviews (IDIs) were held with enrolled participants, about their experiences with and opinions of the products, ~~. The acceptability of and adherence to the products were also discussed, as well as changes in~~ sexual behaviour, and vaginal practices. Women randomised to the behavioural counselling only group were not included in the FGDs and IDIs. All had completed their product use period. The interviews were unlinked anonymous, and women used pseudonyms to enable them to talk freely despite the fact that the discussions and interviews were taped. All interviews took place between November 2015 and March 2016, were held in Kinyarwanda, recorded on tape, transcribed verbatim, and translated into English.

Data analysis

Q~~uantitative~~ uestionnaire data were analysed using Stata 13 (StataCorp, College Station, TX, USA). The proportion of women with ≥80%/≥90%/100% adherence in the probiotic groups were compared by Fisher's exact tests. Changes in self-reported vaginal practices and sexual behaviours over time were tested using McNemar's test for binary outcomes, and Wilcoxon's signed-rank test for continuous~~,~~ outcomes. To study associations of participant characteristics with triangulated adherence, we used bivariable mixed effects models, with perfect adherence (defined as having used all doses as instructed) per interval between study visits during the intervention period as the outcome, participant identification numbers as the random effect, and one participant characteristic at the time as the fixed effect. We could not determine correlates of acceptability due to limited variation in the acceptability data (reported acceptability was high throughout the trial).

The ~~eight~~ FGD~~s~~ and IDI transcripts were read and discussed by three researchers (MV, MU, and JvdW). The Chief Investigator (JvdW) decided that data saturation had been met when the fourth FGD and the fourth IDI transcript had become available in March 2016. The transcripts were then coded using NVivo 10.0 (QSR International, Melbourne, Australia) by one single researcher (MV). The discussions and interviews were semi-structured, with themes and associated codes prepared a priori, as well as new elements that emerged from the data. The codes were derived from an

For peer review only

For peer review only - http://bmjopen.bmj.com/site/about/guidelines.xhtml

acceptability framework that has been used in studies of vaginal products for contraception or HIV prevention.[14,15,19] Components of the framework include study population characteristics, product attributes, sexual encounter and relational attributes, and the contextual environment (e.g. community perceptions of product use).

Ethical statement

All participants provided written consent for study participation, and separate consent for participation in FGDs/IDIs. All non-married participants aged 18-20 also required parental/guardian consent per Rwandan law at the time of the study. The participants received 3 GBP per visit (in local currency) as a reimbursement for time and transport costs. Care was taken to protect participant privacy and confidentiality. The study was sponsored by the University of Liverpool, approved by the Rwanda National Ethics Committee and the University of Liverpool Research Ethics Subcommittee for Physical Interventions, and registered on ClinicalTrials.gov (NCT02459665).

Participant and public involvement

A subset of the enrolled participants were invited to comment on study design and experiences with the interventions during the FGDs/IDIs. Participants were not invited to develop outcomes, interpret the results, or to contribute to the writing or editing of this document for readability or accuracy. The preliminary results of this study were discussed with 32 stakeholders during a workshop held at the Ministry of Health in Kigali, Rwanda, in December 2017. These stakeholders included representatives of the Ministry of Health, the National University of Rwanda, the National Ethics Committee, local hospitals and clinics, and local non-governmental and women's organisations.

RESULTS

Baseline characteristics

We screened 176 women: bacterial STI prevalence was 31.3% and BV prevalence by Gram stain Nugent scoring was 47.9%. All 68 randomised women were at risk of STI/HIV transmission, with 93.1% reporting having exchanged sex for money and/or goods in the previous month (figure 1,

For peer review only

For peer review only - http://bmjopen.bmj.com/site/about/guidelines.xhtml

online supplementary material table 1). We collected 29.93 person-years of data. Four women withdrew their informed consent during the study (for reasons unrelated to study product acceptability). None were lost to follow-up.

Adherence

Triangulated adherence was high: 100% of EF+ users and 88.2% of GynLP users used ≥80% of required doses (Fisher's exact p=0.103; table 1), and these percentages were 88.2% and 68.8% for ≥90% (p=0.225), and 58.8% and 50% for 100% of required doses (p=0.732), respectively. In comparison, these percentages were 88.2%, 82.4%, and 70.6%, respectively, for oral metronidazole users. Reported reasons of non-adherence to vaginal probiotics during face-to-face interviews were 'simply forgetting' (n=9), experiencing side-effects (n=2), menses (n=2), and being away from home and having left products at home (n=1). Additional reasons for missing doses mentioned during FGDs/IDIs were being drunk (n=2) and being confused about the dosing schedule (n=2). Only one woman in the metronidazole arm reported missing doses due to experiencing side-effects. Most women in FGDs reported using all doses as instructed and finding it easy to adhere, and thought that the diary cards served as a useful reminder to use the products.

For peer review only

For peer review only - http://bmjopen.bmj.com/site/about/guidelines.xhtml

Table 1: Adherence to study interventions

**Adherence to study products**	**Metronidazole (n=17)**	**EF+ (n=17)**	**GynLP (n=16)**
Adherence Enr–D7, median % (IQR)	100 (100–100)	100 (100–100)	100 (100–100)
Adherence D7–M1, median % (IQR)	100 (100–100)	100 (100–100)	100 (91.7–100)
Adherence M1–M2, median % (IQR)	100 (100–100)	100 (100–100)	100 (92.3–100)
Overall adherence Enr–M2, median % (IQR)	100 (96.3–100)	100 (100–100)	98.3 (89.3–100)
Overall adherence Enr–M2 n (%)			
- Perfect*	12 (70.6)	10 (58.8)	8 (50.0)
- Adherence ≥90%	14 (82.4)	15 (88.2)	11 (68.8)
- Adherence ≥80%	15 (88.2)	17 (100)	13 (81.3)
Number of times menses Enr–M2 n (%)†			
- Never	7 (41.2)	4 (23.5)	2 (12.5)
- Once	6 (35.3)	5 (29.4)	4 (25.0)
- Twice	4 (23.5)	8 (47.1)	10 (62.5)
Did not use product during menses at least once n (%)			
- Yes	4 (23.5)	3 (17.6)	5 (31.3)
- NA (never had menses)	7 (41.2)	4 (23.5)	2 (12.5)
**Self-reported reasons for non-adherence‡**	**Metronidazole**	**EF+**	**GynLP**
D7: Self-reported reasons why not able to use all doses as instructed n (%)§			
- Simply forgot	0	2 (11.8)	0
- Product had side effects	0	0	1 (6.7)¶
M1: Self-reported reasons why not able to use all doses as instructed n (%)§			
- Simply forgot	1 (6.3)	1 (5.9)	1 (6.3)
- Product had side effects	1 (6.3)‖	0	1 (6.3)‡‡
- Did not like product for another reason	1 (6.3)‖	0	0
- Other	1 (6.3)**	1 (5.9)††	2 (12.5)§§
M2: Self-reported reasons why not able to use all doses as instructed n (%)§			
- Simply forgot	1 (6.3)	2 (11.8)	3 (18.8)
- Travelled and forgot to take product	1 (6.3)	0	1 (6.25)
- Other	0	1 (5.9)¶¶	1 (6.3)‖‖
D7: Participant thinks she used product correctly most of the time n (%)	17 (100)	16 (94.1)	14 (93.3)
M1: Participant thinks she used product correctly most of the time n (%)	13 (86.7)	17 (100)	11 (68.8)
M2: Participant thinks she used product correctly most of the time n (%)	15 (93.7)	16 (94.1)	14 (87.5)

*Defined as 100% of the prescribed doses used at the prescribed times after nurse review of the participant's diary card and returned used packaging and unused product.
†Number of times menses in the control group: never 2 (11.8%), once 3 (17.8%), twice 11 (64.7%), and thrice 1 (5.9%).
‡Numbers of participants per randomisation group may very slightly due to loss to follow-up. Participants with ≥90% adherence not shown.
§Multiple answers possible.
¶Participant reported vulval itching and burning when passing urine.
‖Participant reported mild gastritis and wanting to withdraw from the study anyway.
**Participant reported receiving oral metronidazole therapy for 7 days due to infection.
††Participant reported having menses twice in one month; decided to use less of her product until the next study visit.
‡‡Participant reported genital itching, genital burning, and pain during sex.
§§One participant reported missing the D7 study visit and therefore running out of supplies. Another participant reported not to have used the study product during menses (which she was allowed to do).
¶¶Participant reported being drunk and therefore forgetting to take the study product.
‖‖Participant reported taking the study product correctly but that the product came out during menses
D7, Day 7 visit; EF+, Ecologic Femi+; Enr, enrolment visit; GynLP, Gynophilus LP; IQR, inter-quartile range; M1/2, Month 1/2 visit; NA, not applicable.

For peer review only - http://bmjopen.bmj.com/site/about/guidelines.xhtml

Acceptability

Ease-of-use

No participants reported having heard about probiotics before study participation. After product use, all vaginal probiotic users reported feeling very comfortable with insertion and that insertion became easier over time. All but one woman reported inserting while lying down (online supplementary material table 2).

Bodily changes and product perception

During FGDs, ~~some~~ several women using either vaginal probiotic reported the product (partially) "*coming out*" during the first few uses, but that this decreased after having gained experience. Many ~~women~~ EF+ and GynLP users reported an increase in vaginal wetness, which was considered a positive attribute by most. Some women reported increased libido. For example, one EF+ user said: "*I felt a great desire to* [have] *sex again and again.*" In contrast, o~~O~~ne metronidazole user reported a decrease in libido. Most women believed that the vaginal probiotics decreased the recurrence of symptomatic BV (our preliminary efficacy data suggest that ~~they did~~BV incidence had in fact decreased),[16] and a few believed that they also prevented STI ~~transmission~~ acquisition (the trial had insufficient statistical power to assess this).

Support

One social harm related to vaginal probiotic use was reported: a GynLP user ~~participant~~ was verbally harassed by her partner and her sister because of her study participation, and opted to withdraw her informed consent. Reports of partner, family, and community support during the FGDs/IDIs were mixed: some women reported problems with loved ones. Negative reactions from male partners were more often based on suspicions about study participation than the products themselves. One ~~woman~~ EF+ user said: "*He* [her partner] *did not accept that. He asked me to go together with him to the clinic* [a local health centre] *and check if I am not HIV-positive*." Another participant using metronidazole mentioned wanting to join the study to her husband, who forbade her to participate. However, she decided to join anyway: "*he did not know that I was using the study product, because he had refused*

For peer review only

me to join [the] *study before… I used them* [the study products] *without informing him.*" All sex workers except one stated that they had not discussed study participation with male clients.

Worries and concerns

In the FGDs, one woman reported hearing rumours prior to enrolling that vaginal products "*can damage the uterus or cause tumours in the womb.*" However, most participants thought that vaginal probiotics would be acceptable to Rwandan women. One GynLP user argued: "*They* [already] *give us vaginal pills*", by which she meant vaginal medications for yeast infections. Some women were concerned about future product availability and pricing. They hoped that probiotics would be distributed cheaply through the Rwandan *Mutuelle* public health insurance because they would otherwise be inaccessible to many women. One ~~participant~~ metronidazole user was concerned about a limited applicability of probiotics because BV is not diagnosed by laboratory testing in Rwanda: "*They do not have adequate medical instruments to test diseases, you tell the physician how* […] *you feel and by guessing the disease, he gives you at least four medications, saying that you may have trichomonas, you may have syphilis, you may have gonorrhoea* [she refers to syndromic management.[20,21]] *At health centre-level they do not have medical equipment to test diseases, meaning that they will not know who to give that* [probiotic/antibiotic maintenance therapy] *medication.*"

Vaginal practices and sexual risk-taking

At enrolment, 49.3% of the women reported to never use products inside the vagina, and at Month 6, this increased to 81.5% (OR 5.2, 95% CI 1.96-17.34; table 2). During FGDs, some women understood that vaginal washing practices may increase the risk of vaginal infection, but others did not. A participant stated: "*You get them* [i.e., vaginal diseases] *anyway… whether you wash or not*". In one FGD, 10 of 11 participants stated having ceased vaginal practices thanks to the study counselling. It should be noted that in contrast to many other African populations, Rwandan women use vaginal practices to increase rather than reduce vaginal lubrication. Women mentioned the use of herbs (*umushishiro*), Vaseline, and oils for this purpose. Self-reported sexual risk taking by face-to-face

For peer review only - http://bmjopen.bmj.com/site/about/guidelines.xhtml

interview did not change over time, except for a significant reduction in reported numbers of sex partners in the previous month at Month 6 compared to enrolment. No women in FGDs/IDIs mentioned adopting safer sex practices (such as consistent condom use) in response to the counselling messages. During face-to-face interviews at the Month 2 visit, 12 of 15 women (80%) who had an uncircumcised main sex partner reported asking him to regularly clean his penis in the future (online supplementary material table 2). While most women in FGDs understood that using condoms and improved penile hygiene could reduce BV rates, some mentioned that they found it difficult to discuss these topics with male partners. One participant stated that this is especially difficult being a sex worker: "*a man gives you his own money and you start educating him to wash!*" However, another sex worker reported refusing sex with uncircumcised clients: "*you leave him, because he has a lot* [of] *germs*". Several women reported discussing circumcision with their partners; one participant reported telling her husband: "*It is better that you do circumcision because it is a good thing… you would get a chance of not contracting diseases.*"

For peer review only - http://bmjopen.bmj.com/site/about/guidelines.xhtml

Table 2: Changes in reported vaginal cleansing practices and (sexual) behaviour between the enrolment and the M6 visit.

Self-reported sociodemographic characteristics	Enr (n=71)	M6 (n=65)	OR (95% CI)* P value*
Reports using no products inside the vagina (other than for managing menses; all participants) n (%)	35 (49.3)	53 (81.5)	5.2 (1.96–17.34)<br><0.001
Reports using no products inside the vagina (other than for managing menses; controls and metronidazole users only)† n (%)	15 (44.1)	27 (79.4)	13.0 (1.95–552.5)<br>0.002
Reports using water only n (%)	23 (32.4)	10 (15.4)	0.37 (0.13–0.92)<br>0.029
Reports using water and soap n (%)	3 (4.2)	2 (3.1)	0.67 (0.06–5.82)<br>1.00
Reports using paper, cloth or cotton wool n (%)	9 (12.7)	0 (0)	0.13 (0.00–0.93)‡<br>0.008
Reports using traditional herbs, stones, powders as vaginal cleansing practice n (%)	1 (1.4)	1 (1.5)	1.00 (0.01–78.5)‡<br>1.00
Mean weekly frequency of vaginal practices (95% CI)	2.15 (0.97–3.34)	0.64 (0.18–1.11)	NA<br>0.328
Median number of sex partners in last month at baseline or per month during follow-up period (IQR)	5 (3–16)	2 (1–4)	NA<br><0.001
Any condom use reported in past two weeks (Enr) or since last study visit (M6), versus none n (%)	64 (90.1)	60 (92.3)	1.67 (0.32–10.7)<br>0.727
Reports exchanging sex for money/goods in past month (Enr) or since last study visit (M6) n (%)	65 (91.5)	58 (89.2)	0.80 (0.16–3.72)<br>1.00

*McNemar's OR and p-value for binary variables and Wilcoxon signed-rank test p-value for continuous variables, comparing the response at M6 with the response at Enr. ORs with 95% CI were also calculated for binary pre-post data.
†N=34.
‡To enable calculation of effect measures, a zero value was replaced by 1.
CI, confidence interval; Enr, enrolment visit; IQR, inter-quartile range; M6, Month 6 visit; NA, not applicable; OR, odds ratio.

Correlates of adherence

In bivariable mixed effects models including the probiotic groups only, no participant characteristics were significantly associated with perfect adherence (table 3). However, non-significant trends were observed. Younger age, asking many questions at enrolment, having menses during the previous study interval, and reporting urogenital symptoms were associated with a lower likelihood of perfect adherence. When including oral metronidazole users, menses was significantly associated with a lower likelihood of perfect adherence (p=0.008). There were no significant associations between randomisation group and perfect adherence.

For peer review only

Table 3: Participant characteristics associated with perfect adherence

Participant characteristics	EF+ and GynLP users		EF+, GynLP and oral metronidazole users	
	OR (95% CI)	P value	OR (95% CI)	P value
Randomisation group: GynLP versus EF+	0.68 (0.22–2.11)	0.505	ND	ND
Randomisation group:<br>- EF+ versus metronidazole<br>- GynLP versus metronidazole	ND	ND	0.53 (0.15–1.81)<br>0.36 (0.11–1.23)	0.308<br>0.103
Age in years: ≥30 years versus <30	2.66 (0.90–7.82)	0.076	1.60 (0.61–4.15)	0.336
Marital status:<br>- Married versus never married<br>- Divorced versus never married<br>- Widowed versus never married	0.97 (0.14–6.58)<br>1.18 (0.29–4.79)<br>ND	0.976<br>0.912<br>0.991	1.17 (0.20–6.99)<br>1.39 (0.42–4.57)<br>ND	0.865<br>0.586<br>0.990
At least some schooling versus no schooling	1.20 (0.59–2.45)	0.619	0.80 (0.22–2.95)	0.740
Number of sex partners last month: five or more versus four or less.	0.58 (0.18–1.83)	0.351	0.49 (0.17–1.37)	0.173
Exchanged sex for money/goods past month	ND	0.990	ND	0.986
Nurse reported participant asked questions at Enr<br>- Yes, many versus none<br>- Yes, a few versus none	0.19 (0.02–1.52)<br>0.83 (0.24–2.83)	0.116<br>0.761	0.15 (0.02–1.19)<br>0.83 (0.27–2.57)	0.072<br>0.744
Had menses during study visit interval	0.41 (0.14–1.20)	0.104	0.26 (0.09–0.70)	0.008
Reported alcohol consumption during study:<br>- Once or twice per week versus never<br>- More than twice per week versus never	0.54 (0.14–2.12)<br>0.92 (0.18–4.81)	0.373<br>0.920	0.34 (0.11–1.08)<br>0.81 (0.19–3.49)	0.068<br>0.774
Reported at least one urogenital symptom during study interval versus none	0.11 (0.01–1.56)	0.103	0.30 (0.04–2.16)	0.231
Reported at least one adverse event during study visit interval (excluding urogenital symptoms) versus none	0.43 (0.10–1.83)	0.253	0.55 (0.15–2.05)	0.371

Sociodemographic characteristics associated with perfect adherence in bivariable mixed effects models, in the enrolment–D7, D7–M1, and M1–M2 study visit intervals.
CI, confidence interval; D7, Day 7 visit; EF+, Ecologic Femi+; Enr, enrolment visit; GynLP, Gynophilus LP; M1, Month 1 visit; M2, Month 2 visit; ND, non-determinable; OR, odds ratio.

Vaginal infection knowledge

Almost all participants reported having heard of 'diseases of the vagina' and STIs before, but only 4.6% knew what bacteria were (table 4). The STIs most often spontaneously named (in numerical order) were HIV, gonorrhoea, and syphilis; only one participant reported having heard of BV. After having received an explanation about what BV is, only one woman reported ever having been diagnosed with BV. Most participants could name at least one cause or potential consequence of vaginal infections. Consequences wrongfully attributed to vaginal infections were death (4.6% of women), infant malformations (3.9%), and cervical cancer/tumours (3.1%).

For peer review only - http://bmjopen.bmj.com/site/about/guidelines.xhtml

Table 4: Vaginal infection kKnowledge ~~of BV and other STIs~~

	Recruitment (n=61)	Enrolment (n=70)	Total (n=131)
Median age (IQR)	32 (27–35)*	31 (27–35)	31 (27–35)
Has heard of diseases of the vagina before n (%)	60 (98.4)	70 (100)	130 (99.2)
Reports knowing what bacteria are before study n (%)	5 (8.2)	1 (1.4)	6 (4.6)
Reports having heard about STIs before study n (%)	61 (100)	70 (100)	131 (100)
If yes, spontaneously named, without probing† n (%)			
- HIV	58 (95.1)	65 (92.9)	123 (93.9)
- Gonorrhoea	58 (95.1)	65 (92.9)	123 (93.9)
- Syphilis	44 (72.1)	59 (84.3)	103 (78.7)
- Trichomoniasis	38 (62.3)	48 (68.6)	86 (65.7)
- Hepatitis	3 (4.9)	3 (4.3)	6 (4.6)
- Yeast infection	0	3 (4.3)	3 (2.3)
- BV	0	2 (2.9)	2 (1.5)
- Urinary tract infection	1 (1.6)	1 (1.4)	2 (1.5)
- Chlamydia	0	1 (1.4)	1 (0.8)
- Herpes	0	1 (1.4)	1 (0.8)
- HPV / cervical cancer	1 (1.6)	0	1 (0.8)
Reports having heard about BV before this study n (%)	1 (1.6)	0	1 (0.8)
Spontaneously reported reasons why women get vaginal disease, without probing† n (%)			
- Poor toilet hygiene	37 (60.7)	40 (57.1)	77 (58.8)
- Multiple sex partners	28 (45.9)	36 (51.4)	64 (48.9)
- After sex	25 (41.0)	30 (43.0)	55 (42.0)
- Dirty underwear	19 (31.2)	35 (50.0)	54 (41.2)
- Poor vaginal hygiene	26 (42.6)	22 (31.4)	48 (36.6)
- Poor penile hygiene of male partner(s)	4 (6.6)	17 (24.3)	21 (16.0)
- Traditional vaginal practices and washing	3 (4.9)	12 (17.1)	15 (11.5)
- New sex partner	6 (9.8)	3 (4.3)	9 (6.9)
- Use of contraception	1 (1.6)	3 (4.3)	4 (3.1)
- (Improper) use of sanitary pads or tampons	1 (1.6)	3 (4.3)	4 (3.1)
- Other	3 (4.9)‡	1 (1.4)§	4 (3.1)
- Cannot name any reasons	1 (1.6)	0	1 (0.8)
Spontaneously reported negative consequences of vaginal disease being named, without probing† n (%)			
- Foul smell from the vagina	30 (49.2)	39 (56.5)	69 (53.1)
- Difficulty getting pregnant	18 (29.5)	33 (47.8)	51 (39.2)
- Miscarriage	16 (26.2)	33 (47.8)	49 (37.7)
- Abnormal vaginal discharge	12 (19.7)	28 (40.6)	40 (30.8)
- Baby born too early	16 (26.2)	22 (31.9)	38 (29.2)
- Severe infection / fever of the woman	7 (11.5)	7 (10.1)	14 (10.8)
- Infection / fever of the newborn baby	5 (8.2)	3 (4.4)	8 (6.2)
- Itching	4 (6.6)	4 (5.8)	8 (6.2)
- Other consequences to the baby	3 (4.9)	3 (4.4)	6 (4.6)
- Cervical cancer or tumours	2 (3.3)	3 (4.4)	5 (3.9)
- Death	4 (6.6)	0	4 (3.1)
- HIV/STIs	1 (1.6)	3 (4.4)	4 (3.1)
- Pain during intercourse	0	3 (4.4)	3 (2.3)
- Cannot name any consequence	17 (27.9)	19 (27.5)	36 (27.7)

*One missing value.
†Open-ended question. Totals may be more than 100%.
‡Participants report: "If you are infected with STIs", sharing underwear, and unprotected sex.
§Participant reports: vaginal medicine.
BV, bacterial vaginosis; HPV, human papilloma virus; IQR, interquartile range; STI, sexually transmitted infection.

For peer review only

For peer review only - http://bmjopen.bmj.com/site/about/guidelines.xhtml

DISCUSSION

Several studies of different vaginal probiotics have been conducted, some of them in sub-Saharan Africa.[10–13] However, none reported in-depth acceptability and adherence data. Our study suggests high vaginal probiotic acceptability and adherence in high-risk Rwandan women. We found no statistically significant correlates of perfect adherence, partially due to limited statistical power, but younger age, asking many questions about product use at enrolment, current menses, and reporting urogenital symptoms showed trends towards a lower likelihood of perfect adherence. Vaginal probiotics are currently unavailable on the market in most African countries, and it is important to study acceptability in different target populations to inform product development and future marketing strategies.

We could not evaluate the impact of self-reported acceptability aspects on adherence because almost all women reported very high acceptability in face-to-face interviews throughout the trial. Such interviews are known to suffer from social desirability bias. However, women seemed to speak freely in the FGDs, and those data indicate that they did not have major issues with product attributes or insertion. However, some women reported difficulties due to lack of male partner support. The reported increase in vaginal wetness after probiotic insertion was not considered problematic, as lubrication during sex is preferred by most Rwandan men and women.[22] This might be different in other countries where dry sex is preferred.[23] We did find a non-significant lower adherence to GynLP compared to EF+, which might be explained by differences in formulation: GynLP forms a gel in the vagina whereas EF+ capsules merely release lyophilised bacteria. Previous research indicated high adherence to GynLP.[24] Unfortunately, the impact of these formulation differences was insufficiently probed during the FGDs. Participants indicated that they found the diary cards helpful in reminding them to use their products, and we believe that self-monitoring tools might indeed be helpful in maximising adherence.[25]

Our data suggest that counselling was partially effective in changing behaviours that increase BV risk. Significantly more women reported not engaging in vaginal practices at the end of the study, and most

For peer review only - http://bmjopen.bmj.com/site/about/guidelines.xhtml

women with uncircumcised steady male partners reported having discussed penile hygiene with them. However, many women mentioned in FGDs that they found it difficult to discuss condom use and penile hygiene with male partners, especially clients. Women reduced their sexual risks only to a limited extent during follow-up, reporting a reduction in numbers of sex partners but no differences in engaging in sex work and condom use in face-to-face interviews. While these results are encouraging, it is difficult to assess to what extent they were influenced by social desirability bias.

Our survey with women at recruitment sessions and enrolment visits showed that high-risk Rwandan women had heard of several STIs, but were generally unaware of BV, its causes and potential consequences, and what they can do to prevent it. Experiences with HIV show that public health interventions can only succeed if health care professionals and the public have sufficient knowledge of causes and consequences of disease.[26–28] High-risk Rwandan women (and health care professionals) should therefore be educated about BV.

Limitations

Our study had limited statistical power, and social desirability bias may have affected some of our results, as is often the case in studies of this nature. Additionally, it should be noted that product efficacy, availability and cost are important determinants of acceptability, and were not evaluated in our study, although preliminary efficacy results in this study were promising.[16] We could not directly compare experiences with, and opinions about, the two different vaginal probiotics because each woman used only one product and qualitative data depth was suboptimal. In the FGDs/IDIs, it was sometimes difficult to ascertain whether participants were referring to personal experiences, or to wider community perceptions. Strengths of our study include the use of a mixed-methods approach and triangulated adherence data.

CONCLUSIONS

The prevention of BV recurrence will likely have to include several components to be successful, such as improved diagnostics, treatments, and prophylactic products (for example probiotics), but also

For peer review only - http://bmjopen.bmj.com/site/about/guidelines.xhtml

412 improved information, education, and counselling messages targeted to at-risk women and their partners. The results of this study can be used to inform future product development, and to fine-tune counselling messages in future trials.

For peer review only

For peer review only - http://bmjopen.bmj.com/site/about/guidelines.xhtml

Acknowledgments We thank the study participants, the Rinda Ubuzima team and other colleagues in Rwanda and the United Kingdom who contributed, the funders of this study, as well as the Trial Steering Committee for trial oversight. We thank Biose and Winclove for the donation of study products.

Author contributions JvdW obtained the research funding and wrote the study protocol and data collection documents. AN, EL, SA, and JvdW were members of the Trial Steering Committee. SA, MU, MMU, and JvdW collected the primary data. MU and MMU performed the FGDs and IDIs. MV and JvdW developed the analytical approach and performed the statistical analyses. MV and JvdW wrote the manuscript. All authors commented on and approved the final manuscript.

Funding This work was funded by the DFID/MRC/Wellcome Trust Joint Global Health Trials Scheme as a Development Project (grant reference MR/M017443/1; grant title: "Preparing for a clinical trial of interventions to maintain normal vaginal microbiota for preventing adverse reproductive health outcomes in Africa"). Vaginal probiotics for use in the trial were donated free of charge by Winclove Probiotics (Amsterdam, The Netherlands) and Biose (formerly Probionov; Aurillac, France). The findings and conclusions in this paper are those of the authors and do not necessarily represent the views of the authors' institutions or companies, or the funder. None of the authors were paid to write this article. The corresponding author had full access to the data and had final responsibility for the decision to submit for publication.

Competing interests AN is employed by Biose (owner of trial product GynLP) and EL by Winclove Probiotics BV (owner of trial product EF+). AN has financial and/or intellectual investments in competing products. The other authors report no competing interests.

Patient consent All participants provided written informed consent for study participation, and separate written informed consent for participation in FGDs/IDIs.

For peer review only - http://bmjopen.bmj.com/site/about/guidelines.xhtml

For peer review only

442 **Ethics approval** The study was sponsored by the University of Liverpool, approved by the Rwanda National Ethics Committee and the University of Liverpool Research Ethics Subcommittee for Physical Interventions, and registered on ClinicalTrials.gov (NCT02459665).

Data sharing statement The data supporting the findings of this publication are retained by the corresponding author (JvdW) and will not be made openly accessible due to privacy concerns. Fully anonymised data can be made available by written request to j.vandewijgert@liverpool.ac.uk after assurance that the intended data usage is compliant with relevant ethical approvals and privacy will be maintained.

For peer review only

For peer review only - http://bmjopen.bmj.com/site/about/guidelines.xhtml

Figure footnotes

Figure 1: Flowchart of the study

*Totals to 110 reasons among 102 women because there could be more than one reason per woman.
†Reasons: outside of metronidazole treatment window (n=5), enrolment target already met (n=4), has a mental disorder (n=1), did not complete screening procedures and was subsequently lost to follow=up (n=1), withdrew consent during the screening visit because she thought the reimbursement was too low (n=1).
‡Reasons: moved away from Kigali (n=2), lost interest because symptoms resolved (n=1), and was verbally harassed by partner and sister about study participation (n=1).

Acceptability assessments were made at enrolment and at the M2 visit. Adherence assessments were made using self-rated assessments, pictorial diary cards, and returned packaging at the D7, M1, and M2 visits (after which product use was ceased). The vaginal infection knowledge survey was held at recruitment sessions in the community and at the enrolment visit. Changes in sexual risk-taking and vaginal practices were assessed at each follow-up visits and compared to answers given during the enrol visit. All this themes were discussed during the eight FGDs and IDIs.

BV, bacterial vaginosis; D7, day 7 visit; FGD, focus group discussion; IDI, in-depth interview; M1/2/6, month 1/2/6 visit; RU, Rinda Ubuzima; TV, *Trichomonas vaginalis*.

For peer review only

For peer review only - http://bmjopen.bmj.com/site/about/guidelines.xhtml

References

1 van de Wijgert JHHM, Borgdorff H, Verhelst R, *et al.* The vaginal microbiota: what have we learned after a decade of molecular characterization? *PLOS ONE* 2014;**9**:e105998. doi:10.1371/journal.pone.0105998

2 van de Wijgert JHHM, Morrison CS, Cornelisse PGA, *et al.* Bacterial vaginosis and vaginal yeast, but not vaginal cleansing, increase HIV-1 acquisition in African women. *J Acquir Immune Defic Syndr 1999* 2008;**48**:203–10. doi:10.1097/QAI.0b013e3181743936

3 van de Wijgert JHHM. The vaginal microbiome and sexually transmitted infections are interlinked: consequences for treatment and prevention. *PLOS Med* 2017;**14**:e1002478. doi:10.1371/journal.pmed.1002478

4 Li J, McCormick J, Bocking A, *et al.* Importance of vaginal microbes in reproductive health. *Reprod Sci* 2012;**19**:235–42. doi:10.1177/1933719111418379

5 Nelson DB, Hanlon AL, Wu G, *et al.* First trimester levels of BV-associated bacteria and risk of miscarriage among women early in pregnancy. *Matern Child Health J* 2015;**19**:2682–7. doi:10.1007/s10995-015-1790-2

6 Hay P. Recurrent bacterial vaginosis. *Curr Opin Infect Dis* 2009;**22**:82–6. doi:10.1097/QCO.0b013e32832180c6

7 Verstraelen H, Verhelst R. Bacterial vaginosis: an update on diagnosis and treatment. *Expert Rev Anti Infect Ther* 2009;**7**:1109–24. doi:10.1586/eri.09.87

8 Torrone EA, Morrison CS, Chen P-L, *et al.* Prevalence of sexually transmitted infections and bacterial vaginosis among women in sub-Saharan Africa: An individual participant data meta-analysis of 18 HIV prevention studies. *PLOS Med* 2018;**15**:e1002511. doi:10.1371/journal.pmed.1002511

9 Bradshaw CS, Brotman RM. Making inroads into improving treatment of bacterial vaginosis – striving for long-term cure. *BMC Infect Dis* 2015;**15**:292. doi:10.1186/s12879-015-1027-4

10 Anukam KC, Osazuwa E, Osemene GI, *et al.* Clinical study comparing probiotic *Lactobacillus* GR-1 and RC-14 with metronidazole vaginal gel to treat symptomatic bacterial vaginosis. *Microbes Infect* 2006;**8**:2772–6. doi:10.1016/j.micinf.2006.08.008

11 Ling Z, Liu X, Chen W, *et al.* The restoration of the vaginal microbiota after treatment for bacterial vaginosis with metronidazole or probiotics. *Microb Ecol* 2013;**65**:773–80. doi:10.1007/s00248-012-0154-3

12 Ngugi BM, Hemmerling A, Bukusi EA, *et al.* Effects of bacterial vaginosis-associated bacteria and sexual intercourse on vaginal colonization with the probiotic *Lactobacillus crispatus* CTV-05. *Sex Transm Dis* 2011;**38**:1020–7. doi:10.1097/OLQ.0b013e3182267ac4

13 Bradshaw CS, Pirotta M, De Guingand D, *et al.* Efficacy of oral metronidazole with vaginal clindamycin or vaginal probiotic for bacterial vaginosis: randomised placebo-

For peer review only - http://bmjopen.bmj.com/site/about/guidelines.xhtml

controlled double-blind trial. *PLoS ONE* 2012;**7**:e34540. doi:10.1371/journal.pone.0034540

14 van der Straten A, Montgomery ET, Cheng H, *et al.* High acceptability of a vaginal ring intended as a microbicide delivery method for HIV prevention in African women. *AIDS Behav* 2012;**16**:1775–86. doi:10.1007/s10461-012-0215-0

15 Merkatz RB, Plagianos M, Hoskin E, *et al.* Acceptability of the nestorone®/ethinyl estradiol contraceptive vaginal ring: development of a model; implications for introduction. *Contraception* 2014;**90**:514–21. doi:10.1016/j.contraception.2014.05.015

16 van de Wijgert JHHM, Verwijs MC, Agaba SK, *et al*. Intermittent use of vaginal probiotics or oral metronidazole to prevent bacterial vaginosis recurrence: safety and preliminary efficacy by Nugent scoring and 16S rRNA gene sequencing. 2018. Conference talk presented at the Keystone Symposium "Role of the genital tract microbiome in sexual and reproductive health", Cape Town, South Africa.

17 Sobel JD, Ferris D, Schwebke J, *et al.* Suppressive antibacterial therapy with 0.75% metronidazole vaginal gel to prevent recurrent bacterial vaginosis. *Am J Obstet Gynecol* 2006;**194**:1283–9. doi:10.1016/j.ajog.2005.11.041

18 McClelland RS, Richardson BA, Hassan WM, *et al.* Improvement of vaginal health for Kenyan women at risk for acquisition of Human Immunodeficiency Virus type 1: results of a randomized trial. *J Infect Dis* 2008;**197**:1361–8. doi:10.1086/587490

19 Kestelyn E, Nuil JIV, Umulisa MM, *et al.* High acceptability of a contraceptive vaginal ring among women in Kigali, Rwanda. *PLOS ONE* 2018;**13**:e0199096. doi:10.1371/journal.pone.0199096

20 World Health Organization. Guidelines for the management of sexually transmitted infections. 2003.http://applications.emro.who.int/aiecf/web79.pdf (accessed 12 Mar 2019).

21 Binagwaho A, editor. *National guidelines for prevention and management of HIV, STIs & other blood borne infections*. Republic of Rwanda - Ministry of Health 2013. https://aidsfree.usaid.gov/sites/default/files/hts_policy_rwanda.pdf (accessed 12 Mar 2019).

22 Veldhuijzen N, Nyinawabega J, Umulisa M, *et al.* Preparing for microbicide trials in Rwanda: focus group discussions with Rwandan women and men. *Cult Health Sex* 2006;**8**:395–406. doi:10.1080/13691050600859302

23 Low N, Chersich MF, Schmidlin K, *et al.* Intravaginal practices, bacterial vaginosis, and HIV infection in women: individual participant data meta-analysis. *PLOS Med* 2011;**8**:e1000416. doi:10.1371/journal.pmed.1000416

24 Dausset C, Patrier S, Gajer P, *et al.* Comparative phase I randomized open-label pilot clinical trial of Gynophilus® (Lcr regenerans®) immediate release capsules versus slow release muco-adhesive tablets. *Eur J Clin Microbiol Infect Dis* 2018;**37**:1869–80. doi:10.1007/s10096-018-3321-8

For peer review only - http://bmjopen.bmj.com/site/about/guidelines.xhtml

25 Safren SA, W. Otto M, Worth JL, *et al.* Two strategies to increase adherence to HIV antiretroviral medication: Life-Steps and medication monitoring. *Behav Res Ther* 2001;**39**:1151–62. doi:10.1016/S0005-7967(00)00091-7

26 Glick P, Sahn DE. Changes in HIV/AIDS knowledge and testing behavior in Africa: how much and for whom? *J Popul Econ* 2007;**20**:383–422. doi:10.1007/s00148-006-0085-8

27 Peltzer K, Matseke G, Mzolo T, *et al.* Determinants of knowledge of HIV status in South Africa: results from a population-based HIV survey. *BMC Public Health* 2009;**9**:174. doi:10.1186/1471-2458-9-174

28 Fay H, Baral SD, Trapence G, *et al.* Stigma, health care access, and HIV knowledge among men who have sex with men in Malawi, Namibia, and Botswana. *AIDS Behav* 2011;**15**:1088–97. doi:10.1007/s10461-010-9861-2

For peer review only

For peer review only - http://bmjopen.bmj.com/site/about/guidelines.xhtml

Vaginal Probiotic Adherence and Acceptability in Rwandan Women with High Sexual Risk Participating in a Pilot Randomised Controlled Trial: A Mixed-Methods Approach

Journal:	*BMJ Open*
Manuscript ID	bmjopen-2019-031819.R1
Article Type:	Original research
Date Submitted by the Author:	23-Jan-2020
Complete List of Authors:	Verwijs, Marijn; Institute of Infection and Global Health, University of Liverpool,<br>Agaba, Stephen; Rinda Ubuzima<br>Umulisa, Marie ; Rinda Ubuzima<br>Uwineza, Mireille; Rinda Ubuzima<br>Nivoliez, Adrien; Biose,<br>Lievens, Elke; Winclove<br>van de Wijgert, Janneke H.H.M.; University of Liverpool, Institute of Infection and Global Health; Universitair Medisch Centrum Utrecht, Julius Center for Health Sciences and Primary Care
**Primary Subject Heading**:	Sexual health
Secondary Subject Heading:	Sexual health, Obstetrics and gynaecology, Public health, Qualitative research
Keywords:	bacterial vaginosis, vaginal probiotic, adherence, acceptability, Africa

I, the Submitting Author has the right to grant and does grant on behalf of all authors of the Work (as defined in the below author licence), an exclusive licence and/or a non-exclusive licence for contributions from authors who are: i) UK Crown employees; ii) where BMJ has agreed a CC-BY licence shall apply, and/or iii) in accordance with the terms applicable for US Federal Government officers or employees acting as part of their official duties; on a worldwide, perpetual, irrevocable, royalty-free basis to BMJ Publishing Group Ltd ("BMJ") its licensees and where the relevant Journal is co-owned by BMJ to the co-owners of the Journal, to publish the Work in this journal and any other BMJ products and to exploit all rights, as set out in our licence.

The Submitting Author accepts and understands that any supply made under these terms is made by BMJ to the Submitting Author unless you are acting as an employee on behalf of your employer or a postgraduate student of an affiliated institution which is paying any applicable article publishing charge ("APC") for Open Access articles. Where the Submitting Author wishes to make the Work available on an Open Access basis (and intends to pay the relevant APC), the terms of reuse of such Open Access shall be governed by a Creative Commons licence – details of these licences and which Creative Commons licence will apply to this Work are set out in our licence referred to above.

Other than as permitted in any relevant BMJ Author's Self Archiving Policies, I confirm this Work has not been accepted for publication elsewhere, is not being considered for publication elsewhere and does not duplicate material already published. I confirm all authors consent to publication of this Work and authorise the granting of this licence.

For peer review only

Vaginal Probiotic Adherence and Acceptability in Rwandan Women with High Sexual Risk Participating in a Pilot Randomised Controlled Trial: A Mixed-Methods Approach

Authors: Marijn C. Verwijs (MD)[1], Stephen K. Agaba (MD)[2], Marie-Michele Umulisa (MA)[2], Mireille Uwineza (BA)[2], Adrien Nivoliez (PhD)[3], Elke Lievens (PhD)[4], Janneke H.H.M. van de Wijgert (MD PhD MPH)[1,5].

Author affiliations

[1]Institute of Infection and Global Health, University of Liverpool, Liverpool, United Kingdom.

[2]Rinda Ubuzima, Kigali, Rwanda.

[3]Biose, Aurillac, France.

[4]Winclove Probiotics, Amsterdam, The Netherlands.

[5]Julius Center for Health Sciences and Primary Care, University Medical Center Utrecht, Utrecht University, Utrecht, The Netherlands.

Correspondence to

Professor Janneke van de Wijgert, MD PhD MPH

Department of Clinical Infection, Microbiology and Immunology

Institute of Infection and Global Health, University of Liverpool

Ronald Ross Building, 8 West Derby Street, Liverpool L69 7BE, United Kingdom

Email: j.vandewijgert@liverpool.ac.uk

ORCID: 0000-0003-2728-4560

Word counts:

Word count abstract: 300 words (max. 300 words).

Word count main text: 3,911 words (max. 4000 words).

Number of tables and figures: 5 (max. 5).

Number of supplementary tables and figures: 3.

For peer review only - http://bmjopen.bmj.com/site/about/guidelines.xhtml

For peer review only

ABSTRACT

Objectives To evaluate adherence and acceptability of intermittent vaginal probiotic or antibiotic use to prevent bacterial vaginosis (BV) recurrence.

Design Repeated adherence and acceptability assessments using mixed methods within a pilot randomised controlled trial.

Setting Research clinic in Kigali, Rwanda.

Participants Rwandan women with high sexual risk.

Interventions Women diagnosed with BV and/or trichomoniasis were randomised to four groups (n=17 each) after completing metronidazole treatment: behavioural counselling only, or behavioural counselling plus two-month intermittent use of oral metronidazole, Ecologic Femi+ (EF+) vaginal capsule, or Gynophilus LP (GynLP) vaginal tablet.

Outcome measures Adherence and acceptability were assessed by structured face-to-face interviews, semi-structured focus group discussions and in-depth interviews, daily diaries, and counting of used/unused study products in randomised women (n=68). Vaginal infection knowledge was assessed by structured face-to-face interviews in randomised women and women attending recruitment sessions (n=131).

Results Most women (93%) were sex workers, 99.2% were unfamiliar with BV, and none had ever used probiotics. All probiotic users (n=32) reported that insertion became easier over time. Triangulated adherence data showed that 17/17 EF+ users and 13/16 GynLP users used ≥80% of required doses (Fisher's exact p=0.103). Younger age (p=0.076), asking many questions at enrolment (p=0.116), having menses (p=0.104), and reporting urogenital symptoms (p=0.103) were non-significantly associated with lower perfect adherence. Women believed that the probiotics reduced BV recurrence, but reported that partners were sometimes unsupportive of study participation. Self-reported vaginal washing practices decreased during follow-up, but sexual risk behaviours did not. Most women (12/15) with an uncircumcised steady partner discussed penile hygiene with him, but many women found this difficult, especially with male clients.

For peer review only - http://bmjopen.bmj.com/site/about/guidelines.xhtml

Conclusions High-risk women require education about vaginal infections. Vaginal probiotic acceptability and adherence were high in this cohort. Our results can be used to inform future product development and to fine-tune counselling messages in prevention programs.

Trial registration ClinicalTrials.gov (NCT02459665).

Keywords (5) bacterial vaginosis, vaginal probiotic, adherence, acceptability, Africa.

ARTICLE SUMMARY

Strengths and limitations of this study

- We conducted this research in the context of a pilot randomised controlled trial, and statistical power was therefore limited.
- We triangulated different sources of adherence data to maximise accuracy, and used a mixed-methods approach to evaluate acceptability.
- We could not directly compare experiences with, and opinions about, the two different vaginal probiotics because each woman used only one product and qualitative data depth was suboptimal.
- Social desirability bias may have affected some of the results.
- The results of this study may not be generalizable to women at lower risk of sexually transmitted or urogenital infections.

For peer review only

For peer review only - http://bmjopen.bmj.com/site/about/guidelines.xhtml

INTRODUCTION

Bacterial vaginosis (BV) is a vaginal condition in which fastidious anaerobes such as *Gardnerella vaginalis* increase while beneficial, lactic acid-producing lactobacilli decrease.[1] Often asymptomatic, it is associated with increased risks of sexually transmitted infections (STIs) and HIV acquisition, pelvic inflammatory disease, and adverse pregnancy outcomes.[2–5] Although BV is treatable with antibiotics, the risk of recurrence is high.[6,7] The prevalence of BV varies among regions and ethnic groups but is highest in sub-Saharan Africa, where it is estimated at 30-50%.[8]

Vaginally-administered probiotics containing lactobacilli are considered a promising new strategy to restore a lactobacilli-dominated vaginal microbiota during and/or after antibiotic treatment, or to prevent BV.[9] While some probiotics have been available on the market for several years, clinical trials to support beneficial effects have only recently been initiated for most products.[10–13] Future uptake and adherence of a vaginal probiotic, once proven efficacious, is determined to a large extent by its acceptability in target populations. The acceptability, in turn, depends on factors such as characteristics of the target population, characteristics of and experiences with the product, types of sexual relationships and partner support, and community perceptions.[14,15]

We conducted a clinical trial of intermittent use of two vaginal probiotics and oral metronidazole to prevent BV recurrence in Rwandan women who had been treated for BV and/or *Trichomonas vaginalis* (TV). We used qualitative and quantitative research methods to assess adherence and acceptability with vaginal probiotic use. We triangulated various sources of adherence data to obtain adherence estimates per woman for each period of intermittent product use in between study visits, and determined correlates of adherence.

METHODS

The pilot clinical trial took place from June 2015 to February 2016 at the Rinda Ubuzima research clinic in Kigali, Rwanda. The trial was a pilot trial with a modest sample size at the request of the funder. Women who had been successfully treated for BV/TV with a seven-day course of oral

For peer review only

For peer review only - http://bmjopen.bmj.com/site/about/guidelines.xhtml

metronidazole (Tricozole, Laboratory & Allied Ltd, Nairobi, Kenya) were randomised to four intervention groups (n=17 each) to prevent BV recurrence: behavioural counselling only (controls), or behavioural counselling plus intermittent use of two different vaginal probiotics or oral metronidazole for two months. The behavioural counselling included counselling on safer sex, vaginal hygiene (including discouragement of intravaginal washing), and penile hygiene (i.e. encouragement of cleansing the penis, including underneath the foreskin), because these behaviours are known to reduce BV recurrence risk somewhat.[6,16] We counselled all women in all randomisation groups because we considered it unethical to withhold this information from women at risk. Women were seen at screening, enrolment (product use initiation, if applicable), Day 7, Month 1, Month 2 (product use cessation, if applicable), and Month 6. Product efficacies were not known during the trial, and the efficacy results of the pilot trial are reported elsewhere.[17] Briefly, the vaginal probiotics did improve the vaginal environment (increased lactobacilli and reduced BV-associated bacteria) compared to counselling only, but not as much as oral metronidazole did.

Study population

Women aged 18-45 at risk of HIV/STIs (defined as having had more than one sex partner and/or having been treated for an STI and/or BV in the last 12 months) were eligible for enrolment if they were confirmed HIV-negative, non-pregnant, diagnosed with BV and/or TV, and cured after seven-day oral metronidazole treatment. Other clinical exclusion criteria were applied but were rare.[17] Women were recruited by study staff with the assistance of Community Mobilisers who had strong ties with local high-risk women (particularly sex workers).

Study products and dosing

Ecologic Femi+ (EF+; Winclove Probiotics, Amsterdam, Netherlands) is a vaginal capsule containing lyophilised lactic acid-producing bacteria. EF+ was used once per day for five days followed by thrice weekly, for two months. Gynophilus LP (GynLP; Biose, Aurillac, France) is a tablet containing the *Lactobacillus rhamnosus* Lcr35 strain. The tablet disintegrates in the vagina and forms a gel that slowly releases the probiotic bacteria. GynLP was used once every four days for two months. The first

For peer review only - http://bmjopen.bmj.com/site/about/guidelines.xhtml

dose was inserted at the clinic under direct observation of a clinician, and remaining doses were self-administered at home. Women were asked not to cleanse or insert other products into the vagina after probiotic insertion to allow the probiotics to dissolve. They were also told that they were allowed to cease probiotic use during menses, but were encouraged to continue. Intermittent metronidazole use was chosen as a positive control intervention because studies conducted in the U.S. and Kenya have shown a 30-40% reduction in BV recurrence.[18,19] Metronidazole users took 500 mg generic oral metronidazole (Laboratory & Allied ltd, Nairobi, Kenya) twice weekly for two months. The rationale for selecting these study products and their dosing schedules can be found in the manuscript describing the efficacy results of the pilot trial.[17] Participants and clinicians were not blinded.

Acceptability, adherence, behavioural, and vaginal infection knowledge assessments

Acceptability was assessed at the enrolment visit prior to product use initiation and at the Month 2 visit after the full two months of use. Adherence was assessed during the intervention period, at the Day 7, Month 1, and Month 2 visits. Sexual and other behaviours were assessed at all study visits. Participants were interviewed face-to-face in Kinyarwanda by a trained study nurse using structured questionnaires with multiple-choice questions, questions requiring a number or date, and an adherence self-rating scale (from 0-10). In between visits, participants used pictorial diary cards (online supplementary material figure 1) to record daily episodes of product use, vaginal sex, condom use, and vaginal practices. Those using study products returned the product packaging and unused products (if applicable) to their clinic visits, where they were counted by study staff. Any discrepancies between data sources were discussed with participants and the consensus assessments were recorded on the questionnaires. The adherence data based on the self-rating scale, the diary card, and the returned product packaging were triangulated by the data analyst at the data analysis stage. Additionally, 131 women were interviewed about their knowledge of vaginal infections (such as BV and STIs) using a structured questionnaire during recruitment sessions (n=61; regardless of eligibility) and at enrolment visits (n=70; this included the 68 randomised women, and two women who attended enrolment visits but turned out to be ineligible; figure 1). Women were interviewed before being counselled at study visits or before receiving information at recruitment sessions. This questionnaire

For peer review only

For peer review only - http://bmjopen.bmj.com/site/about/guidelines.xhtml

contained multiple-choice and open-ended questions. Responses to the open-ended questions were categorised and discussed by two different researchers until consensus about the answer categories was reached.

Four semi-structured focus group discussions (FGDs) with 7-11 participants per group (total n=38), and semi-structured individual in-depth interviews (IDIs) with four additional participants, were held. The main themes of these FGDs and IDIs were experiences with and opinions of the study products, sexual behaviour, and vaginal practices. Women randomised to the behavioural counselling only group were not approached for the FGDs and IDIs, but all other randomised participants who had completed their product use period were approached until data saturation had been achieved. The interviews were unlinked anonymous, and women used pseudonyms to enable them to talk freely despite the fact that the discussions and interviews were taped. All interviews took place between November 2015 and March 2016, were held in Kinyarwanda, recorded on tape, transcribed verbatim, and translated into English. The FGD and IDI transcripts were read and discussed by three researchers (MV, MU, and JvdW) at regular intervals. The Chief Investigator (JvdW) decided that data saturation had been met when the fourth FGD and the fourth IDI transcript had become available in March 2016.

Data analysis

The primary outcomes of this study were acceptability and triangulated adherence in women randomised to study product use. Secondary outcomes included vaginal infection knowledge of the target population more broadly, and behavioural changes (of the behaviours included in the counselling messages) in all randomised women. Questionnaire data were analysed using Stata 13 (StataCorp, College Station, TX, USA). The proportion of women with ≥80%/≥90%/100% adherence in the probiotic groups were compared by Fisher's exact tests. Changes in self-reported vaginal practices and sexual behaviours over time were tested using McNemar's test for binary outcomes, and Wilcoxon's signed-rank test for continuous outcomes. To study associations of participant characteristics with triangulated adherence, we used bivariable mixed effects models, with perfect adherence (defined as having used all doses as instructed) per interval between study visits during the

For peer review only - http://bmjopen.bmj.com/site/about/guidelines.xhtml

intervention period as the outcome, participant identification numbers as the random effect, and one participant characteristic at the time as the fixed effect. We could not determine correlates of acceptability due to limited variation in the acceptability data (reported acceptability was high throughout the trial).

The FGD and IDI transcripts were coded using NVivo 10.0 (QSR International, Melbourne, Australia) by one single researcher (MV). The discussions and interviews were semi-structured, with the above-mentioned themes and associated codes prepared a priori, as well as new elements that emerged from the data. The codes were derived from an acceptability framework that has been used in studies of vaginal products for contraception or HIV prevention.[14,15,20] Components of the framework include study population characteristics, product attributes, sexual encounter and relational attributes, and the contextual environment (e.g. community perceptions of product use).

Ethical statement

All participants provided written consent for study participation, and separate consent for participation in FGDs/IDIs. All non-married participants aged 18-20 also required parental/guardian consent per Rwandan law at the time of the study. The participants received 3 GBP per visit (in local currency) as a reimbursement for time and transport costs. Care was taken to protect participant privacy and confidentiality. The study was sponsored by the University of Liverpool, approved by the Rwanda National Ethics Committee and the University of Liverpool Research Ethics Subcommittee for Physical Interventions, and registered on ClinicalTrials.gov (NCT02459665).

Participant and public involvement

As part of the FGDs/IDIs, a subset of the enrolled participants were invited to comment on study design and experiences with the interventions. Participants were not invited to develop outcomes, interpret the results, or to contribute to the writing or editing of this document for readability or accuracy. The preliminary results of this study were discussed with 32 stakeholders during a workshop held at the Ministry of Health in Kigali, Rwanda, in December 2017. These stakeholders

For peer review only - http://bmjopen.bmj.com/site/about/guidelines.xhtml

included representatives of the Ministry of Health, the National University of Rwanda, the National Ethics Committee, local hospitals and clinics, and local non-governmental and women's organisations.

RESULTS

Baseline characteristics

We screened 176 women: bacterial STI prevalence was 31.3% and BV prevalence by Gram stain Nugent scoring was 47.9%. All 68 randomised women were treated for BV and/or TV prior to randomisation and at risk of HIV/STIs, with 93.1% reporting having exchanged sex for money and/or goods in the previous month (figure 1, online supplementary material table 1). We collected 29.93 person-years of data. Four women withdrew their informed consent during the study (for reasons unrelated to study product acceptability). None were lost to follow-up.

Adherence

Triangulated adherence was high: 17/17 (100%) of EF+ users and 13/16 (81.3%) of GynLP users used ≥80% of required doses (Fisher's exact p=0.103; table 1), and these percentages were 15/17 (88.2%) and 11/16 (68.8%) for ≥90% (p=0.225), and 10/17 (58.8%) and 8/16 (50%) for 100% of required doses (p=0.732), respectively. In comparison, these percentages were 15/17 (88.2%), 14/17 (82.4%), and 12/17 (70.6%), respectively, for oral metronidazole users. Reported reasons of non-adherence to vaginal probiotics during face-to-face interviews were 'simply forgetting' (n=9), experiencing side-effects (n=2), menses (n=2), and being away from home and having left products at home (n=1). Additional reasons for missing doses mentioned during FGDs/IDIs were being drunk (n=2) and being confused about the dosing schedule (n=2). Only one woman in the metronidazole arm reported missing doses due to experiencing side-effects. Most women in FGDs reported using all doses as instructed and finding it easy to adhere, and thought that the diary cards served as a useful reminder to use the products.

For peer review only - http://bmjopen.bmj.com/site/about/guidelines.xhtml

Table 1: Adherence to study interventions

**Adherence to study products**	**Metronidazole (n=17)**	**EF+ (n=17)**	**GynLP (n=16)**
Adherence Enr–D7, median % (IQR)	100 (100–100)	100 (100–100)	100 (100–100)
Adherence D7–M1, median % (IQR)	100 (100–100)	100 (100–100)	100 (91.7–100)
Adherence M1–M2, median % (IQR)	100 (100–100)	100 (100–100)	100 (92.3–100)
Overall adherence Enr–M2, median % (IQR)	100 (96.3–100)	100 (100–100)	98.3 (89.3–100)
Overall adherence Enr–M2 n (%)<br>- Perfect*<br>- Adherence ≥90%<br>- Adherence ≥80%	 12 (70.6)<br>14 (82.4)<br>15 (88.2)	 10 (58.8)<br>15 (88.2)<br>17 (100)	 8 (50.0)<br>11 (68.8)<br>13 (81.3)
Number of times menses Enr–M2 n (%)†<br>- Never<br>- Once<br>- Twice	 7 (41.2)<br>6 (35.3)<br>4 (23.5)	 4 (23.5)<br>5 (29.4)<br>8 (47.1)	 2 (12.5)<br>4 (25.0)<br>10 (62.5)
Did not use product during menses at least once n (%)<br>- Yes<br>- NA (never had menses)	 4 (23.5)<br>7 (41.2)	 3 (17.6)<br>4 (23.5)	 5 (31.3)<br>2 (12.5)
**Self-reported reasons for non-adherence‡**	**Metronidazole**	**EF+**	**GynLP**
D7: Self-reported reasons why not able to use all doses as instructed n (%)§<br>- Simply forgot<br>- Product had side effects	 0<br>0	 2 (11.8)<br>0	 0<br>1 (6.7)¶
M1: Self-reported reasons why not able to use all doses as instructed n (%)§<br>- Simply forgot<br>- Product had side effects<br>- Did not like product for another reason<br>- Other	 1 (6.3)<br>1 (6.3)|| 1 (6.3)|| 1 (6.3)**	 1 (5.9)<br>0<br>0<br>1 (5.9)††	 1 (6.3)<br>1 (6.3)‡‡<br>0<br>2 (12.5)§§
M2: Self-reported reasons why not able to use all doses as instructed n (%)§<br>- Simply forgot<br>- Travelled and forgot to take product<br>- Other	 1 (6.3)<br>1 (6.3)<br>0	 2 (11.8)<br>0<br>1 (5.9)¶¶	 3 (18.8)<br>1 (6.25)<br>1 (6.3)||||
D7: Participant thinks she used product correctly most of the time n (%)	17 (100)	16 (94.1)	14 (93.3)
M1: Participant thinks she used product correctly most of the time n (%)	13 (86.7)	17 (100)	11 (68.8)
M2: Participant thinks she used product correctly most of the time n (%)	15 (93.7)	16 (94.1)	14 (87.5)

*Defined as 100% of the prescribed doses used at the prescribed times after nurse review of the participant's diary card and returned used packaging and unused product.
†Number of times menses in the control group: never 2 (11.8%), once 3 (17.8%), twice 11 (64.7%), and thrice 1 (5.9%).
‡Numbers of participants per randomisation group may very slightly due to loss to follow-up. Participants with ≥90% adherence not shown.
§Multiple answers possible.
¶Participant reported vulval itching and burning when passing urine.
||Participant reported mild gastritis and wanting to withdraw from the study anyway.
**Participant reported receiving oral metronidazole therapy for 7 days due to infection.
††Participant reported having menses twice in one month; decided to use less of her product until the next study visit.
‡‡Participant reported genital itching, genital burning, and pain during sex.
§§One participant reported missing the D7 study visit and therefore running out of supplies. Another participant reported not to have used the study product during menses (which she was allowed to do).
¶¶Participant reported being drunk and therefore forgetting to take the study product.
||||Participant reported taking the study product correctly but that the product came out during menses
D7, Day 7 visit; EF+, Ecologic Femi+; Enr, enrolment visit; GynLP, Gynophilus LP; IQR, inter-quartile range; M1/2, Month 1/2 visit; NA, not applicable.

For peer review only - http://bmjopen.bmj.com/site/about/guidelines.xhtml

Acceptability

Ease-of-use

No participants reported having heard about probiotics before study participation. After product use, all vaginal probiotic users reported feeling very comfortable with insertion and that insertion became easier over time. All but one woman reported inserting while lying down (online supplementary material table 2).

Bodily changes and product perception

During FGDs, several women using either vaginal probiotic reported the product (partially) "*coming out*" during the first few uses, but that this decreased after having gained experience. Many EF+ and GynLP users reported an increase in vaginal wetness, which was considered a positive attribute by most. Some women reported increased libido. For example, one EF+ user said: "*I felt a great desire to* [have] *sex again and again.*" In contrast, one metronidazole user reported a decrease in libido. Most women believed that the vaginal probiotics decreased the recurrence of symptomatic BV (our preliminary efficacy data suggest that BV incidence had in fact decreased),[17] and a few believed that they also prevented STI acquisition (the trial had insufficient statistical power to assess this).

Support

One social harm related to vaginal probiotic use was reported: a GynLP user was verbally harassed by her partner and her sister because of her study participation, and opted to withdraw her informed consent. Reports of partner, family, and community support during the FGDs/IDIs were mixed: some women reported problems with loved ones. Negative reactions from male partners were more often based on suspicions about study participation than the products themselves. One EF+ user said: "*He* [her partner] *did not accept that. He asked me to go together with him to the clinic* [a local health centre] *and check if I am not HIV-positive*." Another participant using metronidazole mentioned wanting to join the study to her husband, who forbade her to participate. However, she decided to join anyway: "*he did not know that I was using the study product, because he had refused me to join* [the]

For peer review only - http://bmjopen.bmj.com/site/about/guidelines.xhtml

study before… I used them [the study products] *without informing him.*" All sex workers except one stated that they had not discussed study participation with male clients.

Worries and concerns

In the FGDs, one woman reported hearing rumours prior to enrolling that vaginal products "*can damage the uterus or cause tumours in the womb.*" However, most participants thought that vaginal probiotics would be acceptable to Rwandan women. One GynLP user argued: "*They* [already] *give us vaginal pills*", by which she meant vaginal medications for yeast infections. Some women were concerned about future product availability and pricing. They hoped that probiotics would be distributed cheaply through the Rwandan *Mutuelle* public health insurance because they would otherwise be inaccessible to many women. One metronidazole user was concerned about a limited applicability of probiotics because BV is not diagnosed by laboratory testing in Rwanda: "*They do not have adequate medical instruments to test diseases, you tell the physician how* […] *you feel and by guessing the disease, he gives you at least four medications, saying that you may have trichomonas, you may have syphilis, you may have gonorrhoea* [she refers to syndromic management.[21,22]] *At health centre-level they do not have medical equipment to test diseases, meaning that they will not know who to give that* [probiotic/antibiotic maintenance therapy] *medication.*"

Vaginal practices and sexual risk-taking

At enrolment, 35/71 (49.3%) of the women reported to never use products inside the vagina, and at Month 6, this increased to 53/65 (81.5%) (OR 5.2, 95% CI 1.96-17.34; table 2). During FGDs, some women understood that vaginal washing practices may increase the risk of vaginal infection, but others did not. A participant stated: "*You get them* [i.e., vaginal diseases] *anyway… whether you wash or not*". In one FGD, 10 of 11 participants (90.9%) stated having ceased vaginal practices thanks to the study counselling. It should be noted that in contrast to many other African populations, Rwandan women use vaginal practices to increase rather than reduce vaginal lubrication. Women mentioned the use of herbs (*umushishiro*), Vaseline, and oils for this purpose. Self-reported sexual risk taking by face-to-face interview did not change over time, except for a significant reduction in reported numbers

For peer review only - http://bmjopen.bmj.com/site/about/guidelines.xhtml

of sex partners in the previous month at Month 6 compared to enrolment. No women in FGDs/IDIs mentioned adopting safer sex practices (such as consistent condom use) in response to the counselling messages. During face-to-face interviews at the Month 2 visit, 12 of 15 women (80%) who had an uncircumcised main sex partner reported asking him to regularly clean his penis in the future (online supplementary material table 2). While most women in FGDs understood that using condoms and improved penile hygiene could reduce BV rates (as shown in [6,16]), some mentioned that they found it difficult to discuss these topics with male partners. One participant stated that this is especially difficult being a sex worker: "*a man gives you his own money and you start educating him to wash!*" However, another sex worker reported refusing sex with uncircumcised clients: "*you leave him, because he has a lot* [of] *germs*". Several women reported discussing circumcision with their partners; one participant reported telling her husband: "*It is better that you do circumcision because it is a good thing… you would get a chance of not contracting diseases.*"

For peer review only

For peer review only - http://bmjopen.bmj.com/site/about/guidelines.xhtml

Table 2: Changes in reported vaginal cleansing practices and (sexual) behaviour between the enrolment and the M6 visit.

Self-reported sociodemographic characteristics	Enr (n=71)	M6 (n=65)	OR (95% CI)*	P value*
Reports using no products inside the vagina (other than for managing menses; all participants) n (%)	35 (49.3)	53 (81.5)	5.2 (1.96–17.34)	<0.001
Reports using no products inside the vagina (other than for managing menses; controls and metronidazole users only)† n (%)	15 (44.1)	27 (79.4)	13.0 (1.95–552.5)	0.002
Reports using water only n (%)	23 (32.4)	10 (15.4)	0.37 (0.13–0.92)	0.029
Reports using water and soap n (%)	3 (4.2)	2 (3.1)	0.67 (0.06–5.82)	1.00
Reports using paper, cloth or cotton wool n (%)	9 (12.7)	0 (0)	0.13 (0.00–0.93)‡	0.008
Reports using traditional herbs, stones, powders as vaginal cleansing practice n (%)	1 (1.4)	1 (1.5)	1.00 (0.01–78.5)‡	1.00
Mean weekly frequency of vaginal practices (95% CI)	2.15 (0.97–3.34)	0.64 (0.18–1.11)	NA	0.328
Median number of sex partners in last month at baseline or per month during follow-up period (IQR)	5 (3–16)	2 (1–4)	NA	<0.001
Any condom use reported in past two weeks (Enr) or since last study visit (M6), versus no condom use reported n (%)	64 (90.1)	60 (92.3)	1.67 (0.32–10.7)	0.727
Reports exchanging sex for money/goods in past month (Enr) or since last study visit (M6) n (%)	65 (91.5)	58 (89.2)	0.80 (0.16–3.72)	1.00

*McNemar's OR and p-value for binary variables and Wilcoxon signed-rank test p-value for continuous variables, comparing the response at M6 with the response at Enr. ORs with 95% CI were also calculated for binary pre-post data.
†N=34.
‡To enable calculation of effect measures, a zero value was replaced by 1.
CI, confidence interval; Enr, enrolment visit; IQR, inter-quartile range; M6, Month 6 visit; NA, not applicable; OR, odds ratio.

Correlates of adherence

In bivariable mixed effects models including the probiotic groups only, no participant characteristics were significantly associated with perfect adherence (table 3). However, non-significant trends were observed. Younger age (p=0.076), asking many questions at enrolment (compared to a few questions or no questions; structurally judged by a study nurse; p=0.116), having menses during the previous study interval (p=0.104), and reporting urogenital symptoms (p=0.103) were associated with a lower likelihood of perfect adherence. When including oral metronidazole users, menses was significantly associated with a lower likelihood of perfect adherence (p=0.008). There were no significant associations between randomisation group and perfect adherence.

For peer review only

Table 3: Participant characteristics associated with perfect adherence

Participant characteristics	EF+ and GynLP users		EF+, GynLP and oral metronidazole users	
	OR (95% CI)	P value	OR (95% CI)	P value
Randomisation group: GynLP versus EF+	0.68 (0.22–2.11)	0.505	ND	ND
Randomisation group:				
- EF+ versus metronidazole	ND	ND	0.53 (0.15–1.81)	0.308
- GynLP versus metronidazole			0.36 (0.11–1.23)	0.103
Age in years: ≥30 years versus <30	2.66 (0.90–7.82)	0.076	1.60 (0.61–4.15)	0.336
Marital status:				
- Married versus never married	0.97 (0.14–6.58)	0.976	1.17 (0.20–6.99)	0.865
- Divorced versus never married	1.18 (0.29–4.79)	0.912	1.39 (0.42–4.57)	0.586
- Widowed versus never married	ND	0.991	ND	0.990
At least some schooling versus no schooling	1.20 (0.59–2.45)	0.619	0.80 (0.22–2.95)	0.740
Number of sex partners last month: five or more versus four or less.	0.58 (0.18–1.83)	0.351	0.49 (0.17–1.37)	0.173
Exchanged sex for money/goods past month	ND	0.990	ND	0.986
Nurse reported participant asked questions at Enr				
- Yes, many versus none	0.19 (0.02–1.52)	0.116	0.15 (0.02–1.19)	0.072
- Yes, a few versus none	0.83 (0.24–2.83)	0.761	0.83 (0.27–2.57)	0.744
Had menses during study visit interval	0.41 (0.14–1.20)	0.104	0.26 (0.09–0.70)	0.008
Reported alcohol consumption during study:				
- Once or twice per week versus never	0.54 (0.14–2.12)	0.373	0.34 (0.11–1.08)	0.068
- More than twice per week versus never	0.92 (0.18–4.81)	0.920	0.81 (0.19–3.49)	0.774
Reported at least one urogenital symptom during study interval versus none	0.11 (0.01–1.56)	0.103	0.30 (0.04–2.16)	0.231
Reported at least one adverse event during study visit interval (excluding urogenital symptoms) versus none	0.43 (0.10–1.83)	0.253	0.55 (0.15–2.05)	0.371

Sociodemographic characteristics associated with perfect adherence in bivariable mixed effects models, in the enrolment–D7, D7–M1, and M1–M2 study visit intervals.
CI, confidence interval; D7, Day 7 visit; EF+, Ecologic Femi+; Enr, enrolment visit; GynLP, Gynophilus LP; M1, Month 1 visit; M2, Month 2 visit; ND, non-determinable; OR, odds ratio.

Vaginal infection knowledge

Almost all participants reported having heard of ‘diseases of the vagina’ and STIs before, but only 6/131 (4.6%) knew what bacteria were (table 4). The STIs most often spontaneously named (in numerical order) were HIV, gonorrhoea, and syphilis; only one participant reported having heard of BV. After having received an explanation about what BV is, only one of 131 woman reported ever having been diagnosed with BV. Most participants could name at least one cause or potential consequence of vaginal infections. Consequences wrongfully attributed to vaginal infections were cervical cancer/tumours (5/131; 3.8%), consequences to the infant such as being born with BV or congenital malformations (6/131; 4.6%), and death (4/131; 3.1%).

For peer review only

For peer review only - http://bmjopen.bmj.com/site/about/guidelines.xhtml

Table 4: Vaginal infection knowledge

	Recruitment (n=61)	Enrolment (n=70)	Total (n=131)
Median age (IQR)	32 (27–35)*	31 (27–35)	31 (27–35)
Has heard of diseases of the vagina before n (%)	60 (98.4)	70 (100)	130 (99.2)
Reports knowing what bacteria are before study n (%)	5 (8.2)	1 (1.4)	6 (4.6)
Reports having heard about STIs before study n (%)	61 (100)	70 (100)	131 (100)
If yes, spontaneously named, without probing† n (%)			
- HIV	58 (95.1)	65 (92.9)	123 (93.9)
- Gonorrhoea	58 (95.1)	65 (92.9)	123 (93.9)
- Syphilis	44 (72.1)	59 (84.3)	103 (78.7)
- Trichomoniasis	38 (62.3)	48 (68.6)	86 (65.7)
- Hepatitis	3 (4.9)	3 (4.3)	6 (4.6)
- Yeast infection	0	3 (4.3)	3 (2.3)
- BV	0	2 (2.9)	2 (1.5)
- Urinary tract infection	1 (1.6)	1 (1.4)	2 (1.5)
- Chlamydia	0	1 (1.4)	1 (0.8)
- Herpes	0	1 (1.4)	1 (0.8)
- HPV / cervical cancer	1 (1.6)	0	1 (0.8)
Reports having heard about BV before this study n (%)	1 (1.6)	0	1 (0.8)
Spontaneously reported reasons why women get vaginal disease, without probing† n (%)			
- Poor toilet hygiene	37 (60.7)	40 (57.1)	77 (58.8)
- Multiple sex partners	28 (45.9)	36 (51.4)	64 (48.9)
- After sex	25 (41.0)	30 (43.0)	55 (42.0)
- Dirty underwear	19 (31.2)	35 (50.0)	54 (41.2)
- Poor vaginal hygiene	26 (42.6)	22 (31.4)	48 (36.6)
- Poor penile hygiene of male partner(s)	4 (6.6)	17 (24.3)	21 (16.0)
- Traditional vaginal practices and washing	3 (4.9)	12 (17.1)	15 (11.5)
- New sex partner	6 (9.8)	3 (4.3)	9 (6.9)
- Use of contraception	1 (1.6)	3 (4.3)	4 (3.1)
- (Improper) use of sanitary pads or tampons	1 (1.6)	3 (4.3)	4 (3.1)
- Other	3 (4.9)‡	1 (1.4)§	4 (3.1)
- Cannot name any reasons	1 (1.6)	0	1 (0.8)
Spontaneously reported negative consequences of vaginal disease being named, without probing† n (%)			
- Foul smell from the vagina	30 (49.2)	39 (56.5)	69 (53.1)
- Difficulty getting pregnant	18 (29.5)	33 (47.8)	51 (39.2)
- Miscarriage	16 (26.2)	33 (47.8)	49 (37.7)
- Abnormal vaginal discharge	12 (19.7)	28 (40.6)	40 (30.8)
- Baby born too early	16 (26.2)	22 (31.9)	38 (29.2)
- Severe infection / fever of the woman	7 (11.5)	7 (10.1)	14 (10.8)
- Infection / fever of the newborn baby	5 (8.2)	3 (4.4)	8 (6.1)
- Itching	4 (6.6)	4 (5.8)	8 (6.1)
- Other consequences to the baby: being born with BV, congenital malformations, and others.	3 (4.9)	3 (4.4)	6 (4.6)
- Cervical cancer or tumours	2 (3.3)	3 (4.4)	5 (3.8)
- Death	4 (6.6)	0	4 (3.1)
- HIV/STIs	1 (1.6)	3 (4.4)	4 (3.1)
- Pain during intercourse	0	3 (4.4)	3 (2.3)
- Cannot name any consequence	17 (27.9)	19 (27.5)	36 (27.7)

*One missing value.
†Open-ended question. Totals may be more than 100%.
‡Participants report: "If you are infected with STIs", sharing underwear, and unprotected sex.
§Participant reports: vaginal medicine.
BV, bacterial vaginosis; HPV, human papilloma virus; IQR, interquartile range; STI, sexually transmitted infection.

For peer review only

For peer review only - http://bmjopen.bmj.com/site/about/guidelines.xhtml

DISCUSSION

Several studies of different vaginal probiotics have been conducted, some of them in sub-Saharan Africa.[10–13] However, none reported in-depth acceptability and adherence data. Our study suggests high vaginal probiotic acceptability and adherence in high-risk Rwandan women. We found no statistically significant correlates of perfect adherence, partially due to limited statistical power, but younger age, asking many questions about product use at enrolment, current menses, and reporting urogenital symptoms showed trends towards a lower likelihood of perfect adherence. Vaginal probiotics are currently unavailable on the market in most African countries, and it is important to study acceptability in different target populations to inform product development and future marketing strategies. Adherence to metronidazole was comparable to, or slightly higher than, adherence reported in previously conducted studies.[19,23]

We could not evaluate the impact of self-reported acceptability aspects on adherence because almost all women reported very high acceptability in face-to-face interviews throughout the trial. Such interviews are known to suffer from social desirability bias. However, women seemed to speak freely in the FGDs, and those data indicate that they did not have major issues with product attributes or insertion. However, some women reported difficulties due to lack of male partner support. The reported increase in vaginal wetness after probiotic insertion was not considered problematic, as lubrication during sex is preferred by most Rwandan men and women.[24] This might be different in other countries where dry sex is preferred.[25] We did find a non-significant lower adherence to GynLP compared to EF+, which might be explained by differences in formulation: GynLP forms a gel in the vagina whereas EF+ capsules merely release lyophilised bacteria. Previous research indicated high adherence to GynLP.[26] Unfortunately, the impact of these formulation differences was insufficiently probed during the FGDs; the impact of product formulation on acceptability and adherence should be investigated in future clinical trials. Participants indicated that they found the diary cards helpful in reminding them to use their products, and we believe that self-monitoring tools might indeed be helpful in maximising adherence and therefore recommend them for use in future studies.[27]

For peer review only - http://bmjopen.bmj.com/site/about/guidelines.xhtml

Our data suggest that counselling was partially effective in changing behaviours that increase BV risk. While these results are encouraging, it is difficult to assess to what extent they were influenced by social desirability bias. Significantly more women reported not engaging in vaginal practices at the end of the study, and most women with uncircumcised steady male partners reported having discussed penile hygiene with them. However, many women mentioned in FGDs that they found it difficult to discuss condom use and penile hygiene with male partners, especially clients. Women reduced their sexual risks only to a limited extent during follow-up, reporting a reduction in numbers of sex partners but no differences in engaging in sex work and condom use in face-to-face interviews. We did not ask women to what extent they depended on sex work for subsistence. Women who only partially depend on sex work may find it easier to negotiate with male partners.

Two probiotics-related themes that emerged from the stakeholders consultations that had not been raised by the study participants were uncertainty about long-term side effects (women in the pilot trial used the products for only two months) and whether probiotic bacteria (in this case lactobacilli) could also be delivered orally instead of vaginally. We have since conducted a systematic review, which showed that long-term safety of vaginal probiotics has not yet been evaluated.[28]

Our survey with women at recruitment sessions and enrolment visits showed that high-risk Rwandan women had heard of several STIs, but were generally unaware of BV, its causes and potential consequences, and what they can do to prevent it. Experiences with HIV show that public health interventions can only succeed if health care professionals and the public have sufficient knowledge of causes and consequences of disease.[29–31] High-risk Rwandan women (and health care professionals) should therefore be educated about BV, and vaginal probiotics studies should include counselling for all participants on vaginal diseases and how to prevent them.

Limitations

Our study had limited statistical power, and social desirability bias may have affected some of our results, as is often the case in studies of this nature. Additionally, it should be noted that product

For peer review only - http://bmjopen.bmj.com/site/about/guidelines.xhtml

efficacy, availability and cost are important determinants of acceptability, and were not evaluated in our study, although preliminary efficacy results in this study were promising.[17] We could not directly compare experiences with, and opinions about, the two different vaginal probiotics because each woman used only one product and qualitative data depth was suboptimal. In the FGDs/IDIs, it was sometimes difficult to ascertain whether participants were referring to personal experiences, or to wider community perceptions. Strengths of our study include the use of a mixed-methods approach and triangulated adherence data.

CONCLUSIONS

The prevention of BV recurrence will likely have to include several components to be successful, such as improved diagnostics, treatments, and prophylactic products (for example probiotics), but also improved information, education, and counselling messages targeted to at-risk women and their partners. The results of this study can be used to inform future product development, and to fine-tune counselling messages in future trials.

For peer review only

For peer review only - http://bmjopen.bmj.com/site/about/guidelines.xhtml

Acknowledgments We thank the study participants, the Rinda Ubuzima team and other colleagues in Rwanda and the United Kingdom who contributed, the funders of this study, as well as the Trial Steering Committee for trial oversight. We thank Biose and Winclove for the donation of study products.

Author contributions JvdW obtained the research funding and wrote the study protocol and data collection documents. AN, EL, SA, and JvdW were members of the Trial Steering Committee. SA, MU, MMU, and JvdW collected the primary data. MU and MMU performed the FGDs and IDIs. MV and JvdW developed the analytical approach and performed the statistical analyses. MV and JvdW wrote the manuscript. All authors commented on and approved the final manuscript.

Funding This work was funded by the DFID/MRC/Wellcome Trust Joint Global Health Trials Scheme as a Development Project (grant reference MR/M017443/1; grant title: "Preparing for a clinical trial of interventions to maintain normal vaginal microbiota for preventing adverse reproductive health outcomes in Africa"). Vaginal probiotics for use in the trial were donated free of charge by Winclove Probiotics (Amsterdam, The Netherlands) and Biose (formerly Probionov; Aurillac, France). The findings and conclusions in this paper are those of the authors and do not necessarily represent the views of the authors' institutions or companies, or the funder. None of the authors were paid to write this article. The corresponding author had full access to the data and had final responsibility for the decision to submit for publication.

Competing interests AN is employed by Biose (owner of trial product GynLP) and EL by Winclove Probiotics BV (owner of trial product EF+). AN has financial and/or intellectual investments in competing products. The other authors report no competing interests.

Patient consent All participants provided written informed consent for study participation, and separate written informed consent for participation in FGDs/IDIs.

For peer review only - http://bmjopen.bmj.com/site/about/guidelines.xhtml

Ethics approval The study was sponsored by the University of Liverpool, approved by the Rwanda National Ethics Committee and the University of Liverpool Research Ethics Subcommittee for Physical Interventions, and registered on ClinicalTrials.gov (NCT02459665).

Data sharing statement The data supporting the findings of this publication are retained by the corresponding author (JvdW) and will not be made openly accessible due to privacy concerns. Fully anonymised data can be made available by written request to j.vandewijgert@liverpool.ac.uk after assurance that the intended data usage is compliant with relevant ethical approvals and privacy will be maintained.

For peer review only

For peer review only - http://bmjopen.bmj.com/site/about/guidelines.xhtml

Figure footnotes

Figure 1: Flowchart of the study

*Totals to 110 reasons among 102 women because there could be more than one reason per woman.
†Reasons: outside of metronidazole treatment window (n=5), enrolment target already met (n=4), has a mental disorder (n=1), did not complete screening procedures and was subsequently lost to follow=up (n=1), withdrew consent during the screening visit because she thought the reimbursement was too low (n=1).
‡Reasons: moved away from Kigali (n=2), lost interest because symptoms resolved (n=1), and was verbally harassed by partner and sister about study participation (n=1).

Acceptability assessments were made at enrolment and at the M2 visit. Adherence assessments were made using self-rated assessments, pictorial diary cards, and returned packaging at the D7, M1, and M2 visits (after which product use was ceased). The vaginal infection knowledge survey was held at recruitment sessions in the community and at the enrolment visit. Changes in sexual risk-taking and vaginal practices were assessed at each follow-up visits and compared to answers given during the enrolment visit. All of these themes were discussed during the eight FGDs and IDIs.

BV, bacterial vaginosis; D7, day 7 visit; FGD, focus group discussion; IDI, in-depth interview; M1/2/6, month 1/2/6 visit; RU, Rinda Ubuzima; TV, *Trichomonas vaginalis*.

For peer review only

For peer review only - http://bmjopen.bmj.com/site/about/guidelines.xhtml

References

1 van de Wijgert JHHM, Borgdorff H, Verhelst R, *et al.* The vaginal microbiota: what have we learned after a decade of molecular characterization? *PLOS ONE* 2014;**9**:e105998. doi:10.1371/journal.pone.0105998

2 van de Wijgert JHHM, Morrison CS, Cornelisse PGA, *et al.* Bacterial vaginosis and vaginal yeast, but not vaginal cleansing, increase HIV-1 acquisition in African women. *J Acquir Immune Defic Syndr 1999* 2008;**48**:203–10. doi:10.1097/QAI.0b013e3181743936

3 van de Wijgert JHHM. The vaginal microbiome and sexually transmitted infections are interlinked: consequences for treatment and prevention. *PLOS Med* 2017;**14**:e1002478. doi:10.1371/journal.pmed.1002478

4 Li J, McCormick J, Bocking A, *et al.* Importance of vaginal microbes in reproductive health. *Reprod Sci* 2012;**19**:235–42. doi:10.1177/1933719111418379

5 Nelson DB, Hanlon AL, Wu G, *et al.* First trimester levels of BV-associated bacteria and risk of miscarriage among women early in pregnancy. *Matern Child Health J* 2015;**19**:2682–7. doi:10.1007/s10995-015-1790-2

6 Hay P. Recurrent bacterial vaginosis. *Curr Opin Infect Dis* 2009;**22**:82–6. doi:10.1097/QCO.0b013e32832180c6

7 Verstraelen H, Verhelst R. Bacterial vaginosis: an update on diagnosis and treatment. *Expert Rev Anti Infect Ther* 2009;**7**:1109–24. doi:10.1586/eri.09.87

8 Torrone EA, Morrison CS, Chen P-L, *et al.* Prevalence of sexually transmitted infections and bacterial vaginosis among women in sub-Saharan Africa: An individual participant data meta-analysis of 18 HIV prevention studies. *PLOS Med* 2018;**15**:e1002511. doi:10.1371/journal.pmed.1002511

9 Bradshaw CS, Brotman RM. Making inroads into improving treatment of bacterial vaginosis – striving for long-term cure. *BMC Infect Dis* 2015;**15**:292. doi:10.1186/s12879-015-1027-4

10 Anukam KC, Osazuwa E, Osemene GI, *et al.* Clinical study comparing probiotic *Lactobacillus* GR-1 and RC-14 with metronidazole vaginal gel to treat symptomatic bacterial vaginosis. *Microbes Infect* 2006;**8**:2772–6. doi:10.1016/j.micinf.2006.08.008

11 Ling Z, Liu X, Chen W, *et al.* The restoration of the vaginal microbiota after treatment for bacterial vaginosis with metronidazole or probiotics. *Microb Ecol* 2013;**65**:773–80. doi:10.1007/s00248-012-0154-3

12 Ngugi BM, Hemmerling A, Bukusi EA, *et al.* Effects of bacterial vaginosis-associated bacteria and sexual intercourse on vaginal colonization with the probiotic *Lactobacillus crispatus* CTV-05. *Sex Transm Dis* 2011;**38**:1020–7. doi:10.1097/OLQ.0b013e3182267ac4

13 Bradshaw CS, Pirotta M, De Guingand D, *et al.* Efficacy of oral metronidazole with vaginal clindamycin or vaginal probiotic for bacterial vaginosis: randomised placebo-

For peer review only

For peer review only - http://bmjopen.bmj.com/site/about/guidelines.xhtml

controlled double-blind trial. *PLOS ONE* 2012;**7**:e34540. doi:10.1371/journal.pone.0034540

14 van der Straten A, Montgomery ET, Cheng H, *et al.* High acceptability of a vaginal ring intended as a microbicide delivery method for HIV prevention in African women. *AIDS Behav* 2012;**16**:1775–86. doi:10.1007/s10461-012-0215-0

15 Merkatz RB, Plagianos M, Hoskin E, *et al.* Acceptability of the nestorone®/ethinyl estradiol contraceptive vaginal ring: development of a model; implications for introduction. *Contraception* 2014;**90**:514–21. doi:10.1016/j.contraception.2014.05.015

16 Liu CM, Hungate BA, Tobian AAR, *et al.* Penile microbiota and female partner bacterial vaginosis in Rakai, Uganda. *mBio* 2015;**6**:e00589. doi:10.1128/mBio.00589-15

17 van de Wijgert J, Verwijs MC, Agaba SK, *et al.* Intermittent lactobacilli-containing vaginal probiotic or metronidazole use to prevent bacterial vaginosis recurrence: safety and preliminary efficacy by microscopy and sequencing. *MedRxiv* Published Online First: 8 July 2019. doi:10.1101/19001156

18 Sobel JD, Ferris D, Schwebke J, *et al.* Suppressive antibacterial therapy with 0.75% metronidazole vaginal gel to prevent recurrent bacterial vaginosis. *Am J Obstet Gynecol* 2006;**194**:1283–9. doi:10.1016/j.ajog.2005.11.041

19 McClelland RS, Richardson BA, Hassan WM, *et al.* Improvement of vaginal health for Kenyan women at risk for acquisition of Human Immunodeficiency Virus type 1: results of a randomized trial. *J Infect Dis* 2008;**197**:1361–8. doi:10.1086/587490

20 Kestelyn E, van Nuil JI, Umulisa MM, *et al.* High acceptability of a contraceptive vaginal ring among women in Kigali, Rwanda. *PLOS ONE* 2018;**13**:e0199096. doi:10.1371/journal.pone.0199096

21 World Health Organization. Guidelines for the management of sexually transmitted infections. 2003.http://applications.emro.who.int/aiecf/web79.pdf (accessed 12 Mar 2019).

22 Binagwaho A, editor. *National guidelines for prevention and management of HIV, STIs & other blood borne infections*. Republic of Rwanda - Ministry of Health 2013. https://aidsfree.usaid.gov/sites/default/files/hts_policy_rwanda.pdf (accessed 12 Mar 2019).

23 McClelland RS, Balkus JE, Lee J, *et al.* Randomized trial of periodic presumptive treatment with high-dose intravaginal metronidazole and miconazole to prevent vaginal infections in HIV-negative women. *J Infect Dis* 2015;**211**:1875–82. doi:10.1093/infdis/jiu818

24 Veldhuijzen N, Nyinawabega J, Umulisa M, *et al.* Preparing for microbicide trials in Rwanda: focus group discussions with Rwandan women and men. *Cult Health Sex* 2006;**8**:395–406. doi:10.1080/13691050600859302

25 Low N, Chersich MF, Schmidlin K, *et al.* Intravaginal practices, bacterial vaginosis, and HIV infection in women: individual participant data meta-analysis. *PLOS Med* 2011;**8**:e1000416. doi:10.1371/journal.pmed.1000416

For peer review only

For peer review only - http://bmjopen.bmj.com/site/about/guidelines.xhtml

26 Dausset C, Patrier S, Gajer P, *et al.* Comparative phase I randomized open-label pilot clinical trial of Gynophilus® (Lcr regenerans®) immediate release capsules versus slow release muco-adhesive tablets. *Eur J Clin Microbiol Infect Dis* 2018;**37**:1869–80. doi:10.1007/s10096-018-3321-8

27 Safren SA, W. Otto M, Worth JL, *et al.* Two strategies to increase adherence to HIV antiretroviral medication: Life-Steps and medication monitoring. *Behav Res Ther* 2001;**39**:1151–62. doi:10.1016/S0005-7967(00)00091-7

28 van de Wijgert JHHM, Verwijs MC. Lactobacilli-containing vaginal probiotics to cure or prevent bacterial or fungal vaginal dysbiosis: a systematic review and recommendations for future trial designs. *BJOG*;**0**. doi:10.1111/1471-0528.15870

29 Glick P, Sahn DE. Changes in HIV/AIDS knowledge and testing behavior in Africa: how much and for whom? *J Popul Econ* 2007;**20**:383–422. doi:10.1007/s00148-006-0085-8

30 Peltzer K, Matseke G, Mzolo T, *et al.* Determinants of knowledge of HIV status in South Africa: results from a population-based HIV survey. *BMC Public Health* 2009;**9**:174. doi:10.1186/1471-2458-9-174

31 Fay H, Baral SD, Trapence G, *et al.* Stigma, health care access, and HIV knowledge among men who have sex with men in Malawi, Namibia, and Botswana. *AIDS Behav* 2011;**15**:1088–97. doi:10.1007/s10461-010-9861-2

For peer review only

For peer review only - http://bmjopen.bmj.com/site/about/guidelines.xhtml

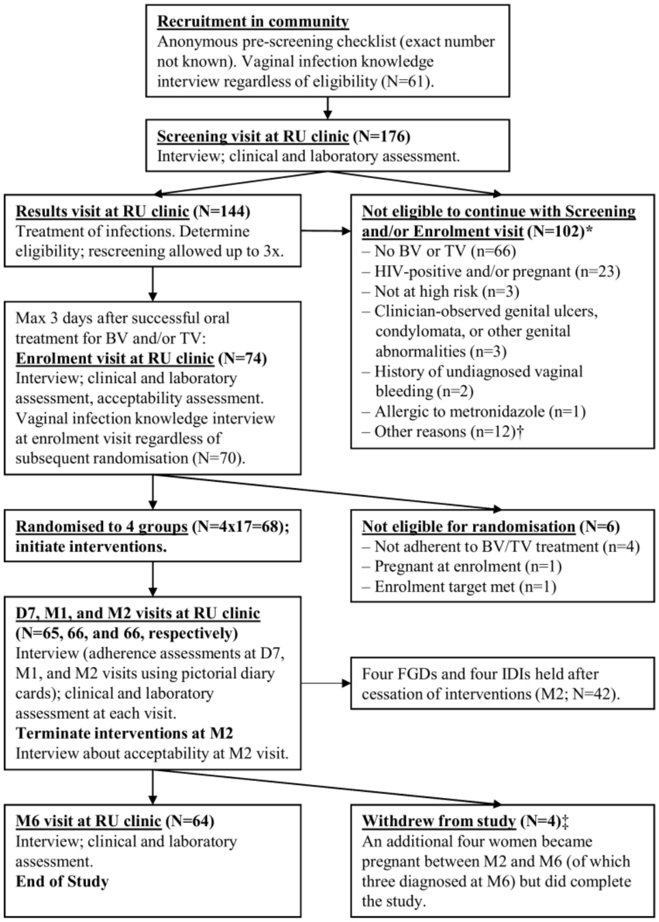

Figure 1: Flowchart of the study
*Totals to 110 reasons among 102 women because there could be more than one reason per woman.
†Reasons: outside of metronidazole treatment window (n=5), enrolment target already met (n=4), has a mental disorder (n=1), did not complete screening procedures and was subsequently lost to follow=up (n=1), withdrew consent during the screening visit because she thought the reimbursement was too low (n=1).
‡Reasons: moved away from Kigali (n=2), lost interest because symptoms resolved (n=1), and was verbally harassed by partner and sister about study participation (n=1).

Acceptability assessments were made at enrolment and at the M2 visit. Adherence assessments were made using self-rated assessments, pictorial diary cards, and returned packaging at the D7, M1, and M2 visits (after which product use was ceased). The vaginal infection knowledge survey was held at recruitment sessions in the community and at the enrolment visit. Changes in sexual risk-taking and vaginal practices were assessed at each follow-up visits and compared to answers given during the enrol visit. All this themes

were discussed during the eight FGDs and IDIs.

BV, bacterial vaginosis; D7, day 7 visit; FGD, focus group discussion; IDI, in-depth interview; M1/2/6, month 1/2/6 visit; RU, Rinda Ubuzima; TV, *Trichomonas vaginalis*.

215x279mm (300 x 300 DPI)

Supplementary Figure 1: Pictorial diary card

Date/Month	Descriptions	Monday	Tuesday	Wednesday	Thursday	Friday	Saturday	Sunday
Indicate each time you used study product								
	Used study product							
Indicate each sex act								
	Sex with condom							
	Sex without condom							
Indicate each time you washed/inserted something inside the vagina other than study product								
By washing inside, we mean inserting an entire finger inside the vaginal canal								
	Washed inside vagina with water only							
	Washed inside vagina with soap and water							
	Inserted something else (herbs, powders, etc.)							
Indicate each day of menstrual bleeding								
	Had menstrual bleeding							

The picture provided is the English translation of the pictorial card; participants received a version in Kinyarwanda.

For peer review only

Supplementary Table 1: Baseline characteristics of enrolled population

	Controls (n=17)	Metronidazole (n=17)	EF+ (n=17)	GynLP (n=17)
Median age (IQR)	29 (24–36)	30 (27–34)	33 (28–35)	30 (27–35)
Marital status n (%)				
- Never married	16 (94.1)	11 (64.7)	10 (58.8)	13 (76.5)
- Married	1 (5.9)	1 (5.9)	2 (11.8)	1 (5.9)
- Divorced	0	5 (29.4)	4 (23.5)	3 (17.6)
- Widowed	0	0	1 (5.9)	0
Education level n (%)				
- No schooling	5 (29.4)	3 (17.6)	3 (17.6)	3 (17.7)
- Primary school not completed	7 (41.2)	7 (41.2)	13 (76.5)	4 (23.5)
- Primary school completed	4 (23.5)	5 (29.4)	1 (5.9)	7 (41.2)
- At least some secondary school	1 (5.9)	2 (11.8)	0	3 (17.7)
Median number of sex partners last month (IQR)	5 (3–20)	5 (2–10)	3 (2–15)	3 (2–20)
Exchanged sex for money/goods past month n (%)	17 (100)	14 (82.4)	15 (88.2)	17 (100)
At least one laboratory-confirmed STI* n (%)	8 (47.1)	8 (47.1)	4 (23.5)	9 (52.9)
Median weekly frequency of washing body (IQR)	7 (7–7)	7 (7–7)	7 (7–7)	7 (4–7)
Ever washing the genitalia n (%)				
- Yes, outside only	12 (70.7)	14 (82.4)	15 (88.3)	14 (82.3)
- Yes, both inside and outside	5 (29.4)	3 (17.6)	2 (11.7)	3 (17.7)
- Yes, inside only	0	0	0	0
If reports washing inside, median weekly frequency (IQR)	14 (7–16)	14 (14–14)	11 (7–14)	7 (3–12)

*Chlamydia, gonorrhoea, and/or syphilis.
EF+, Ecologic Femi+; Enr, enrolment visit; GynLP, Gynophilus LP; IQR, inter-quartile range; M2, Month 2 visit; STI, sexually transmitted disease.

For peer review only

Supplementary Table 2: Acceptability of interventions

Acceptability of study products at Enr	Controls (n=17)	Metronidazole (n=17)	EF+ (n=17)	GynLP (n=17)
Nurse reports having explained intervention to participant in detail n (%)	17 (100)	17 (100)	17 (100)	17 (100)
Nurse reports participant asked questions n (%)*				
- Yes, a few	6 (35.3)	2 (11.8)	11 (64.7)	11 (64.7)
- Yes, many	0	0	0	2 (11.8)
First dose applied† under supervision n (%)	NA	17 (100)	17 (100)	17 (100)
Median number of attempts participant made until successful application (IQR)	NA	NA	1 (1–1)	1 (1–1)
Participant seemed comfortable with the insertion after these attempts, according to study nurse n (%)	NA	NA		
- Yes, very			17 (100)	16 (94.1)
- Yes, somewhat			0	1 (5.9)
**Acceptability of study products at M2**				
Self-reported usual time of insertion n (%)				
- Before going to sleep	NA	NA	17 (100)	15 (100)‡
- After bathing in the morning			0	0
Level of comfort with vaginal insertion after 2 months of use, self-reported n (%)	NA	NA		
- Very comfortable			17 (100)	15 (100)‡
- Somewhat comfortable			0	0
Reported insertion becoming easier over time n (%)	NA	NA	17 (100)	15 (100)‡
Reported manner of insertion§ n (%)				
- While lying down	NA	NA	17 (100)	14 (93.3)‡
- While squatting			1 (5.9)	1 (6.7)
**Acceptability of penile hygiene intervention at M2**				
Reports having told main sex partner to regularly clean the penis, including underneath the foreskin n (%)¶				
- Yes	3 (17.7)	3 (18.8)	3 (17.6)	3 (18.8)
- No, because he is circumcised	10 (58.8)	9 (56.2)	6 (35.3)	5 (31.3)
- No, other reason	1 (5.9)	0	1 (5.9)	1 (6.3)
If yes, response by the main partner n (%)‖				
- He said that he would do so in the future	2 (66.7)	1 (33.3)	1 (33.3)	1 (33.3)
- He said that he already does this	1 (33.3)	1 (33.3)	0	1 (33.3)
- He said that he is not interested	0	1 (33.3)	2 (66.7)	1 (33.3)

*One missing value.
†Whether oral insertion (oral metronidazole group) or vaginal insertion (Ecologic Femi+ and Gynophilus LP groups).
‡N=15 due to participants withdrawing informed consent.
§Multiple answers possible; hence totals can be more than 100%.
¶Women with no main sex partner not included.
‖N=3 in all four groups.
EF+, Ecologic Femi+; Enr, enrolment visit; GynLP, Gynophilus LP; IQR, inter-quartile range; M2, Month 2 visit; NA, Not applicable.

For peer review only

STROBE Statement—Checklist of items that should be included in reports of ***cohort studies***

	Item No	Recommendation	Reported on page #
**Title and abstract**	1	(*a*) Indicate the study's design with a commonly used term in the title or the abstract	1
		(*b*) Provide in the abstract an informative and balanced summary of what was done and what was found	2
**Introduction**			
Background/rationale	2	Explain the scientific background and rationale for the investigation being reported	4
Objectives	3	State specific objectives, including any prespecified hypotheses	4
**Methods**			
Study design	4	Present key elements of study design early in the paper	4
Setting	5	Describe the setting, locations, and relevant dates, including periods of recruitment, exposure, follow-up, and data collection	4
Participants	6	(*a*) Give the eligibility criteria, and the sources and methods of selection of participants. Describe methods of follow-up	4,5
		(*b*) For matched studies, give matching criteria and number of exposed and unexposed	NA
Variables	7	Clearly define all outcomes, exposures, predictors, potential confounders, and effect modifiers. Give diagnostic criteria, if applicable	6
Data sources/ measurement	8*	For each variable of interest, give sources of data and details of methods of assessment (measurement). Describe comparability of assessment methods if there is more than one group	6
Bias	9	Describe any efforts to address potential sources of bias	6
Study size	10	Explain how the study size was arrived at	NA (Primary outcomes paper)
Quantitative variables	11	Explain how quantitative variables were handled in the analyses. If applicable, describe which groupings were chosen and why	6
Statistical methods	12	(*a*) Describe all statistical methods, including those used to control for confounding	6,7
		(*b*) Describe any methods used to examine subgroups and interactions	6,7
		(*c*) Explain how missing data were addressed	NA (indicated in tables if missing)
		(*d*) If applicable, explain how loss to follow-up was addressed	NA
		(*e*) Describe any sensitivity analyses	NA
**Results**			
Participants	13*	(a) Report numbers of individuals at each stage of study—eg numbers potentially eligible, examined for eligibility, confirmed eligible, included in the study, completing follow-up, and analysed	Page 8, Figure 1
		(b) Give reasons for non-participation at each stage	8, Figure 1
		(c) Consider use of a flow diagram	Figure 1
Descriptive data	14*	(a) Give characteristics of study participants (eg demographic, clinical, social) and information on exposures and potential confounders	8
		(b) Indicate number of participants with missing data for each variable of	8 (missing

For peer review only - http://bmjopen.bmj.com/site/about/guidelines.xhtml

		interest	data in footnotes in table, if applicable)
		(c) Summarise follow-up time (eg, average and total amount)	8
Outcome data	15*	Report numbers of outcome events or summary measures over time	9
Main results	16	(*a*) Give unadjusted estimates and, if applicable, confounder-adjusted estimates and their precision (eg, 95% confidence interval). Make clear which confounders were adjusted for and why they were included	9
		(*b*) Report category boundaries when continuous variables were categorized	Table 1
		(*c*) If relevant, consider translating estimates of relative risk into absolute risk for a meaningful time period	NA
Other analyses	17	Report other analyses done—eg analyses of subgroups and interactions, and sensitivity analyses	Page 12 and beyond
**Discussion**			
Key results	18	Summarise key results with reference to study objectives	Page 18
Limitations	19	Discuss limitations of the study, taking into account sources of potential bias or imprecision. Discuss both direction and magnitude of any potential bias	Page 19
Interpretation	20	Give a cautious overall interpretation of results considering objectives, limitations, multiplicity of analyses, results from similar studies, and other relevant evidence	Page 18, 19
Generalisability	21	Discuss the generalisability (external validity) of the study results	Page 18, 19
**Other information**			
Funding	22	Give the source of funding and the role of the funders for the present study and, if applicable, for the original study on which the present article is based	Page 21

*Give information separately for exposed and unexposed groups.

Note: An Explanation and Elaboration article discusses each checklist item and gives methodological background and published examples of transparent reporting. The STROBE checklist is best used in conjunction with this article (freely available on the Web sites of PLoS Medicine at http://www.plosmedicine.org/, Annals of Internal Medicine at http://www.annals.org/, and Epidemiology at http://www.epidem.com/). Information on the STROBE Initiative is available at http://www.strobe-statement.org.

For peer review only - http://bmjopen.bmj.com/site/about/guidelines.xhtml

Vaginal Probiotic Adherence and Acceptability in Rwandan Women with High Sexual Risk Participating in a Pilot Randomised Controlled Trial: A Mixed-Methods Approach

Journal:	*BMJ Open*
Manuscript ID	bmjopen-2019-031819.R2
Article Type:	Original research
Date Submitted by the Author:	04-Mar-2020
Complete List of Authors:	Verwijs, Marijn; Institute of Infection and Global Health, University of Liverpool,<br>Agaba, Stephen; Rinda Ubuzima<br>Umulisa, Marie ; Rinda Ubuzima<br>Uwineza, Mireille; Rinda Ubuzima<br>Nivoliez, Adrien; Biose,<br>Lievens, Elke; Winclove<br>van de Wijgert, Janneke H.H.M.; University of Liverpool, Institute of Infection and Global Health; Universitair Medisch Centrum Utrecht, Julius Center for Health Sciences and Primary Care
**Primary Subject Heading**:	Sexual health
Secondary Subject Heading:	Sexual health, Obstetrics and gynaecology, Public health, Qualitative research
Keywords:	bacterial vaginosis, vaginal probiotic, adherence, acceptability, Africa

For peer review only - http://bmjopen.bmj.com/site/about/guidelines.xhtml

I, the Submitting Author has the right to grant and does grant on behalf of all authors of the Work (as defined in the below author licence), an exclusive licence and/or a non-exclusive licence for contributions from authors who are: i) UK Crown employees; ii) where BMJ has agreed a CC-BY licence shall apply, and/or iii) in accordance with the terms applicable for US Federal Government officers or employees acting as part of their official duties; on a worldwide, perpetual, irrevocable, royalty-free basis to BMJ Publishing Group Ltd ("BMJ") its licensees and where the relevant Journal is co-owned by BMJ to the co-owners of the Journal, to publish the Work in this journal and any other BMJ products and to exploit all rights, as set out in our licence.

The Submitting Author accepts and understands that any supply made under these terms is made by BMJ to the Submitting Author unless you are acting as an employee on behalf of your employer or a postgraduate student of an affiliated institution which is paying any applicable article publishing charge ("APC") for Open Access articles. Where the Submitting Author wishes to make the Work available on an Open Access basis (and intends to pay the relevant APC), the terms of reuse of such Open Access shall be governed by a Creative Commons licence – details of these licences and which Creative Commons licence will apply to this Work are set out in our licence referred to above.

Other than as permitted in any relevant BMJ Author's Self Archiving Policies, I confirm this Work has not been accepted for publication elsewhere, is not being considered for publication elsewhere and does not duplicate material already published. I confirm all authors consent to publication of this Work and authorise the granting of this licence.

For peer review only

Vaginal Probiotic Adherence and Acceptability in Rwandan Women with High Sexual Risk Participating in a Pilot Randomised Controlled Trial: A Mixed-Methods Approach

Authors: Marijn C. Verwijs (MD)[1], Stephen K. Agaba (MD)[2], Marie-Michele Umulisa (MA)[2], Mireille Uwineza (BA)[2], Adrien Nivoliez (PhD)[3], Elke Lievens (PhD)[4], Janneke H.H.M. van de Wijgert (MD PhD MPH)[1,5].

Author affiliations

[1]Institute of Infection and Global Health, University of Liverpool, Liverpool, United Kingdom.

[2]Rinda Ubuzima, Kigali, Rwanda.

[3]Biose, Aurillac, France.

[4]Winclove Probiotics, Amsterdam, The Netherlands.

[5]Julius Center for Health Sciences and Primary Care, University Medical Center Utrecht, Utrecht University, Utrecht, The Netherlands.

Correspondence to

Professor Janneke van de Wijgert, MD PhD MPH

Department of Clinical Infection, Microbiology and Immunology

Institute of Infection and Global Health, University of Liverpool

Ronald Ross Building, 8 West Derby Street, Liverpool L69 7BE, United Kingdom

Email: j.vandewijgert@liverpool.ac.uk

ORCID: 0000-0003-2728-4560

Word counts:

Word count abstract: 300 words (max. 300 words).

Word count main text: 3,934 words (max. 4000 words).

Number of tables and figures: 5 (max. 5).

Number of supplementary tables and figures: 3.

For peer review only - http://bmjopen.bmj.com/site/about/guidelines.xhtml

ABSTRACT

Objectives To evaluate adherence and acceptability of intermittent vaginal probiotic or antibiotic use to prevent bacterial vaginosis (BV) recurrence.

Design Repeated adherence and acceptability assessments using mixed methods within a pilot randomised controlled trial.

Setting Research clinic in Kigali, Rwanda.

Participants Rwandan women with high sexual risk.

Interventions Women diagnosed with BV and/or trichomoniasis were randomised to four groups (n=17 each) after completing metronidazole treatment: behavioural counselling only, or behavioural counselling plus two-month intermittent use of oral metronidazole, Ecologic Femi+ (EF+) vaginal capsule, or Gynophilus LP (GynLP) vaginal tablet.

Outcome measures Adherence and acceptability were assessed by structured face-to-face interviews, semi-structured focus group discussions and in-depth interviews, daily diaries, and counting of used/unused study products in randomised women (n=68). Vaginal infection knowledge was assessed by structured face-to-face interviews in randomised women and women attending recruitment sessions (n=131).

Results Most women (93%) were sex workers, 99.2% were unfamiliar with BV, and none had ever used probiotics. All probiotic users (n=32) reported that insertion became easier over time. Triangulated adherence data showed that 17/17 EF+ users and 13/16 GynLP users used ≥80% of required doses (Fisher's exact p=0.103). Younger age (p=0.076), asking many questions at enrolment (p=0.116), having menses (p=0.104), and reporting urogenital symptoms (p=0.103) were non-significantly associated with lower perfect adherence. Women believed that the probiotics reduced BV recurrence, but reported that partners were sometimes unsupportive of study participation. Self-reported vaginal washing practices decreased during follow-up, but sexual risk behaviours did not. Most women (12/15) with an uncircumcised steady partner discussed penile hygiene with him, but many women found this difficult, especially with male clients.

For peer review only - http://bmjopen.bmj.com/site/about/guidelines.xhtml

Conclusions High-risk women require education about vaginal infections. Vaginal probiotic acceptability and adherence were high in this cohort. Our results can be used to inform future product development and to fine-tune counselling messages in prevention programs.

Trial registration ClinicalTrials.gov (NCT02459665).

Keywords (5) bacterial vaginosis, vaginal probiotic, adherence, acceptability, Africa.

ARTICLE SUMMARY

Strengths and limitations of this study

- We conducted this research in the context of a pilot randomised controlled trial, and statistical power was therefore limited.
- We triangulated different sources of adherence data to maximise accuracy, and used a mixed-methods approach to evaluate acceptability.
- We could not directly compare experiences with, and opinions about, the two different vaginal probiotics because each woman used only one product and qualitative data depth was suboptimal.
- Social desirability bias may have affected some of the results.
- The results of this study may not be generalizable to women at lower risk of sexually transmitted or urogenital infections.

For peer review only

For peer review only - http://bmjopen.bmj.com/site/about/guidelines.xhtml

INTRODUCTION

Bacterial vaginosis (BV) is a vaginal condition in which fastidious anaerobes such as *Gardnerella vaginalis* increase while beneficial, lactic acid-producing lactobacilli decrease.[1] Often asymptomatic, it is associated with increased risks of sexually transmitted infections (STIs) and HIV acquisition, pelvic inflammatory disease, and adverse pregnancy outcomes.[2–5] Although BV is treatable with antibiotics, the risk of recurrence is high.[6,7] The prevalence of BV varies among regions and ethnic groups but is highest in sub-Saharan Africa, where it is estimated at 30-50%.[8]

Vaginally-administered probiotics containing lactobacilli are considered a promising new strategy to restore a lactobacilli-dominated vaginal microbiota during and/or after antibiotic treatment, or to prevent BV.[9] While some probiotics have been available on the market for several years, clinical trials to support beneficial effects have only recently been initiated for most products.[10–13] Future uptake and adherence of a vaginal probiotic, once proven efficacious, is determined to a large extent by its acceptability in target populations. The acceptability, in turn, depends on factors such as characteristics of the target population, characteristics of and experiences with the product, types of sexual relationships and partner support, and community perceptions.[14,15]

We conducted a clinical trial of intermittent use of two vaginal probiotics and oral metronidazole to prevent BV recurrence in Rwandan women who had been treated for BV and/or *Trichomonas vaginalis* (TV). We used qualitative and quantitative research methods to assess adherence and acceptability with vaginal probiotic use. We triangulated various sources of adherence data to obtain adherence estimates per woman for each period of intermittent product use in between study visits, and determined correlates of adherence.

METHODS

The pilot clinical trial took place from June 2015 to February 2016 at the Rinda Ubuzima research clinic in Kigali, Rwanda. The trial was a pilot trial with a modest sample size at the request of the funder. Women who had been successfully treated for BV/TV with a seven-day course of oral

For peer review only - http://bmjopen.bmj.com/site/about/guidelines.xhtml
For peer review only

metronidazole (Tricozole, Laboratory & Allied Ltd, Nairobi, Kenya) were randomised to four intervention groups (n=17 each) to prevent BV recurrence: behavioural counselling only (controls), or behavioural counselling plus intermittent use of two different vaginal probiotics or oral metronidazole for two months. The behavioural counselling included counselling on safer sex, vaginal hygiene (including discouragement of intravaginal washing), and penile hygiene (i.e. encouragement of cleansing the penis, including underneath the foreskin), because these behaviours are known to reduce BV recurrence risk somewhat.[6,16] We counselled all women in all randomisation groups because we considered it unethical to withhold this information from women at risk. Women were seen at screening, enrolment (product use initiation, if applicable), Day 7, Month 1, Month 2 (product use cessation, if applicable), and Month 6. Product efficacies were not known during the trial, and the efficacy results of the pilot trial are reported elsewhere.[17] Briefly, the vaginal probiotics did improve the vaginal environment (increased lactobacilli and reduced BV-associated bacteria) compared to counselling only, but not as much as oral metronidazole did.

Study population

Women aged 18-45 at risk of HIV/STIs (defined as having had more than one sex partner and/or having been treated for an STI and/or BV in the last 12 months) were eligible for enrolment if they were confirmed HIV-negative, non-pregnant, diagnosed with BV and/or TV, and cured after seven-day oral metronidazole treatment. Other clinical exclusion criteria were applied but were rare.[17] Women were recruited by study staff with the assistance of Community Mobilisers who had strong ties with local high-risk women (particularly sex workers).

Study products and dosing

Ecologic Femi+ (EF+; Winclove Probiotics, Amsterdam, Netherlands) is a vaginal capsule containing lyophilised lactic acid-producing bacteria. EF+ was used once per day for five days followed by thrice weekly, for two months. Gynophilus LP (GynLP; Biose, Aurillac, France) is a tablet containing the *Lactobacillus rhamnosus* Lcr35 strain. The tablet disintegrates in the vagina and forms a gel that slowly releases the probiotic bacteria. GynLP was used once every four days for two months. The first

For peer review only - http://bmjopen.bmj.com/site/about/guidelines.xhtml

dose was inserted at the clinic under direct observation of a clinician, and remaining doses were self-administered at home. Women were asked not to cleanse or insert other products into the vagina after probiotic insertion to allow the probiotics to dissolve. They were also told that they were allowed to cease probiotic use during menses, but were encouraged to continue. Intermittent metronidazole use was chosen as a positive control intervention because studies conducted in the U.S. and Kenya have shown a 30-40% reduction in BV recurrence.[18,19] Metronidazole users took 500 mg generic oral metronidazole (Laboratory & Allied ltd, Nairobi, Kenya) twice weekly for two months. The rationale for selecting these study products and their dosing schedules can be found in the manuscript describing the efficacy results of the pilot trial.[17] Participants and clinicians were not blinded.

Acceptability, adherence, behavioural, and vaginal infection knowledge assessments

Acceptability was assessed at the enrolment visit prior to product use initiation and at the Month 2 visit after the full two months of use. Adherence was assessed during the intervention period, at the Day 7, Month 1, and Month 2 visits. Sexual and other behaviours were assessed at all study visits. Participants were interviewed face-to-face in Kinyarwanda by a trained study nurse using structured questionnaires with multiple-choice questions, questions requiring a number or date, and an adherence self-rating scale (from 0-10). In between visits, participants used pictorial diary cards (online supplementary material figure 1) to record daily episodes of product use, vaginal sex, condom use, and vaginal practices. Those using study products returned the product packaging and unused products (if applicable) to their clinic visits, where they were counted by study staff. Any discrepancies between data sources were discussed with participants and the consensus assessments were recorded on the questionnaires. The adherence data based on the self-rating scale, the diary card, and the returned product packaging were triangulated by the data analyst at the data analysis stage. Additionally, 131 women were interviewed about their knowledge of vaginal infections (such as BV and STIs) using a structured questionnaire during recruitment sessions (n=61; regardless of eligibility) and at enrolment visits (n=70; this included the 68 randomised women, and two women who attended enrolment visits but turned out to be ineligible; figure 1). Women were interviewed before being counselled at study visits or before receiving information at recruitment sessions. This questionnaire

For peer review only - http://bmjopen.bmj.com/site/about/guidelines.xhtml

contained multiple-choice and open-ended questions. Responses to the open-ended questions were categorised and discussed by two different researchers until consensus about the answer categories was reached.

Four semi-structured focus group discussions (FGDs) with 7-11 participants per group (total n=38), and semi-structured individual in-depth interviews (IDIs) with four additional participants, were held. The main themes of these FGDs and IDIs were experiences with and opinions of the study products, sexual behaviour, and vaginal practices. Women randomised to the behavioural counselling only group were not approached for the FGDs and IDIs, but all other randomised participants who had completed their product use period were approached until data saturation had been achieved. The interviews were unlinked anonymous, and women used pseudonyms to enable them to talk freely despite the fact that the discussions and interviews were taped. All interviews took place between November 2015 and March 2016, were held in Kinyarwanda, recorded on tape, transcribed verbatim, and translated into English. The FGD and IDI transcripts were read and discussed by three researchers (MV, MU, and JvdW) at regular intervals. The Chief Investigator (JvdW) decided that data saturation had been met when the fourth FGD and the fourth IDI transcript had become available in March 2016.

Data analysis

The primary outcomes of this study were acceptability and triangulated adherence in women randomised to study product use. Secondary outcomes included vaginal infection knowledge of the target population more broadly, and behavioural changes (of the behaviours included in the counselling messages) in all randomised women. Questionnaire data were analysed using Stata 13 (StataCorp, College Station, TX, USA). The proportion of women with ≥80%/≥90%/100% adherence in the probiotic groups were compared by Fisher's exact tests. Changes in self-reported vaginal practices and sexual behaviours over time were tested using McNemar's test for binary outcomes, and Wilcoxon's signed-rank test for continuous outcomes. To study associations of participant characteristics with triangulated adherence, we used bivariable mixed effects models, with perfect adherence (defined as having used all doses as instructed) per interval between study visits during the

For peer review only - http://bmjopen.bmj.com/site/about/guidelines.xhtml

intervention period as the outcome, participant identification numbers as the random effect, and one participant characteristic at the time as the fixed effect. We could not determine correlates of acceptability due to limited variation in the acceptability data (reported acceptability was high throughout the trial).

The FGD and IDI transcripts were coded using NVivo 10.0 (QSR International, Melbourne, Australia) by one single researcher (MV). The discussions and interviews were semi-structured, with the above-mentioned themes and associated codes prepared a priori, as well as new elements that emerged from the data. The codes were derived from an acceptability framework that has been used in studies of vaginal products for contraception or HIV prevention.[14,15,20] Components of the framework include study population characteristics, product attributes, sexual encounter and relational attributes, and the contextual environment (e.g. community perceptions of product use).

Ethical statement

All participants provided written consent for study participation, and separate consent for participation in FGDs/IDIs. All non-married participants aged 18-20 also required parental/guardian consent per Rwandan law at the time of the study. The participants received 3 GBP per visit (in local currency) as a reimbursement for time and transport costs. Care was taken to protect participant privacy and confidentiality. The study was sponsored by the University of Liverpool, approved by the Rwanda National Ethics Committee and the University of Liverpool Research Ethics Subcommittee for Physical Interventions, and registered on ClinicalTrials.gov (NCT02459665).

Participant and public involvement

As part of the FGDs/IDIs, a subset of the enrolled participants were invited to comment on study design and experiences with the interventions. Participants were not invited to develop outcomes, interpret the results, or to contribute to the writing or editing of this document for readability or accuracy. The preliminary results of this study were discussed with 32 stakeholders during a workshop held at the Ministry of Health in Kigali, Rwanda, in December 2017. These stakeholders

For peer review only - http://bmjopen.bmj.com/site/about/guidelines.xhtml

included representatives of the Ministry of Health, the National University of Rwanda, the National Ethics Committee, local hospitals and clinics, and local non-governmental and women's organisations.

RESULTS

Baseline characteristics

We screened 176 women: bacterial STI prevalence was 31.3% and BV prevalence by Gram stain Nugent scoring was 47.9%. All 68 randomised women were treated for BV and/or TV prior to randomisation and at risk of HIV/STIs, with 93.1% reporting having exchanged sex for money and/or goods in the previous month (figure 1, online supplementary material table 1). We collected 29.93 person-years of data. Four women withdrew their informed consent during the study (for reasons unrelated to study product acceptability). None were lost to follow-up.

Adherence

Triangulated adherence was high: 17/17 (100%) of EF+ users and 13/16 (81.3%) of GynLP users used ≥80% of required doses (Fisher's exact p=0.103; table 1), and these percentages were 15/17 (88.2%) and 11/16 (68.8%) for ≥90% (p=0.225), and 10/17 (58.8%) and 8/16 (50%) for 100% of required doses (p=0.732), respectively. In comparison, these percentages were 15/17 (88.2%), 14/17 (82.4%), and 12/17 (70.6%), respectively, for oral metronidazole users. Reported reasons of non-adherence to vaginal probiotics during face-to-face interviews were 'simply forgetting' (n=9), experiencing side-effects (n=2), menses (n=2), and being away from home and having left products at home (n=1). Additional reasons for missing doses mentioned during FGDs/IDIs were being drunk (n=2) and being confused about the dosing schedule (n=2). Only one woman in the metronidazole arm reported missing doses due to experiencing side-effects. Most women in FGDs reported using all doses as instructed and finding it easy to adhere, and thought that the diary cards served as a useful reminder to use the products.

For peer review only

For peer review only - http://bmjopen.bmj.com/site/about/guidelines.xhtml

Table 1: Adherence to study interventions

Adherence to study products	Metronidazole (n=17)	EF+ (n=17)	GynLP (n=16)
Adherence Enr–D7, median % (IQR)	100 (100–100)	100 (100–100)	100 (100–100)
Adherence D7–M1, median % (IQR)	100 (100–100)	100 (100–100)	100 (91.7–100)
Adherence M1–M2, median % (IQR)	100 (100–100)	100 (100–100)	100 (92.3–100)
Overall adherence Enr–M2, median % (IQR)	100 (96.3–100)	100 (100–100)	98.3 (89.3–100)
Overall adherence Enr–M2 n (%)			
- Perfect*	12 (70.6)	10 (58.8)	8 (50.0)
- Adherence ≥90%	14 (82.4)	15 (88.2)	11 (68.8)
- Adherence ≥80%	15 (88.2)	17 (100)	13 (81.3)
Number of times menses Enr–M2 n (%)†			
- Never	7 (41.2)	4 (23.5)	2 (12.5)
- Once	6 (35.3)	5 (29.4)	4 (25.0)
- Twice	4 (23.5)	8 (47.1)	10 (62.5)
Did not use product during menses at least once n (%)			
- Yes	4 (23.5)	3 (17.6)	5 (31.3)
- NA (never had menses)	7 (41.2)	4 (23.5)	2 (12.5)
**Self-reported reasons for non-adherence‡**	**Metronidazole**	**EF+**	**GynLP**
D7: Self-reported reasons why not able to use all doses as instructed n (%)§			
- Simply forgot	0	2 (11.8)	0
- Product had side effects	0	0	1 (6.7)¶
M1: Self-reported reasons why not able to use all doses as instructed n (%)§			
- Simply forgot	1 (6.3)	1 (5.9)	1 (6.3)
- Product had side effects	1 (6.3)‖	0	1 (6.3)‡‡
- Did not like product for another reason	1 (6.3)‖	0	0
- Other	1 (6.3)**	1 (5.9)††	2 (12.5)§§
M2: Self-reported reasons why not able to use all doses as instructed n (%)§			
- Simply forgot	1 (6.3)	2 (11.8)	3 (18.8)
- Travelled and forgot to take product	1 (6.3)	0	1 (6.25)
- Other	0	1 (5.9)¶¶	1 (6.3)‖‖
D7: Participant thinks she used product correctly most of the time n (%)	17 (100)	16 (94.1)	14 (93.3)
M1: Participant thinks she used product correctly most of the time n (%)	13 (86.7)	17 (100)	11 (68.8)
M2: Participant thinks she used product correctly most of the time n (%)	15 (93.7)	16 (94.1)	14 (87.5)

*Defined as 100% of the prescribed doses used at the prescribed times after nurse review of the participant's diary card and returned used packaging and unused product.
†Number of times menses in the control group: never 2 (11.8%), once 3 (17.8%), twice 11 (64.7%), and thrice 1 (5.9%).
‡Numbers of participants per randomisation group may very slightly due to loss to follow-up. Participants with ≥90% adherence not shown.
§Multiple answers possible.
¶Participant reported vulval itching and burning when passing urine.
‖Participant reported mild gastritis and wanting to withdraw from the study anyway.
**Participant reported receiving oral metronidazole therapy for 7 days due to infection.
††Participant reported having menses twice in one month; decided to use less of her product until the next study visit.
‡‡Participant reported genital itching, genital burning, and pain during sex.
§§One participant reported missing the D7 study visit and therefore running out of supplies. Another participant reported not to have used the study product during menses (which she was allowed to do).
¶¶Participant reported being drunk and therefore forgetting to take the study product.
‖‖Participant reported taking the study product correctly but that the product came out during menses
D7, Day 7 visit; EF+, Ecologic Femi+; Enr, enrolment visit; GynLP, Gynophilus LP; IQR, inter-quartile range; M1/2, Month 1/2 visit; NA, not applicable.

For peer review only - http://bmjopen.bmj.com/site/about/guidelines.xhtml

Acceptability

Ease-of-use

No participants reported having heard about probiotics before study participation. After product use, all vaginal probiotic users reported feeling very comfortable with insertion and that insertion became easier over time. All but one woman reported inserting while lying down (online supplementary material table 2).

Bodily changes and product perception

During FGDs, several women using either vaginal probiotic reported the product (partially) "*coming out*" during the first few uses, but that this decreased after having gained experience. Many EF+ and GynLP users reported an increase in vaginal wetness, which was considered a positive attribute by most. Some women reported increased libido. For example, one EF+ user said: "*I felt a great desire to* [have] *sex again and again.*" In contrast, one metronidazole user reported a decrease in libido. Most women believed that the vaginal probiotics decreased the recurrence of symptomatic BV (our preliminary efficacy data suggest that BV incidence had in fact decreased),[17] and a few believed that they also prevented STI acquisition (the trial had insufficient statistical power to assess this).

Support

One social harm related to vaginal probiotic use was reported: a GynLP user was verbally harassed by her partner and her sister because of her study participation, and opted to withdraw her informed consent. Reports of partner, family, and community support during the FGDs/IDIs were mixed: some women reported problems with loved ones. Negative reactions from male partners were more often based on suspicions about study participation than the products themselves. One EF+ user said: "*He* [her partner] *did not accept that. He asked me to go together with him to the clinic* [a local health centre] *and check if I am not HIV-positive*." Another participant using metronidazole mentioned wanting to join the study to her husband, who forbade her to participate. However, she decided to join anyway: "*he did not know that I was using the study product, because he had refused me to join* [the]

For peer review only

For peer review only - http://bmjopen.bmj.com/site/about/guidelines.xhtml

study before… I used them [the study products] *without informing him.*" All sex workers except one stated that they had not discussed study participation with male clients.

Worries and concerns

In the FGDs, one woman reported hearing rumours prior to enrolling that vaginal products "*can damage the uterus or cause tumours in the womb.*" However, most participants thought that vaginal probiotics would be acceptable to Rwandan women. One GynLP user argued: "*They* [already] *give us vaginal pills*", by which she meant vaginal medications for yeast infections. We did not ask women explicitly whether they would be willing to pay for the products, but some women mentioned spontaneously in FGDs that they were concerned about future product availability and pricing. They hoped that probiotics would be distributed cheaply through the Rwandan *Mutuelle* public health insurance because they would otherwise be inaccessible to many women. One metronidazole user was concerned about a limited applicability of probiotics because BV is not diagnosed by laboratory testing in Rwanda: "*They do not have adequate medical instruments to test diseases, you tell the physician how* […] *you feel and by guessing the disease, he gives you at least four medications, saying that you may have trichomonas, you may have syphilis, you may have gonorrhoea* [she refers to syndromic management.[21,22]] *At health centre-level they do not have medical equipment to test diseases, meaning that they will not know who to give that* [probiotic/antibiotic maintenance therapy] *medication.*"

Vaginal practices and sexual risk-taking

At enrolment, 35/71 (49.3%) of the women reported to never use products inside the vagina, and at Month 6, this increased to 53/65 (81.5%) (OR 5.2, 95% CI 1.96-17.34; table 2). During FGDs, some women understood that vaginal washing practices may increase the risk of vaginal infection, but others did not. A participant stated: "*You get them* [i.e., vaginal diseases] *anyway… whether you wash or not*". In one FGD, 10 of 11 participants (90.9%) stated having ceased vaginal practices thanks to the study counselling. It should be noted that in contrast to many other African populations, Rwandan women use vaginal practices to increase rather than reduce vaginal lubrication. Women mentioned the

For peer review only - http://bmjopen.bmj.com/site/about/guidelines.xhtml

use of herbs (*umushishiro*), Vaseline, and oils for this purpose. Self-reported sexual risk taking by face-to-face interview did not change over time, except for a significant reduction in reported numbers of sex partners in the previous month at Month 6 compared to enrolment. No women in FGDs/IDIs mentioned adopting safer sex practices (such as consistent condom use) in response to the counselling messages. During face-to-face interviews at the Month 2 visit, 12 of 15 women (80%) who had an uncircumcised main sex partner reported asking him to regularly clean his penis in the future (online supplementary material table 2). While most women in FGDs understood that using condoms and improved penile hygiene could reduce BV rates (as shown in [6,16]), some mentioned that they found it difficult to discuss these topics with male partners. One participant stated that this is especially difficult being a sex worker: "*a man gives you his own money and you start educating him to wash!*" However, another sex worker reported refusing sex with uncircumcised clients: "*you leave him, because he has a lot* [of] *germs*". Several women reported discussing circumcision with their partners; one participant reported telling her husband: "*It is better that you do circumcision because it is a good thing… you would get a chance of not contracting diseases.*"

For peer review only - http://bmjopen.bmj.com/site/about/guidelines.xhtml

Table 2: Changes in reported vaginal cleansing practices and (sexual) behaviour between the enrolment and the M6 visit.

Self-reported sociodemographic characteristics	Enr (n=71)	M6 (n=65)	OR (95% CI)*	P value*
Reports using no products inside the vagina (other than for managing menses; all participants) n (%)	35 (49.3)	53 (81.5)	5.2 (1.96–17.34)	<0.001
Reports using no products inside the vagina (other than for managing menses; controls and metronidazole users only)† n (%)	15 (44.1)	27 (79.4)	13.0 (1.95–552.5)	0.002
Reports using water only n (%)	23 (32.4)	10 (15.4)	0.37 (0.13–0.92)	0.029
Reports using water and soap n (%)	3 (4.2)	2 (3.1)	0.67 (0.06–5.82)	1.00
Reports using paper, cloth or cotton wool n (%)	9 (12.7)	0 (0)	0.13 (0.00–0.93)‡	0.008
Reports using traditional herbs, stones, powders as vaginal cleansing practice n (%)	1 (1.4)	1 (1.5)	1.00 (0.01–78.5)‡	1.00
Mean weekly frequency of vaginal practices (95% CI)	2.15 (0.97–3.34)	0.64 (0.18–1.11)	NA	0.328
Median number of sex partners in last month at baseline or per month during follow-up period (IQR)	5 (3–16)	2 (1–4)	NA	<0.001
Any condom use reported in past two weeks (Enr) or since last study visit (M6), versus no condom use reported n (%)	64 (90.1)	60 (92.3)	1.67 (0.32–10.7)	0.727
Reports exchanging sex for money/goods in past month (Enr) or since last study visit (M6) n (%)	65 (91.5)	58 (89.2)	0.80 (0.16–3.72)	1.00

*McNemar's OR and p-value for binary variables and Wilcoxon signed-rank test p-value for continuous variables, comparing the response at M6 with the response at Enr. ORs with 95% CI were also calculated for binary pre-post data.
†N=34.
‡To enable calculation of effect measures, a zero value was replaced by 1.
CI, confidence interval; Enr, enrolment visit; IQR, inter-quartile range; M6, Month 6 visit; NA, not applicable; OR, odds ratio.

Correlates of adherence

In bivariable mixed effects models including the probiotic groups only, no participant characteristics were significantly associated with perfect adherence (table 3). However, non-significant trends were observed. Younger age (p=0.076), asking many questions at enrolment (compared to a few questions or no questions; structurally judged by a study nurse; p=0.116), having menses during the previous study interval (p=0.104), and reporting urogenital symptoms (p=0.103) were associated with a lower likelihood of perfect adherence. When including oral metronidazole users, menses was significantly associated with a lower likelihood of perfect adherence (p=0.008). There were no significant associations between randomisation group and perfect adherence.

For peer review only

For peer review only - http://bmjopen.bmj.com/site/about/guidelines.xhtml

Table 3: Participant characteristics associated with perfect adherence

Participant characteristics	EF+ and GynLP users		EF+, GynLP and oral metronidazole users	
	OR (95% CI)	P value	OR (95% CI)	P value
Randomisation group: GynLP versus EF+	0.68 (0.22–2.11)	0.505	ND	ND
Randomisation group:				
- EF+ versus metronidazole	ND	ND	0.53 (0.15–1.81)	0.308
- GynLP versus metronidazole			0.36 (0.11–1.23)	0.103
Age in years: ≥30 years versus <30	2.66 (0.90–7.82)	0.076	1.60 (0.61–4.15)	0.336
Marital status:				
- Married versus never married	0.97 (0.14–6.58)	0.976	1.17 (0.20–6.99)	0.865
- Divorced versus never married	1.18 (0.29–4.79)	0.912	1.39 (0.42–4.57)	0.586
- Widowed versus never married	ND	0.991	ND	0.990
At least some schooling versus no schooling	1.20 (0.59–2.45)	0.619	0.80 (0.22–2.95)	0.740
Number of sex partners last month: five or more versus four or less.	0.58 (0.18–1.83)	0.351	0.49 (0.17–1.37)	0.173
Exchanged sex for money/goods past month	ND	0.990	ND	0.986
Nurse reported participant asked questions at Enr				
- Yes, many versus none	0.19 (0.02–1.52)	0.116	0.15 (0.02–1.19)	0.072
- Yes, a few versus none	0.83 (0.24–2.83)	0.761	0.83 (0.27–2.57)	0.744
Had menses during study visit interval	0.41 (0.14–1.20)	0.104	0.26 (0.09–0.70)	0.008
Reported alcohol consumption during study:				
- Once or twice per week versus never	0.54 (0.14–2.12)	0.373	0.34 (0.11–1.08)	0.068
- More than twice per week versus never	0.92 (0.18–4.81)	0.920	0.81 (0.19–3.49)	0.774
Reported at least one urogenital symptom during study interval versus none	0.11 (0.01–1.56)	0.103	0.30 (0.04–2.16)	0.231
Reported at least one adverse event during study visit interval (excluding urogenital symptoms) versus none	0.43 (0.10–1.83)	0.253	0.55 (0.15–2.05)	0.371

Sociodemographic characteristics associated with perfect adherence in bivariable mixed effects models, in the enrolment–D7, D7–M1, and M1–M2 study visit intervals.

CI, confidence interval; D7, Day 7 visit; EF+, Ecologic Femi+; Enr, enrolment visit; GynLP, Gynophilus LP; M1, Month 1 visit; M2, Month 2 visit; ND, non-determinable; OR, odds ratio.

Vaginal infection knowledge

Almost all participants reported having heard of 'diseases of the vagina' and STIs before, but only 6/131 (4.6%) knew what bacteria were (table 4). The STIs most often spontaneously named (in numerical order) were HIV, gonorrhoea, and syphilis; only one participant reported having heard of BV. After having received an explanation about what BV is, only one of 131 woman reported ever having been diagnosed with BV. Most participants could name at least one cause or potential consequence of vaginal infections. Consequences wrongfully attributed to vaginal infections were cervical cancer/tumours (5/131; 3.8%), consequences to the infant such as being born with BV or congenital malformations (6/131; 4.6%), and death (4/131; 3.1%).

For peer review only

For peer review only - http://bmjopen.bmj.com/site/about/guidelines.xhtml

Table 4: Vaginal infection knowledge

	Recruitment (n=61)	Enrolment (n=70)	Total (n=131)
Median age (IQR)	32 (27–35)*	31 (27–35)	31 (27–35)
Has heard of diseases of the vagina before n (%)	60 (98.4)	70 (100)	130 (99.2)
Reports knowing what bacteria are before study n (%)	5 (8.2)	1 (1.4)	6 (4.6)
Reports having heard about STIs before study n (%)	61 (100)	70 (100)	131 (100)
If yes, spontaneously named, without probing† n (%)			
- HIV	58 (95.1)	65 (92.9)	123 (93.9)
- Gonorrhoea	58 (95.1)	65 (92.9)	123 (93.9)
- Syphilis	44 (72.1)	59 (84.3)	103 (78.7)
- Trichomoniasis	38 (62.3)	48 (68.6)	86 (65.7)
- Hepatitis	3 (4.9)	3 (4.3)	6 (4.6)
- Yeast infection	0	3 (4.3)	3 (2.3)
- BV	0	2 (2.9)	2 (1.5)
- Urinary tract infection	1 (1.6)	1 (1.4)	2 (1.5)
- Chlamydia	0	1 (1.4)	1 (0.8)
- Herpes	0	1 (1.4)	1 (0.8)
- HPV / cervical cancer	1 (1.6)	0	1 (0.8)
Reports having heard about BV before this study n (%)	1 (1.6)	0	1 (0.8)
Spontaneously reported reasons why women get vaginal disease, without probing† n (%)			
- Poor toilet hygiene	37 (60.7)	40 (57.1)	77 (58.8)
- Multiple sex partners	28 (45.9)	36 (51.4)	64 (48.9)
- After sex	25 (41.0)	30 (43.0)	55 (42.0)
- Dirty underwear	19 (31.2)	35 (50.0)	54 (41.2)
- Poor vaginal hygiene	26 (42.6)	22 (31.4)	48 (36.6)
- Poor penile hygiene of male partner(s)	4 (6.6)	17 (24.3)	21 (16.0)
- Traditional vaginal practices and washing	3 (4.9)	12 (17.1)	15 (11.5)
- New sex partner	6 (9.8)	3 (4.3)	9 (6.9)
- Use of contraception	1 (1.6)	3 (4.3)	4 (3.1)
- (Improper) use of sanitary pads or tampons	1 (1.6)	3 (4.3)	4 (3.1)
- Other	3 (4.9)‡	1 (1.4)§	4 (3.1)
- Cannot name any reasons	1 (1.6)	0	1 (0.8)
Spontaneously reported negative consequences of vaginal disease being named, without probing† n (%)			
- Foul smell from the vagina	30 (49.2)	39 (56.5)	69 (53.1)
- Difficulty getting pregnant	18 (29.5)	33 (47.8)	51 (39.2)
- Miscarriage	16 (26.2)	33 (47.8)	49 (37.7)
- Abnormal vaginal discharge	12 (19.7)	28 (40.6)	40 (30.8)
- Baby born too early	16 (26.2)	22 (31.9)	38 (29.2)
- Severe infection / fever of the woman	7 (11.5)	7 (10.1)	14 (10.8)
- Infection / fever of the newborn baby	5 (8.2)	3 (4.4)	8 (6.1)
- Itching	4 (6.6)	4 (5.8)	8 (6.1)
- Other consequences to the baby: being born with BV, congenital malformations, and others.	3 (4.9)	3 (4.4)	6 (4.6)
- Cervical cancer or tumours	2 (3.3)	3 (4.4)	5 (3.8)
- Death	4 (6.6)	0	4 (3.1)
- HIV/STIs	1 (1.6)	3 (4.4)	4 (3.1)
- Pain during intercourse	0	3 (4.4)	3 (2.3)
- Cannot name any consequence	17 (27.9)	19 (27.5)	36 (27.7)

*One missing value.
†Open-ended question. Totals may be more than 100%.
‡Participants report: "If you are infected with STIs", sharing underwear, and unprotected sex.
§Participant reports: vaginal medicine.
BV, bacterial vaginosis; HPV, human papilloma virus; IQR, interquartile range; STI, sexually transmitted infection.

For peer review only

For peer review only - http://bmjopen.bmj.com/site/about/guidelines.xhtml

DISCUSSION

Several studies of different vaginal probiotics have been conducted, some of them in sub-Saharan Africa.[10–13] However, none reported in-depth acceptability and adherence data. Our study suggests high vaginal probiotic acceptability and adherence in high-risk Rwandan women. We found no statistically significant correlates of perfect adherence, partially due to limited statistical power, but younger age, asking many questions about product use at enrolment, current menses, and reporting urogenital symptoms showed trends towards a lower likelihood of perfect adherence. Vaginal probiotics are currently unavailable on the market in most African countries, and it is important to study acceptability in different target populations to inform product development and future marketing strategies. Adherence to metronidazole was comparable to, or slightly higher than, adherence reported in previously conducted studies.[19,23]

We could not evaluate the impact of self-reported acceptability aspects on adherence because almost all women reported very high acceptability in face-to-face interviews throughout the trial. Such interviews are known to suffer from social desirability bias. However, women seemed to speak freely in the FGDs, and those data indicate that they did not have major issues with product attributes or insertion. However, some women reported difficulties due to lack of male partner support. The reported increase in vaginal wetness after probiotic insertion was not considered problematic, as lubrication during sex is preferred by most Rwandan men and women.[24] This might be different in other countries where dry sex is preferred.[25] We did find a non-significant lower adherence to GynLP compared to EF+, which might be explained by differences in formulation: GynLP forms a gel in the vagina whereas EF+ capsules merely release lyophilised bacteria. Previous research indicated high adherence to GynLP.[26] Unfortunately, the impact of these formulation differences was insufficiently probed during the FGDs; the impact of product formulation on acceptability and adherence should be investigated in future clinical trials. Participants indicated that they found the diary cards helpful in reminding them to use their products, and we believe that self-monitoring tools might indeed be helpful in maximising adherence and therefore recommend them for use in future studies.[27]

For peer review only

For peer review only - http://bmjopen.bmj.com/site/about/guidelines.xhtml

Our data suggest that counselling was partially effective in changing behaviours that increase BV risk. While these results are encouraging, it is difficult to assess to what extent they were influenced by social desirability bias. Significantly more women reported not engaging in vaginal practices at the end of the study, and most women with uncircumcised steady male partners reported having discussed penile hygiene with them. However, many women mentioned in FGDs that they found it difficult to discuss condom use and penile hygiene with male partners, especially clients. Women reduced their sexual risks only to a limited extent during follow-up, reporting a reduction in numbers of sex partners but no differences in engaging in sex work and condom use in face-to-face interviews. We did not ask women to what extent they depended on sex work for subsistence. Women who only partially depend on sex work may find it easier to negotiate with male partners.

Two probiotics-related themes that emerged from the stakeholders consultations that had not been raised by the study participants were uncertainty about long-term side effects (women in the pilot trial used the products for only two months) and whether probiotic bacteria (in this case lactobacilli) could also be delivered orally instead of vaginally. We have since conducted a systematic review, which showed that long-term safety of vaginal probiotics has not yet been evaluated.[28]

Our survey with women at recruitment sessions and enrolment visits showed that high-risk Rwandan women had heard of several STIs, but were generally unaware of BV, its causes and potential consequences, and what they can do to prevent it. Experiences with HIV show that public health interventions can only succeed if health care professionals and the public have sufficient knowledge of causes and consequences of disease.[29–31] High-risk Rwandan women (and health care professionals) should therefore be educated about BV, and vaginal probiotics studies should include counselling for all participants on vaginal diseases and how to prevent them.

Limitations

Our study had limited statistical power, and social desirability bias may have affected some of our results, as is often the case in studies of this nature. Additionally, it should be noted that product

For peer review only - http://bmjopen.bmj.com/site/about/guidelines.xhtml

efficacy, availability and cost are important determinants of acceptability, and were not evaluated in our study, although preliminary efficacy results in this study were promising.[17] We could not directly compare experiences with, and opinions about, the two different vaginal probiotics because each woman used only one product and qualitative data depth was suboptimal. In the FGDs/IDIs, it was sometimes difficult to ascertain whether participants were referring to personal experiences, or to wider community perceptions. Strengths of our study include the use of a mixed-methods approach and triangulated adherence data.

CONCLUSIONS

The prevention of BV recurrence will likely have to include several components to be successful, such as improved diagnostics, treatments, and prophylactic products (for example probiotics), but also improved information, education, and counselling messages targeted to at-risk women and their partners. The results of this study can be used to inform future product development, and to fine-tune counselling messages in future trials.

For peer review only

For peer review only - http://bmjopen.bmj.com/site/about/guidelines.xhtml

Acknowledgments We thank the study participants, the Rinda Ubuzima team and other colleagues in Rwanda and the United Kingdom who contributed, the funders of this study, as well as the Trial Steering Committee for trial oversight. We thank Biose and Winclove for the donation of study products.

Author contributions JvdW obtained the research funding and wrote the study protocol and data collection documents. AN, EL, SA, and JvdW were members of the Trial Steering Committee. SA, MU, MMU, and JvdW collected the primary data. MU and MMU performed the FGDs and IDIs. MV and JvdW developed the analytical approach and performed the statistical analyses. MV and JvdW wrote the manuscript. All authors commented on and approved the final manuscript.

Funding This work was funded by the DFID/MRC/Wellcome Trust Joint Global Health Trials Scheme as a Development Project (grant reference MR/M017443/1; grant title: "Preparing for a clinical trial of interventions to maintain normal vaginal microbiota for preventing adverse reproductive health outcomes in Africa"). Vaginal probiotics for use in the trial were donated free of charge by Winclove Probiotics (Amsterdam, The Netherlands) and Biose (formerly Probionov; Aurillac, France). The findings and conclusions in this paper are those of the authors and do not necessarily represent the views of the authors' institutions or companies, or the funder. None of the authors were paid to write this article. The corresponding author had full access to the data and had final responsibility for the decision to submit for publication.

Competing interests AN is employed by Biose (owner of trial product GynLP) and EL by Winclove Probiotics BV (owner of trial product EF+). AN has financial and/or intellectual investments in competing products. The other authors report no competing interests.

Patient consent All participants provided written informed consent for study participation, and separate written informed consent for participation in FGDs/IDIs.

For peer review only - http://bmjopen.bmj.com/site/about/guidelines.xhtml

467 **Ethics approval** The study was sponsored by the University of Liverpool, approved by the Rwanda National Ethics Committee and the University of Liverpool Research Ethics Subcommittee for Physical Interventions, and registered on ClinicalTrials.gov (NCT02459665).

471 **Data sharing statement** The data supporting the findings of this publication are retained by the corresponding author (JvdW) and will not be made openly accessible due to privacy concerns. Fully anonymised data can be made available by written request to j.vandewijgert@liverpool.ac.uk after assurance that the intended data usage is compliant with relevant ethical approvals and privacy will be maintained.

For peer review only

For peer review only - http://bmjopen.bmj.com/site/about/guidelines.xhtml

Figure footnotes

Figure 1: Flowchart of the study

*Totals to 110 reasons among 102 women because there could be more than one reason per woman.
†Reasons: outside of metronidazole treatment window (n=5), enrolment target already met (n=4), has a mental disorder (n=1), did not complete screening procedures and was subsequently lost to follow=up (n=1), withdrew consent during the screening visit because she thought the reimbursement was too low (n=1).
‡Reasons: moved away from Kigali (n=2), lost interest because symptoms resolved (n=1), and was verbally harassed by partner and sister about study participation (n=1).

Acceptability assessments were made at enrolment and at the M2 visit. Adherence assessments were made using self-rated assessments, pictorial diary cards, and returned packaging at the D7, M1, and M2 visits (after which product use was ceased). The vaginal infection knowledge survey was held at recruitment sessions in the community and at the enrolment visit. Changes in sexual risk-taking and vaginal practices were assessed at each follow-up visits and compared to answers given during the enrolment visit. All of these themes were discussed during the eight FGDs and IDIs.

BV, bacterial vaginosis; D7, day 7 visit; FGD, focus group discussion; IDI, in-depth interview; M1/2/6, month 1/2/6 visit; RU, Rinda Ubuzima; TV, *Trichomonas vaginalis*.

For peer review only

References

1 van de Wijgert JHHM, Borgdorff H, Verhelst R, *et al.* The vaginal microbiota: what have we learned after a decade of molecular characterization? *PLOS ONE* 2014;**9**:e105998. doi:10.1371/journal.pone.0105998

2 van de Wijgert JHHM, Morrison CS, Cornelisse PGA, *et al.* Bacterial vaginosis and vaginal yeast, but not vaginal cleansing, increase HIV-1 acquisition in African women. *J Acquir Immune Defic Syndr 1999* 2008;**48**:203–10. doi:10.1097/QAI.0b013e3181743936

3 van de Wijgert JHHM. The vaginal microbiome and sexually transmitted infections are interlinked: consequences for treatment and prevention. *PLOS Med* 2017;**14**:e1002478. doi:10.1371/journal.pmed.1002478

4 Li J, McCormick J, Bocking A, *et al.* Importance of vaginal microbes in reproductive health. *Reprod Sci* 2012;**19**:235–42. doi:10.1177/1933719111418379

5 Nelson DB, Hanlon AL, Wu G, *et al.* First trimester levels of BV-associated bacteria and risk of miscarriage among women early in pregnancy. *Matern Child Health J* 2015;**19**:2682–7. doi:10.1007/s10995-015-1790-2

6 Hay P. Recurrent bacterial vaginosis. *Curr Opin Infect Dis* 2009;**22**:82–6. doi:10.1097/QCO.0b013e32832180c6

7 Verstraelen H, Verhelst R. Bacterial vaginosis: an update on diagnosis and treatment. *Expert Rev Anti Infect Ther* 2009;**7**:1109–24. doi:10.1586/eri.09.87

8 Torrone EA, Morrison CS, Chen P-L, *et al.* Prevalence of sexually transmitted infections and bacterial vaginosis among women in sub-Saharan Africa: An individual participant data meta-analysis of 18 HIV prevention studies. *PLOS Med* 2018;**15**:e1002511. doi:10.1371/journal.pmed.1002511

9 Bradshaw CS, Brotman RM. Making inroads into improving treatment of bacterial vaginosis – striving for long-term cure. *BMC Infect Dis* 2015;**15**:292. doi:10.1186/s12879-015-1027-4

10 Anukam KC, Osazuwa E, Osemene GI, *et al.* Clinical study comparing probiotic *Lactobacillus* GR-1 and RC-14 with metronidazole vaginal gel to treat symptomatic bacterial vaginosis. *Microbes Infect* 2006;**8**:2772–6. doi:10.1016/j.micinf.2006.08.008

11 Ling Z, Liu X, Chen W, *et al.* The restoration of the vaginal microbiota after treatment for bacterial vaginosis with metronidazole or probiotics. *Microb Ecol* 2013;**65**:773–80. doi:10.1007/s00248-012-0154-3

12 Ngugi BM, Hemmerling A, Bukusi EA, *et al.* Effects of bacterial vaginosis-associated bacteria and sexual intercourse on vaginal colonization with the probiotic *Lactobacillus crispatus* CTV-05. *Sex Transm Dis* 2011;**38**:1020–7. doi:10.1097/OLQ.0b013e3182267ac4

13 Bradshaw CS, Pirotta M, De Guingand D, *et al.* Efficacy of oral metronidazole with vaginal clindamycin or vaginal probiotic for bacterial vaginosis: randomised placebo-

For peer review only

For peer review only - http://bmjopen.bmj.com/site/about/guidelines.xhtml

controlled double-blind trial. *PLOS ONE* 2012;**7**:e34540. doi:10.1371/journal.pone.0034540

14 van der Straten A, Montgomery ET, Cheng H, *et al.* High acceptability of a vaginal ring intended as a microbicide delivery method for HIV prevention in African women. *AIDS Behav* 2012;**16**:1775–86. doi:10.1007/s10461-012-0215-0

15 Merkatz RB, Plagianos M, Hoskin E, *et al.* Acceptability of the nestorone®/ethinyl estradiol contraceptive vaginal ring: development of a model; implications for introduction. *Contraception* 2014;**90**:514–21. doi:10.1016/j.contraception.2014.05.015

16 Liu CM, Hungate BA, Tobian AAR, *et al.* Penile microbiota and female partner bacterial vaginosis in Rakai, Uganda. *mBio* 2015;**6**:e00589. doi:10.1128/mBio.00589-15

17 van de Wijgert J, Verwijs MC, Agaba SK, *et al.* Intermittent lactobacilli-containing vaginal probiotic or metronidazole use to prevent bacterial vaginosis recurrence: safety and preliminary efficacy by microscopy and sequencing. *MedRxiv* Published Online First: 8 July 2019. doi:10.1101/19001156

18 Sobel JD, Ferris D, Schwebke J, *et al.* Suppressive antibacterial therapy with 0.75% metronidazole vaginal gel to prevent recurrent bacterial vaginosis. *Am J Obstet Gynecol* 2006;**194**:1283–9. doi:10.1016/j.ajog.2005.11.041

19 McClelland RS, Richardson BA, Hassan WM, *et al.* Improvement of vaginal health for Kenyan women at risk for acquisition of Human Immunodeficiency Virus type 1: results of a randomized trial. *J Infect Dis* 2008;**197**:1361–8. doi:10.1086/587490

20 Kestelyn E, van Nuil JI, Umulisa MM, *et al.* High acceptability of a contraceptive vaginal ring among women in Kigali, Rwanda. *PLOS ONE* 2018;**13**:e0199096. doi:10.1371/journal.pone.0199096

21 World Health Organization. Guidelines for the management of sexually transmitted infections. 2003.http://applications.emro.who.int/aiecf/web79.pdf (accessed 12 Mar 2019).

22 Binagwaho A, editor. *National guidelines for prevention and management of HIV, STIs & other blood borne infections*. Republic of Rwanda - Ministry of Health 2013. https://aidsfree.usaid.gov/sites/default/files/hts_policy_rwanda.pdf (accessed 12 Mar 2019).

23 McClelland RS, Balkus JE, Lee J, *et al.* Randomized trial of periodic presumptive treatment with high-dose intravaginal metronidazole and miconazole to prevent vaginal infections in HIV-negative women. *J Infect Dis* 2015;**211**:1875–82. doi:10.1093/infdis/jiu818

24 Veldhuijzen N, Nyinawabega J, Umulisa M, *et al.* Preparing for microbicide trials in Rwanda: focus group discussions with Rwandan women and men. *Cult Health Sex* 2006;**8**:395–406. doi:10.1080/13691050600859302

25 Low N, Chersich MF, Schmidlin K, *et al.* Intravaginal practices, bacterial vaginosis, and HIV infection in women: individual participant data meta-analysis. *PLOS Med* 2011;**8**:e1000416. doi:10.1371/journal.pmed.1000416

For peer review only - http://bmjopen.bmj.com/site/about/guidelines.xhtml

26 Dausset C, Patrier S, Gajer P, *et al.* Comparative phase I randomized open-label pilot clinical trial of Gynophilus® (Lcr regenerans®) immediate release capsules versus slow release muco-adhesive tablets. *Eur J Clin Microbiol Infect Dis* 2018;**37**:1869–80. doi:10.1007/s10096-018-3321-8

27 Safren SA, W. Otto M, Worth JL, *et al.* Two strategies to increase adherence to HIV antiretroviral medication: Life-Steps and medication monitoring. *Behav Res Ther* 2001;**39**:1151–62. doi:10.1016/S0005-7967(00)00091-7

28 van de Wijgert JHHM, Verwijs MC. Lactobacilli-containing vaginal probiotics to cure or prevent bacterial or fungal vaginal dysbiosis: a systematic review and recommendations for future trial designs. *BJOG*;**0**. doi:10.1111/1471-0528.15870

29 Glick P, Sahn DE. Changes in HIV/AIDS knowledge and testing behavior in Africa: how much and for whom? *J Popul Econ* 2007;**20**:383–422. doi:10.1007/s00148-006-0085-8

30 Peltzer K, Matseke G, Mzolo T, *et al.* Determinants of knowledge of HIV status in South Africa: results from a population-based HIV survey. *BMC Public Health* 2009;**9**:174. doi:10.1186/1471-2458-9-174

31 Fay H, Baral SD, Trapence G, *et al.* Stigma, health care access, and HIV knowledge among men who have sex with men in Malawi, Namibia, and Botswana. *AIDS Behav* 2011;**15**:1088–97. doi:10.1007/s10461-010-9861-2

For peer review only

For peer review only - http://bmjopen.bmj.com/site/about/guidelines.xhtml

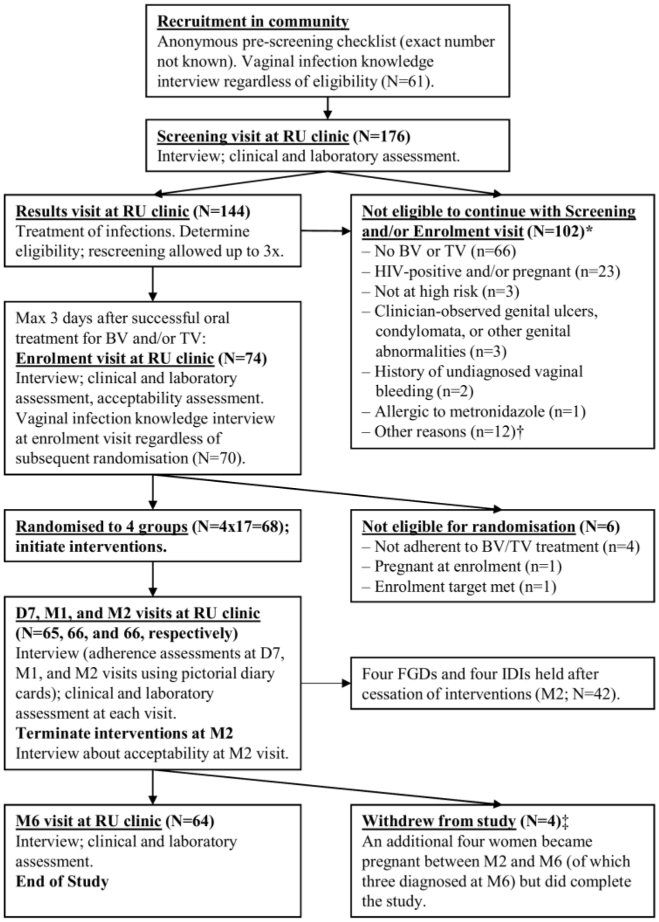

Figure 1: Flowchart of the study
*Totals to 110 reasons among 102 women because there could be more than one reason per woman.
†Reasons: outside of metronidazole treatment window (n=5), enrolment target already met (n=4), has a mental disorder (n=1), did not complete screening procedures and was subsequently lost to follow=up (n=1), withdrew consent during the screening visit because she thought the reimbursement was too low (n=1).
‡Reasons: moved away from Kigali (n=2), lost interest because symptoms resolved (n=1), and was verbally harassed by partner and sister about study participation (n=1).

Acceptability assessments were made at enrolment and at the M2 visit. Adherence assessments were made using self-rated assessments, pictorial diary cards, and returned packaging at the D7, M1, and M2 visits (after which product use was ceased). The vaginal infection knowledge survey was held at recruitment sessions in the community and at the enrolment visit. Changes in sexual risk-taking and vaginal practices were assessed at each follow-up visits and compared to answers given during the enrol visit. All this themes

were discussed during the eight FGDs and IDIs.

BV, bacterial vaginosis; D7, day 7 visit; FGD, focus group discussion; IDI, in-depth interview; M1/2/6, month 1/2/6 visit; RU, Rinda Ubuzima; TV, *Trichomonas vaginalis*.

215x279mm (300 x 300 DPI)

Supplementary Figure 1: Pictorial diary card

Date/Month	Descriptions	Monday	Tuesday	Wednesday	Thursday	Friday	Saturday	Sunday
Indicate each time you used study product								
	Used study product							
Indicate each sex act								
	Sex with condom							
	Sex without condom							
Indicate each time you washed/inserted something inside the vagina other than study product								
By washing inside, we mean inserting an entire finger inside the vaginal canal								
	Washed inside vagina with water only							
	Washed inside vagina with soap and water							
	Inserted something else (herbs, powders, etc.)							
Indicate each day of menstrual bleeding								
	Had menstrual bleeding							

The picture provided is the English translation of the pictorial card; participants received a version in Kinyarwanda.

Supplementary Table 1: Baseline characteristics of enrolled population

	**Controls (n=17)**	**Metronidazole (n=17)**	**EF+ (n=17)**	**GynLP (n=17)**
Median age (IQR)	29 (24–36)	30 (27–34)	33 (28–35)	30 (27–35)
Marital status n (%)				
- Never married	16 (94.1)	11 (64.7)	10 (58.8)	13 (76.5)
- Married	1 (5.9)	1 (5.9)	2 (11.8)	1 (5.9)
- Divorced	0	5 (29.4)	4 (23.5)	3 (17.6)
- Widowed	0	0	1 (5.9)	0
Education level n (%)				
- No schooling	5 (29.4)	3 (17.6)	3 (17.6)	3 (17.7)
- Primary school not completed	7 (41.2)	7 (41.2)	13 (76.5)	4 (23.5)
- Primary school completed	4 (23.5)	5 (29.4)	1 (5.9)	7 (41.2)
- At least some secondary school	1 (5.9)	2 (11.8)	0	3 (17.7)
Median number of sex partners last month (IQR)	5 (3–20)	5 (2–10)	3 (2–15)	3 (2–20)
Exchanged sex for money/goods past month n (%)	17 (100)	14 (82.4)	15 (88.2)	17 (100)
At least one laboratory-confirmed STI* n (%)	8 (47.1)	8 (47.1)	4 (23.5)	9 (52.9)
Median weekly frequency of washing body (IQR)	7 (7–7)	7 (7–7)	7 (7–7)	7 (4–7)
Ever washing the genitalia n (%)				
- Yes, outside only	12 (70.7)	14 (82.4)	15 (88.3)	14 (82.3)
- Yes, both inside and outside	5 (29.4)	3 (17.6)	2 (11.7)	3 (17.7)
- Yes, inside only	0	0	0	0
If reports washing inside, median weekly frequency (IQR)	14 (7–16)	14 (14–14)	11 (7–14)	7 (3–12)

*Chlamydia, gonorrhoea, and/or syphilis.
EF+, Ecologic Femi+; Enr, enrolment visit; GynLP, Gynophilus LP; IQR, inter-quartile range; M2, Month 2 visit; STI, sexually transmitted disease.

For peer review only

Supplementary Table 2: Acceptability of interventions

Acceptability of study products at Enr	Controls (n=17)	Metronidazole (n=17)	EF+ (n=17)	GynLP (n=17)
Nurse reports having explained intervention to participant in detail n (%)	17 (100)	17 (100)	17 (100)	17 (100)
Nurse reports participant asked questions n (%)*				
- Yes, a few	6 (35.3)	2 (11.8)	11 (64.7)	11 (64.7)
- Yes, many	0	0	0	2 (11.8)
First dose applied† under supervision n (%)	NA	17 (100)	17 (100)	17 (100)
Median number of attempts participant made until successful application (IQR)	NA	NA	1 (1–1)	1 (1–1)
Participant seemed comfortable with the insertion after these attempts, according to study nurse n (%)	NA	NA		
- Yes, very			17 (100)	16 (94.1)
- Yes, somewhat			0	1 (5.9)
**Acceptability of study products at M2**				
Self-reported usual time of insertion n (%)				
- Before going to sleep	NA	NA	17 (100)	15 (100)‡
- After bathing in the morning			0	0
Level of comfort with vaginal insertion after 2 months of use, self-reported n (%)	NA	NA		
- Very comfortable			17 (100)	15 (100)‡
- Somewhat comfortable			0	0
Reported insertion becoming easier over time n (%)	NA	NA	17 (100)	15 (100)‡
Reported manner of insertion§ n (%)				
- While lying down	NA	NA	17 (100)	14 (93.3)‡
- While squatting			1 (5.9)	1 (6.7)
**Acceptability of penile hygiene intervention at M2**				
Reports having told main sex partner to regularly clean the penis, including underneath the foreskin n (%)¶				
- Yes	3 (17.7)	3 (18.8)	3 (17.6)	3 (18.8)
- No, because he is circumcised	10 (58.8)	9 (56.2)	6 (35.3)	5 (31.3)
- No, other reason	1 (5.9)	0	1 (5.9)	1 (6.3)
If yes, response by the main partner n (%)‖				
- He said that he would do so in the future	2 (66.7)	1 (33.3)	1 (33.3)	1 (33.3)
- He said that he already does this	1 (33.3)	1 (33.3)	0	1 (33.3)
- He said that he is not interested	0	1 (33.3)	2 (66.7)	1 (33.3)

*One missing value.
†Whether oral insertion (oral metronidazole group) or vaginal insertion (Ecologic Femi+ and Gynophilus LP groups).
‡N=15 due to participants withdrawing informed consent.
§Multiple answers possible; hence totals can be more than 100%.
¶Women with no main sex partner not included.
‖N=3 in all four groups.
EF+, Ecologic Femi+; Enr, enrolment visit; GynLP, Gynophilus LP; IQR, inter-quartile range; M2, Month 2 visit; NA, Not applicable.

For peer review only

STROBE Statement—Checklist of items that should be included in reports of ***cohort studies***

	Item No	Recommendation	Reported on page #
**Title and abstract**	1	(*a*) Indicate the study's design with a commonly used term in the title or the abstract	1
		(*b*) Provide in the abstract an informative and balanced summary of what was done and what was found	2
**Introduction**			
Background/rationale	2	Explain the scientific background and rationale for the investigation being reported	4
Objectives	3	State specific objectives, including any prespecified hypotheses	4
**Methods**			
Study design	4	Present key elements of study design early in the paper	4
Setting	5	Describe the setting, locations, and relevant dates, including periods of recruitment, exposure, follow-up, and data collection	4
Participants	6	(*a*) Give the eligibility criteria, and the sources and methods of selection of participants. Describe methods of follow-up	4,5
		(*b*) For matched studies, give matching criteria and number of exposed and unexposed	NA
Variables	7	Clearly define all outcomes, exposures, predictors, potential confounders, and effect modifiers. Give diagnostic criteria, if applicable	6
Data sources/ measurement	8*	For each variable of interest, give sources of data and details of methods of assessment (measurement). Describe comparability of assessment methods if there is more than one group	6
Bias	9	Describe any efforts to address potential sources of bias	6
Study size	10	Explain how the study size was arrived at	NA (Primary outcomes paper)
Quantitative variables	11	Explain how quantitative variables were handled in the analyses. If applicable, describe which groupings were chosen and why	6
Statistical methods	12	(*a*) Describe all statistical methods, including those used to control for confounding	6,7
		(*b*) Describe any methods used to examine subgroups and interactions	6,7
		(*c*) Explain how missing data were addressed	NA (indicated in tables if missing)
		(*d*) If applicable, explain how loss to follow-up was addressed	NA
		(*e*) Describe any sensitivity analyses	NA
**Results**			
Participants	13*	(a) Report numbers of individuals at each stage of study—eg numbers potentially eligible, examined for eligibility, confirmed eligible, included in the study, completing follow-up, and analysed	Page 8, Figure 1
		(b) Give reasons for non-participation at each stage	8, Figure 1
		(c) Consider use of a flow diagram	Figure 1
Descriptive data	14*	(a) Give characteristics of study participants (eg demographic, clinical, social) and information on exposures and potential confounders	8
		(b) Indicate number of participants with missing data for each variable of	8 (missing

For peer review only

		interest	data in footnotes in table, if applicable)
		(c) Summarise follow-up time (eg, average and total amount)	8
Outcome data	15*	Report numbers of outcome events or summary measures over time	9
Main results	16	(*a*) Give unadjusted estimates and, if applicable, confounder-adjusted estimates and their precision (eg, 95% confidence interval). Make clear which confounders were adjusted for and why they were included	9
		(*b*) Report category boundaries when continuous variables were categorized	Table 1
		(*c*) If relevant, consider translating estimates of relative risk into absolute risk for a meaningful time period	NA
Other analyses	17	Report other analyses done—eg analyses of subgroups and interactions, and sensitivity analyses	Page 12 and beyond
**Discussion**			
Key results	18	Summarise key results with reference to study objectives	Page 18
Limitations	19	Discuss limitations of the study, taking into account sources of potential bias or imprecision. Discuss both direction and magnitude of any potential bias	Page 19
Interpretation	20	Give a cautious overall interpretation of results considering objectives, limitations, multiplicity of analyses, results from similar studies, and other relevant evidence	Page 18, 19
Generalisability	21	Discuss the generalisability (external validity) of the study results	Page 18, 19
**Other information**			
Funding	22	Give the source of funding and the role of the funders for the present study and, if applicable, for the original study on which the present article is based	Page 21

*Give information separately for exposed and unexposed groups.

Note: An Explanation and Elaboration article discusses each checklist item and gives methodological background and published examples of transparent reporting. The STROBE checklist is best used in conjunction with this article (freely available on the Web sites of PLoS Medicine at http://www.plosmedicine.org/, Annals of Internal Medicine at http://www.annals.org/, and Epidemiology at http://www.epidem.com/). Information on the STROBE Initiative is available at http://www.strobe-statement.org.

For peer review only - http://bmjopen.bmj.com/site/about/guidelines.xhtml

For peer review only
